# Comprehensive Density Functional and Kinetic Monte Carlo Study of CO_2_ Hydrogenation on a Well-Defined Ni/CeO_2_ Model Catalyst: Role of Eley–Rideal Reactions

**DOI:** 10.1021/acscatal.3c05336

**Published:** 2024-01-30

**Authors:** Pablo Lozano-Reis, Pablo Gamallo, Ramón Sayós, Francesc Illas

**Affiliations:** Departament de Ciència de Materials i Química Física & Institut de Química Teòrica i Computacional (IQTCUB), Universitat de Barcelona, C. Martí i Franquès 1, 08028 Barcelona, Spain

**Keywords:** Ni/CeO_2_, Eley−Rideal reactions, metal−support interactions, methane selectivity, CO_2_ hydrogenation, kinetic Monte Carlo, DFT calculations

## Abstract

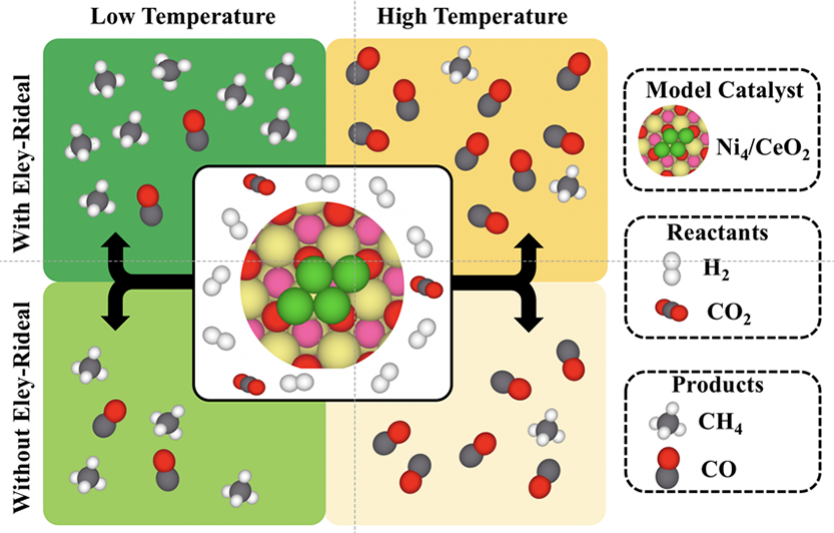

A detailed multiscale study of the mechanism of CO_2_ hydrogenation on a well-defined Ni/CeO_2_ model catalyst is reported that couples periodic density functional theory (DFT) calculations with kinetic Monte Carlo (kMC) simulations. The study includes an analysis of the role of Eley–Rideal elementary steps for the water formation step, which are usually neglected on the overall picture of the mechanism, catalytic activity, and selectivity. The DFT calculations for the chosen model consisting of a Ni_4_ cluster supported on CeO_2_ (111) show large enough adsorption energies along with low energy barriers that suggest this catalyst to be a good option for high selective CO_2_ methanation. The kMC simulations results show a synergic effect between the two 3-fold hollow sites of the supported Ni_4_ cluster with some elementary reactions dominant in one site, while other reactions prefer the another, nearly equivalent site. This effect is even more evident for the simulations explicitly including Eley–Rideal steps. The kMC simulations reveal that CO is formed via the dissociative pathway of the reverse water–gas shift reaction, while methane is formed via a CO_2_ → CO → HCO → CH → CH_2_ → CH_3_ → CH_4_ mechanism. Overall, our results show the importance of including the Eley–Rideal reactions and point to small Ni clusters supported on the CeO_2_ (111) surface as potential good catalysts for high selective CO_2_ methanation under mild conditions, while very active and selective toward CO formation at higher temperatures.

## Introduction

The continuous use of carbon-rich fossil fuels has dramatically increased the atmospheric amounts of carbon dioxide producing devastating effects on our ecosystem. In order to reverse this situation, CO_2_ valorization has emerged as a low-cost strategy to reduce the environmental impact related to carbon dioxide and, at the same time, generate value-added chemicals. In this regard, the power to gas^1,2^ (PtG) technology has gained attraction as a promising option to absorb and exploit surplus renewable energies using CO_2_ as a feedstock. The PtG concept is based on using excess of energy produced from renewable sources for carrying out water splitting to further use the produced H_2_ for CO_2_ hydrogenation toward different chemicals. Among the different possible processes, the power to methane (PtM) technology^3^ provides an interesting chemical route to produce methane that is further used as a fuel, now in a circular way. Regarding this reaction, Ni-based catalysts are commonly used due to their relatively high activity and its economic viability in comparison to catalysts using other noble metals.^4,5^ In particular, Ni/CeO_2_ catalysts have been shown to exhibit superior catalytic activity than other Ni-based catalysts.^6−9^

In the past years, considerable effort has been devoted to understand the intricacies that make Ni/CeO_2_ catalysts so active for the CO_2_ methanation reaction.^6−18^ Unfortunately, there is still not a clear consensus around this issue, and different hypotheses have been proposed. Among them, researchers have focused on the effect of support, of particle size and morphology, and of the strong metal–support interaction (SMSI) just to name a few. Regarding the support, Martin et al.^7^ studied the CO_2_ hydrogenation reaction over different Ni-based catalysts and reported that the highest activity and methane selectivity correspond to the Ni/CeO_2_ catalyst. They attributed the increase of activity and selectivity to the quite small Ni nanoparticles that were present on these systems. Similarly, Le et al.^9^ studied the CO and CO_2_ hydrogenation reaction over several catalysts consisting on Ni nanoparticles supported over different metal oxides. They reported the highest catalytic activity for Ni/CeO_2_, and again attributed this boost of activity to the small size of Ni nanoparticles that were present in that system. These conclusions point to the nanoparticle size as a key defining the final catalytic activity and selectivity, which probably stimulated other research groups to focus their attention on the particle size effect for the Ni/CeO_2_ catalyst. Even though several studies have focused on the Ni nanoparticle size, different conclusions have been reached, and there is still not a clear consensus on how the nanoparticle size affects the catalytic activity. Thus, some studies claim that large nanoparticles are more active, while other studies argue the opposite. For instance, Lin et al.^10^ studied three systems with Ni nanoparticles supported on CeO_2_ featuring different sizes (i.e., 2, 4, and 8 nm) and found that the larger Ni nanoparticles were more active. They attributed the higher activity of the larger particles to their ability to dissociate H_2_ thus producing a higher amount of H adatoms that further hydrogenate interfacial species to methane. Similar results were obtained by Winter et al.^11^ and Zheng et al.^12^ who also reported higher catalytic activity and selectivity for the systems containing larger nanoparticles. Interestingly, in both works, they observed a rapid increase in the methane selectivity when increasing the nanoparticle size. On the other hand, Lin et al.,^13^ in a very recent article, studied different Ni/CeO_2_ systems with Ni nanoparticle sizes ranging from 9 to 11 nm and reported that the system with the smallest nanoparticles, although rather large particles with size ∼9 nm, had the highest catalytic activity and selectivity toward methane. They attributed the boost of activity to a large number of interfacial oxygen vacancies as a response of the large metal–support interaction that smallest nanoparticles feature. Similarly, Rui et al.^14^ prepared two different Ni/CeO_2_ systems following two different preparation methods and observed that the smaller the nanoparticle, the larger the metal–support interaction, which was translated into more oxygen vacancies and a higher catalytic activity.

Moreover, some results from the literature points to a correlation between the strong metal–support interaction and the catalytic activity rather than a correlation between the Ni nanoparticle size and the catalytic activity. Precisely, in a very recent work, Pu et al.^15^ studied the effect of SMSIs for three different Ni/CeO_2_ systems with nanoparticles of ∼5 nm but with different metal–support interactions due to the level of the nanoparticles encapsulation. They suggested that the SMSI effect is closely related to the encapsulation and the larger the encapsulation, the larger the metal–support interaction, which was translated into a large amount of oxygen vacancies where CO_2_ can be activated with the concomitant increase of the catalytic activity. Combining theoretical modeling and experiments, several studies claim that small Ni clusters or nanoparticles supported on CeO_2_ exhibit large metal–support interactions together to high catalytic activity for a variety of reactions such as the dry reforming of methane,^19−22^ direct conversion of methane to methanol,^23^ water dissociation,^24^ methane steam reforming reaction,^25^ water–gas shift (WGS) reaction,^26^ and CO_2_ methanation.^27^ Regarding theoretical studies dealing with CO_2_ hydrogenation reaction to methane over Ni/CeO_2_, there is, to the best of our knowledge, only the work of Zhang et al.^27^ In their density functional theory (DFT) study, they propose different mechanisms for the CO_2_ hydrogenation reaction and suggest that methane is formed via the reverse water–gas shift (RWGS) reaction followed by the CO-hydrogenation pathway.

However, for such CO_2_ hydrogenation reaction involving a complex network of elementary steps and several active sites, one cannot always rely on the static picture offered by the typical DFT-based energy or free energy profiles to accurately describe the system evolution under real working conditions. In these cases, it is necessary to couple DFT calculations to some kinetic modeling techniques as we have very recently shown for the RWGS reaction on Ni/TiC systems.^28^ Precisely, the goal of the present work is to reach a thoroughly description of the CO_2_ hydrogenation reaction over a Ni/CeO_2_ model system, where both support composition and crystal structure and supported Ni atomic structure are well-defined. To this end, we couple a very detailed study of all elementary steps to kinetic Monte Carlo (kMC) simulations. This multiscale approach provides compelling evidence of the role of each part of the model catalyst and unravel the main mechanism that governs the overall reaction, highlighting the unexpected role of the Eley–Rideal (ER) elementary steps on the final activity and selectivity.

## Models and Methods

### Ni/CeO_2_ Structural Model

**Figure 1:**
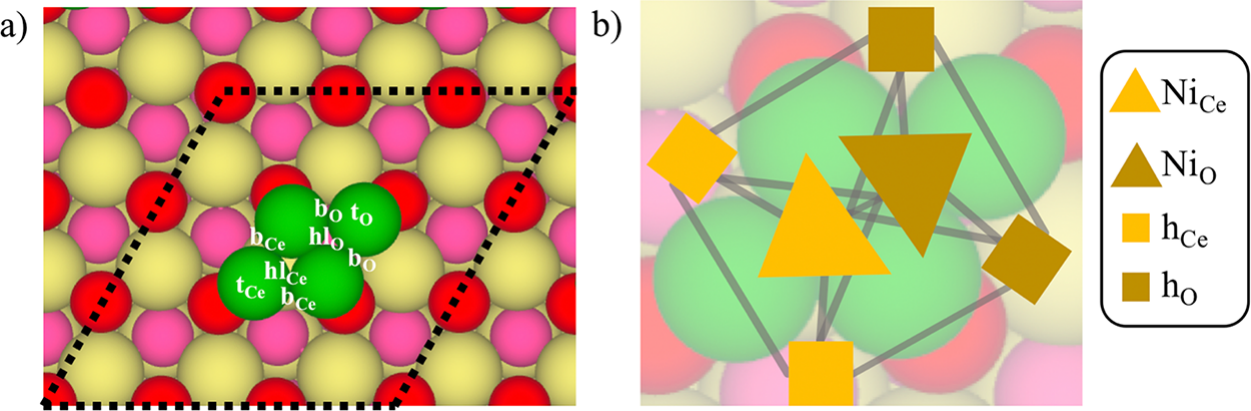
(a) Surface model of the Ni_4_/CeO_2_ system used for the DFT calculations. Green, pale yellow, red, and pink stand for Ni, Ce, uppermost O, and subsurface O atoms, respectively. t_Ce_/t_O_, b_Ce_/b_O_, and hl_Ce_/hl_O_ stands for top, bridge, and 3-fold hollow sites for the site type “Ce” and “O”, respectively. (b) Lattice model representing the supported Ni_4_ cluster used for the kMC simulations. Triangles represent the Ni hollow sites, while squares represent the hydrogen reservoir sites in which H and H_2_ can adsorb. Gray lines depict the connectivity between sites.

The Ni/CeO_2_ system is modeled by a flat Ni_4_ cluster supported on a slab model of the stoichiometric CeO_2_(111) surface. The slab model consists of an appropriate 3 × 3 CeO_2_(111) supercell built from the calculated fluorite structure of ceria bulk with an equilibrium lattice parameter of *a*_o_ = 5.445 Å. The slab contains a total of nine atomic layers, or three O–Ce–O trilayers. A Ni_4_ cluster is deposited on top of the surface with its equilibrium structure as shown in Figure 1a. A 13 Å vacuum width between periodically repeated slabs has been included in order to avoid spurious interactions between the periodic replicas in the perpendicular direction to the surface. Regarding the choice of the supported Ni cluster, one has to realize that, in practice, many different supported particles coexist. Thus, when aiming at comparing to experiment one should be aware that supported metal clusters may present several near-degenerate structural isomers that can contribute to the catalytic reaction,^29−31^ specially under operating conditions and that even the high-energy states can contribute to the final production as very recently was shown by Xia et al.^32^ From the modeling point of view, one needs to select a model that can be representative enough while accepting that small variations are possible depending on the choice. In the present work, we selected a flat Ni_4_ cluster even though it has been shown that both flat and tetrahedral Ni_4_ isomers supported on CeO_2_(111) present similar stability.^33^ The particular choice of the flat Ni_4_ cluster is because it has more atoms in direct contact with the support, thus mimicking also the situation that can be encountered in slightly larger clusters. Besides, a flat supported cluster maximizes the electronic metal–support interactions that are responsible for increased catalytic activity. These arguments are further confirmed by data in the literature showing values of the CH_4_ and H_2_O dissociation energy barriers for the supported Ni_4_ planar cluster supported on CeO_2_ lower than those corresponding to the tetrahedral one, which points to a higher catalytic activity for the planar cluster.^21,24,34^ Note that similar results have been reported for Cu/CeO_2_ and Ni/TiC systems, in which 2D clusters are more active than their 3D counterparts.^20,34−36^ With these considerations in mind, the main goal of the present work is to study the complex CO_2_ hydrogenation reaction on a well-defined model catalyst that can be representative of experiments involving small Ni clusters supported on CeO_2_(111). For these reasons, comparison to experiment should be taken as qualitative rather than quantitative, yet with a correct unveiled chemistry.

### DFT Calculations

To characterize the energetics of the CO_2_ hydrogenation reaction for the Ni_4_ cluster supported on the CeO_2_ (111) surface model, periodic spin-polarized DFT calculations have been carried out by means of the Vienna Ab-initio Simulation Package (VASP) code^37−39^ using the frozen-core augmented (PAW)^40^ method to describe the interaction between the atomic cores and the valence electron density. All calculations have been done using the PBE+U formalism involving the PBE functional^41^ and a value of 4.5 eV for the Hubbard U-like term, which is included to correctly represent the Ce 4f states.^42,43^ In addition, we have included the Grimme D3^44^ term to capture the effect of dispersion in the calculated energies. The Ce oxidation state is estimated by analyzing its local magnetic moment which, in turn, is estimated from the spin density. In particular, values of 0 and ∼1 μ_B_ are found for the Ce^4+^ and Ce^3+^ ions consistent with the occupation of the *f* states of 0 and ∼1, respectively. For the Ni atoms, the oxidation state per atom is calculated as the total number of electrons transferred to the CeO_2_ support, estimated through a Bader analysis,^45^ divided by the number of Ni atoms that are in direct contact with the support. Note that this procedure has been extensively used in similar systems to calculate the oxidation state of both Ce and Ni atoms.^21,25,33^ The particular choice of the Ni_4_ cluster is to have a representative model of a small flat cluster exhibiting electronic metal–support interactions.^23,27,34,46,47^ In all calculations, the three lowermost layers (one O–Ce–O trilayer) have been kept fixed at their bulk position to provide an adequate bulk environment to the top surface layers. The atomic structure of the six uppermost layers of the Ni_4_ cluster and that of the different adsorbed species have been allowed to fully relax during the calculations. The Brillouin zone has been sampled with a (3 × 3 × 1) *k*-point mesh using the Monkhorst–Pack scheme,^48^ and a cutoff energy of 415 eV has been used for the plane wave basis expansion. The electronic energy criterion has been selected to 10^–5^ eV, while a value of 0.01 eV Å^–1^ has been used for the ionic relaxation criterion.

Transition state (TS) structures have been located using the climbing-image nudged elastic band (CI-NEB) method.^49,50^ To generate the initial image guesses, the image-dependent pair potential procedure^51^ has been used as implemented in the atomic simulation environment (ASE) package.^52^ The located TS structures have been properly characterized by vibrational frequency analysis to ensure that all TSs have only the desired imaginary frequency corresponding to the reaction coordinate. Frequency calculations have also been performed for all the adsorbed and coadsorbed structures ensuring that they correspond to minima on the potential energy surface (PES). The calculated frequencies have been used to calculate the zero-point energy (ZPE) contribution of the different structures as well as to calculate the vibrational partition functions, which are required for the calculation of preexponential factors that are necessary to compute the transition probabilities (usually termed as rate constants) used in the kMC simulations. Note that low-frequency modes below a cutoff value of 6.9 meV have been set to this cutoff value in order to approximate the entropy from a pseudorotational/pseudotranslational degree of freedom, as previously done in ref (53). The energy of the gas-phase species has been calculated by placing a single molecule in an asymmetric box of dimension 9 × 10 × 11 Å^3^ and considering the Γ point only. Finally, the reaction energies (Δ*E*_0,r_) and energy barriers (Δ*E*_0_^≠^) including the ZPE contribution have been calculated as
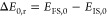
1
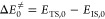
2where *E*_FS,0_, *E*_IS,0_, and *E*_TS,0_ are the total energy of the final state, initial state, and TS, respectively, also including the ZPE contribution.

### kMC Simulations

In order to gain insights into the system evolution under real working conditions, kMC simulations have been carried out using the graph-theoretical kMC approach^54^ combined with cluster expansion Hamiltonians^55,56^ as implemented in the Zacros software.^54,55^ The kMC lattice (Figure 1b) is built so as to mimic the Ni_4_ cluster used for the DFT calculations (Figure 1a) and consists of a nonperiodic custom grid of 6 points representing surface sites, where the different species can adsorb, desorb, react, or diffuse. A total of 4 different sites have been considered, as described next. Two different coarse-grained (i.e., Ni_Ce_ and Ni_O_) sites have been used to represent the 3-fold hollow sites of the Ni_4_ cluster. We have used two different types of sites because the two hollow sites of the Ni_4_ cluster are not exactly the same as one has a Ce atom underneath (i.e., Ni_Ce_), while the other has an O atom below (i.e., Ni_O_). These differences translate into different adsorption energies depending on the site the species adsorbs. Note that each coarse-grained site includes the top, bridge, and 3-fold hollow site as shown in Figure 1a. Moreover, we have considered that species adsorbed on the top, bridge, or 3-fold hollow site occupy a single coarse-grained site, which avoids the use of many multidentate species. Finally, to avoid the use of a “hard sphere” model for small adsorbates such as H and H_2_, we have used a special hydrogen reservoir site,^28,57^ in which these two species can adsorb. As for the Ni sites, we have considered two different types of hydrogen reservoirs (i.e., h_Ce_ and h_O_). The presence of two nonidentical sites involves considering the energetics of the different adsorbed species and the energetics of the possible elementary reactions twice, each one for each site. Moreover, to correctly define the reactivity, we have considered that if the two Ni sites are occupied, hydrogenation reactions can only occur if both species are on the same type of site (i.e., both species in Ce sites or both species in O sites). For instance, let us imagine a situation in which CH, O, and H species are adsorbed onto Ni_O_, Ni_Ce_, and h_O_, respectively. In this case, the H adatom cannot react with the oxygen atoms as it is blocked by the CH, and it can only react with CH or diffuse to the h_Ce_ site. This condition is used to correctly capture blocking effects. Finally, mapping the DFT calculations, we have considered that all species are monodentate and occupy a single site with the only exception of CO_2_ and COOH that we have considered as bidentate species occupying both the Ni_Ce_–Ni_O_ or Ni_O_–Ni_Ce_ sites, as well as H_2_ that occupies the h_Ce_–h_O_ sites.

**Figure 2:**
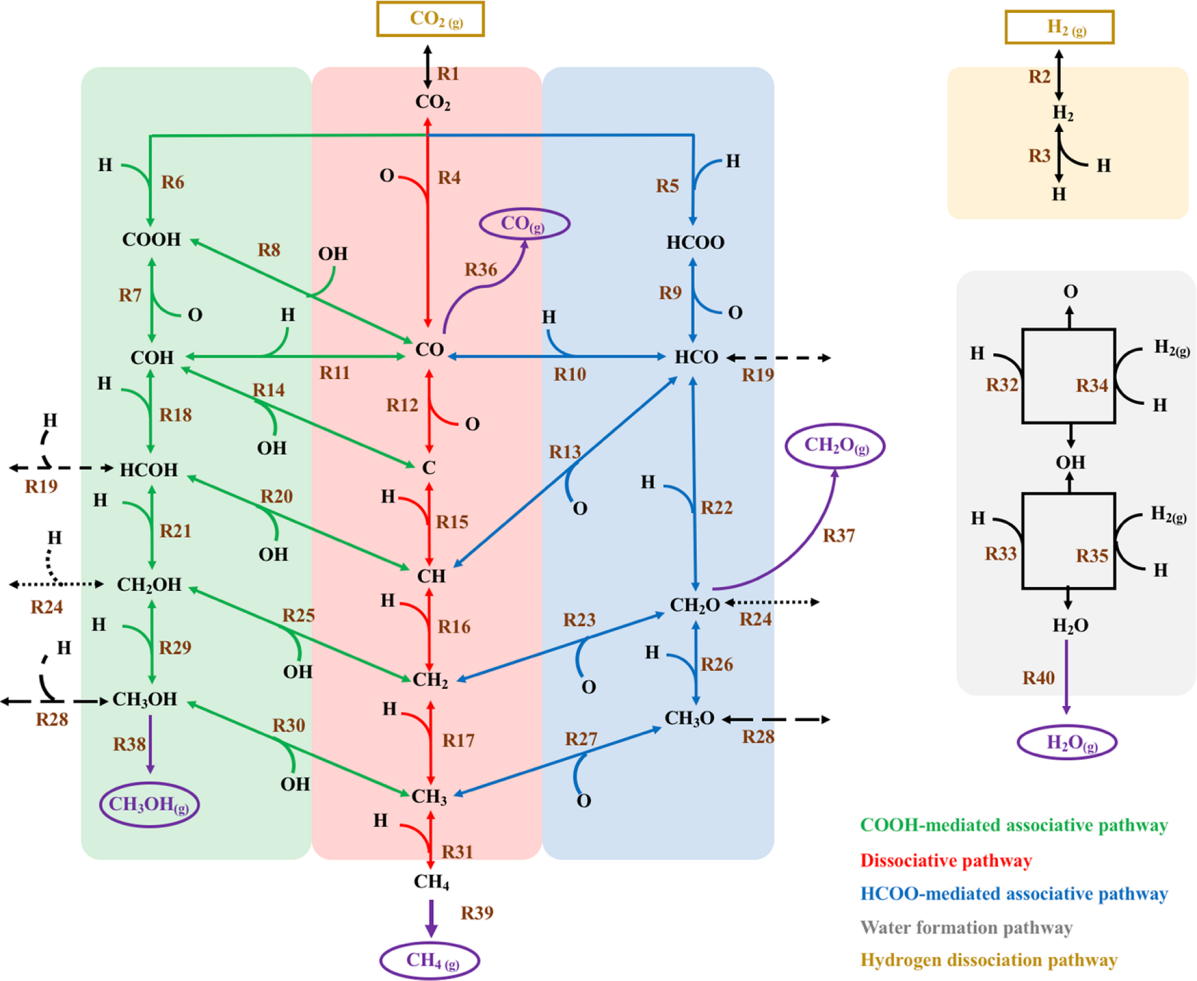
Reaction network proposed for the CO_2_ hydrogenation reaction. The dissociative pathway, COOH-mediate pathway, HCOO-mediated pathway, hydrogen dissociation pathway, and water formation pathway are shown in red, green, blue, pale yellow, and gray, respectively. Black dotted/dashed lines are for elementary steps that interconnect different pathways. Dark yellow and purple stand for reactants and products, respectively. Reversible steps are represented by double arrows.

The reaction network involves a total of 98 reversible reactions, including adsorption, desorption, surface reaction, and diffusion steps. Besides, we have also considered some ER steps for the hydrogenation of O (i.e., R34) and OH species (i.e., R35) as shown in Figure 2. The decision to only include the ER hydrogenation reactions for water formation via the hydrogenation of O and OH species is because, in our lattice model, in which only two sites are considered, water formation is a very important step as it leaves a free site in which the CH_*x*_ species can be produced, which are further hydrogenated to methane (vide infra). Nevertheless, even if the inclusion of some extra ER reactions for some hydrogenation steps could slightly change the results, the main conclusions will remain untouched. Note that, as explained earlier, reactions are considered twice as the adsorbed species can be either at the Ni_Ce_ site or at the Ni_O_ site. Moreover, for some hydrogenation reactions, we have considered two different possibilities: (i) the H adatom and the other species are at the same type of site (i.e., both at Ce or O type of sites) and (ii) the H adatom and the other species are at different type of sites (i.e., one at Ce and the other at O type of sites or vice versa). The cluster expansion used in our model includes pairwise lateral interactions between all possible reactant/product pairs as well as between all other relevant species. The cluster expansion includes first-nearest neighbors two-body terms as well as some second-nearest neighbors two-body terms. Overall, it contains 41 one-body terms and 93 two-body terms as summarized in Tables S1 and S2 in the Supporting Information. For very fast and quasi-equilibrated processes, the transition probabilities have been scaled by a factor α <1 to speed up the kMC simulations while ensuring that these processes are still very fast and quasi-equilibrated. This pragmatic solution has been extensively and successfully applied in several previous studies.^28,53,57−60^

To study the CO_2_ hydrogenation reaction over the Ni cluster, we have considered an initial mixture of CO_2_ and H_2_ continuously impinging on a pristine surface in which different processes can take place and where the products formed are considered to desorb without allowing readsorption. The working conditions are chosen as in the experiments of Zheng et al.,^12^ which are P(H_2_) = 0.528 bar and P(CO_2_) = 0.132 bar and temperatures ranging from 483 to 563 K. Additional simulations for a temperature of 563 K and different partial pressures are also performed in order to study the partial orders of reaction for the RWGS reaction and the methanation, also known as the Sabatier reaction. Moreover, for all the different working conditions, we have run the simulations including some ER steps and without including them to gain insights about the role of this kind of reactions. Finally, in order to better sample the configurational space, we have run 5 different simulations, which only differ from each other in the sequence of random numbers, and the macroscopic magnitudes are given as the average of the five different replicas.

## Results and Discussion

### DFT Results

#### Ni_4_/CeO_2_ Interaction

The Ni_4_ cluster adsorbs above the oxygen atoms of the CeO_2_(111) uppermost layer, adopting a structure that is reminiscent of that of the Ni(111) facet (see Figure 1a). Interestingly, the cluster adsorption triggers a charge transfer between the metal cluster and the surface so that two formally Ce^4+^ atoms are reduced to Ce^3+^ atoms, while the Ni atoms are partially oxidized to Ni^0.5+^, a clear indication of metal–support interactions, which is in agreement with previous results for similar Ni/CeO_2_ systems.^25,33,46,47^ Precisely, the metal–support interactions affect the electronic structure of the Ni atoms of the Ni adcluster, making them different from those of the Ni(111) surface. In general, this is beneficial for the adsorption and activation of the different species with a concomitant increase of the catalytic activity as shown later. Therefore, we have studied the adsorption of the possible reactants and products of the CO_2_ hydrogenation reaction (see Figure 2) over the Ni cluster sites with results summarized in Table 1, where some data from our previous study of the CO_2_ hydrogenation reaction on Ni(111) have been included for comparison.^53^ Note that, in general, bifunctional catalysts such as Ni/CeO_2_ contain three different regions with different energetics, namely, the metal region, the support region, and the interface region that lies in between the two former regions. In this study, we have restricted our calculations to the Ni cluster region for several reasons. First, we want to understand the effect of the metal–support interactions, which is thought to increase the metal activity. Moreover, the pristine (un)reduced CeO_2_ support has been shown to be inactive for CO_2_ hydrogenation^12,18^ so that one can safely discard this region. Finally, adsorption energies for CO_2_ on the Ni cluster are larger than those at the interface region,^27^ and a noticeable CO_2_ adsorption energy is a necessary step for its catalytic hydrogenation.

**Table 1:** Adsorption Energy for the Different Reactants and Products of the CO_2_ Hydrogenation Reaction on the Supported Ni Cluster at the Most Stable Adsorption Sites Along with Already Published Data for the Ni(111) Surface^a^

species	*E*_ads,0_/eV	
	this work	Ni (111) ref (53)
CO_2_	–1.46 (Ni_Ce_), −1.51 (Ni_O_)	–0.16
CO	–2.47 (Ni_Ce_), −2.33 (Ni_O_)	–1.61
CH_2_O	–2.10 (Ni_Ce_), −2.06 (Ni_O_)	–0.58
CH_3_OH	–0.89 (Ni_Ce_), −0.91 (Ni_O_)	–0.36
CH_4_	–0.26 (Ni_Ce_), −0.26 (Ni_O_)	–0.13
H_2_O	–0.73 (Ni_Ce_), −0.75 (Ni_O_)	–0.26
H_2_	–0.63 (h_Ce_ – h_O_)	0.00

a Note that all values include the ZPE term.

As shown in Table 1, the most favorable sites for the different stable gas-phase species are, in general, the two different 3-fold hollow sites. The adsorption energies are similar for the two different sites. Let us start by comparing our values in Table 1 for the Ni cluster with reported values for similar systems. For instance, the present CO_2_ adsorption energies of −1.51 and −1.46 eV at both 3-fold hollow sites are similar to the values reported by Alvarez-Galvan et al.^46^ and Zhang et al.^27^ of −1.26 and −1.64 eV, respectively, lying in between them. A careful comparison between present and previous values helps in understanding the origin of the differences in adsorption energy. For instance, we have found a CO_2_ structure that is bent to the surface; hence, featuring a higher interaction with the surface that can explain the higher adsorption energy, we have found with respect to the study of Alvarez-Galvan et al.^46^ Regarding the latter study by Zhang et al.,^27^ the differences are smaller—less than 0.2 eV—and can be attributed to the different computational code used and the different methodology used in the calculations. Moreover, our values include the ZPE term, while it does not seem to be included in the commented previous works. Alvarez-Galvan et al.^46^ also reported a molecular H_2_ adsorption energy of −0.95 eV, which again is slightly larger than the present value of −0.63 eV. The difference is clearly due to the different Ni atom in which H_2_ adsorbs as well as to the lack of the ZPE term. For instance, in this study, the molecular H_2_ adsorption energy with and without the ZPE term is −0.63 and −0.75 eV, respectively, the latter being closer to the value reported by Alvarez-Galvan et al.^46^ Moreover, additional deviations can arise from the inclusion or not the contribution of long-range dispersion interactions, which have been included in our calculations via the Grimme’s D3 approach, while it seems that this is not the case in the literature values. Finally, Lustemberg et al.^34^ reported adsorption energies of −0.93 and −0.24 eV for H_2_O and CH_4_, respectively, which agree with our reported values. Again, we attribute the differences between our values and the reported ones due to the inclusion or not of the ZPE term, our values being −0.75 and −0.82 eV for H_2_O and −0.26 and −0.28 eV for CH_4_ when including or not the ZPE term. In general, the present values are close enough to those reported in the literature, and we suggest that further differences are due to small details in the methodology used in the calculations. Next, we compare the values obtained for the Ni clusters and the ones reported for the Ni(111) surface.^53^ From Table 1 one can clearly see how the metal–support interactions along with differences in the Ni atomic coordination lead to larger adsorption energies with the former, implying a considerable charge transfer, being likely to dominate. For instance, CO_2_ and H_2_ molecules physisorb on the Ni(111) surface, while they are clearly adsorbed on the supported Ni cluster. As these two molecules are the reactants of the CO_2_ hydrogenation reaction, higher adsorption energies would, in principle, favor the catalytic activity of the Ni cluster suggesting Ni clusters to be more active than the Ni(111) surface. Nevertheless, it is important to point out that only from the adsorption energy values it is not possible to conclude which system will be more catalytically active as small energy barriers are required for the reaction to proceed (vide infra). Regarding the desired product (CH_4_), the adsorption energy on the Ni cluster is also larger than on Ni(111). Nevertheless, in both cases, CH_4_ has small adsorption energy, which would favor methane desorption. Focusing on the other possible products, namely CO, CH_2_O, and CH_3_OH, we see again that the support effect leads to an increase of the adsorption energies of these species. Interestingly, a larger adsorption energy of the different products would, in principle, benefit CH_4_ formation. In fact, as reported for the Ni(111) facet,^53^ one of the problems that makes it inactive for methane formation is the low CO adsorption energy compared with other competitive reactions, favoring CO desorption rather than subsequent reactions. Therefore, a large CO adsorption energy would, in principle, favor methane selectivity, pointing to the Ni cluster being more selective than the Ni(111) surface.

#### Reactivity of the Ni_4_/CeO_2_ Model

We have shown that metal–support interactions strongly increase the adsorption capacity of the Ni cluster compared with the Ni(111) facet. Nevertheless, as explained earlier, to gain insights about the catalytic behavior of a given catalyst one must also evaluate the energy barriers of the different competing elementary reactions. In Table 2, we summarize the reaction energy and energy barrier for some elementary steps of the CO_2_ hydrogenation reaction (see Figure 2). For simplicity, we have only selected the most relevant reactions for our discussion, while information regarding all the studied elementary reactions can be found in Table S3. For simplicity, we restrict the discussion to the elementary steps at the Ni_Ce_ site, but similar conclusions can be extracted focusing on the Ni_O_ site. First, we start by comparing our calculated values with those already published for the same system. Comparing our values (see Table S3) with those from Zhang et al.,^27^ one can rapidly see a huge difference in the energy barriers reported by these authors and the present ones. In general, they reported significantly larger energy barriers for several elementary reactions with barriers ranging from 2 to 4 eV. This fact contrasts with the present calculated values that are generally smaller with nearly vanishing barriers for some steps with the largest one being 2.18 eV. We suggest that these differences are due to the different initial and final states used and to the different computational methodology used for the calculations. In fact, Alvarez-Galvan et al.^46^ reported a CO_2_ dissociation energy barrier of 0.75 eV, which nicely agrees with our calculated value of 0.77 eV. Moreover, they also found a barrierless H_2_ dissociation reaction also in line with our results. Likewise, Lustemberg et al.^34^ reported values that are in good agreement with our values. Note that for some reactions, these authors considered the presence of some spectator species, while for others, the presence of the spectators is not considered, which clearly changes the energetics of the studied elementary reaction as shown for the CH_4_ dissociation reaction. For a better comparison with the present values, we focus on the situation without spectator species. These authors studied CH_4_ and H_2_O dissociation and CH_3_OH formation reactions, reporting energy barriers of 0.14, 0.41, and 1.40 eV, respectively, which closely resemble our reported values of 0.14, 0.35, and 1.33 eV, respectively, as shown in Tables 2 and S3 for the reactions R31_NiCe,n_, R33_NiCe,n_, and R28_NiCe_, respectively. Interestingly, the very small CH_4_ dissociation energy barrier contrasts with the larger values reported for Ni/CeO_2_ systems containing single Ni atoms^20^ or 3D Ni clusters,^21,25^ while are similar to other M_4_/CeO_2_ systems containing flat metallic clusters (M = Co, Pt, and Ni),^47^ which points to systems similar to the Ni_4_/CeO_2_ model as potential good candidates for methane conversion reactions. Note, however, that CH_4_ dissociation will compete with the very small CH_4_ desorption energy (i.e., 0.26 eV), the latter being detrimental to methane conversion reactions. Finally, the very good agreement between our calculated values and these two works points to a proper definition of our model system while questioning the results of Zhang et al.^27^

**Table 2:** Reaction Energies (Δ*E*_0,r_) and Forward and Reverse Energy Barriers (Δ*E*_0,f_^≠^, Δ*E*_0,r_^≠^) for the Selected Elementary Reactions Including the ZPE for the Ni_4_/CeO_2_ System and for the Ni(111) Surface^53^ for Comparison^a^

ID	reaction	Δ*E*_0,r_/eV		Δ*E*_0,f_^≠^		Δ*E*_0,r_^≠^/eV	
		this work	ref (53)	this work	ref (53)	this work	ref (53)
R1_NiCe_	CO_2, (g)_ + *_NiCe_ + *_NiO_ ⇌ CO_2, NiCe – NiO_^**^	–1.46	–0.16	0.00	0.00	1.46	0.16
R2	H_2, (g)_ + *_hCe_ + *_hO_ ⇌ H_2, hCe – hO_^**^	–0.63	0.00	0.00	0.00	0.63	0.00
R3	H_2, hCe – hO_^**^ ⇌ H_hCe_^*^ + H_hO_^*^	–0.70	–0.33	0.00	0.26	0.70	0.59
R4_NiCe_	CO_2, NiCe – NiO_^**^ ⇌ CO_NiCe_^*^ + O_NiO_^*^	–0.70	–0.57	0.77	0.86	1.47	1.43
R5_NiCe_	CO_2, NiCe – NiO_^**^ + H_hO_^*^ ⇌ HCOO_NiCe_^*^ + *_hO_ + *_NiO_	–0.07	0.04	0.39	1.05	0.45	1.01
R6_NiCe_	CO_2, NiCe – NiO_^**^ + H_hO_^*^ ⇌ COOH_NiCe – NiO_^**^ + *_hO_	0.22	0.49	1.20	1.33	0.98	0.84
R9_NiCe_	HCOO_NiCe_^*^ + *_NiO_ ⇌ HCO_NiCe_^*^ + O_NiO_^*^	0.23	0.85	0.65	1.39	0.42	0.54
R10_NiCe_	CO_NiCe_^*^ + H_hO_^*^ ⇌ HCO_NiCe_^*^ + *_hO_	0.60	1.21	0.90	1.42	0.30	0.21
R12_NiCe_	CO_NiCe_^*^ + *_NiO_ ⇌ C_NiCe_^*^ + O_NiO_^*^	1.03	1.84	1.52	2.98	0.49	1.15
R13_NiCe_	HCO_NiCe_^*^ + *_NiO_ ⇌ CH_NiO_^*^ + O_NiCe_^*^	0.31	–0.07	0.64	1.10	0.33	1.17
R16_NiCe,n_	CH_NiCe_^*^ + H_hCe_^*^ ⇌ CH_2, NiCe_^*^ + *_hCe_	–0.38	0.30	0.03	0.63	0.41	0.34
R17_NiCe,n_	CH_2, NiCe_^*^ + H_hCe_^*^ ⇌ CH_3, NiCe_^*^ + *_hCe_	–0.53	–0.11	0.09	0.57	0.62	0.68
R22_NiCe_	HCO_NiCe_^*^ + H_hO_^*^ ⇌ CH_2_O_NiCe_^*^ + *_hO_	0.29	0.26	0.46	0.71	0.17	0.45
R23_NiCe_	CH_2_O_NiCe_^*^ + *_NiO_ ⇌ CH_2, NiO_^*^ + O_NiCe_^*^	–0.39	–0.40	0.68	0.96	1.07	1.37
R31_NiCe,n_	CH_3, NiCe_^*^ + H_hO_^*^ ⇌ CH_4, NiCe_^*^ + *_hO_	0.77	–0.30	0.91	0.79	0.14	0.96
R36_NiCe_	CO_NiCe_^*^ ⇌ CO_(g)_ + *_NiCe_	2.47	1.61	2.47	1.61	0.00	0.00
R37_NiCe_	CH_2_O_NiCe_^*^ ⇌ CH_2_O_(g)_ + *_NiCe_	2.10	0.58	2.10	0.58	0.00	0.00
R39_NiCe_	CH_4, NiCe_^*^ ⇌ CH_4, (g)_ + *_NiCe_	0.26	0.13	0.26	0.13	0.00	0.00

a For reactions in which two possible hydrogen attacks are considered, the *f* and *n* subscripts stand for the H atom being at the site that is far or near the attacking species, respectively. For instance, *f* stands for situations in which H and the other species are at h_O_/Ni_Ce_ or h_Ce_/Ni_O_, respectively, and *n* stands for situations in which H and the other species are at h_Ce_/Ni_Ce_ or h_O_/Ni_O_, respectively. The * and ** symbols stand for monodentate (one site) or bidentate (two sites) adsorbed species, respectively.

Let us now evaluate the effect of the metal–support interaction on the energy barriers of the different elementary reactions and the possible mechanism that drive the overall reaction by comparing the values obtained for the Ni cluster and the values previously reported for the Ni(111) facet. Comparing the calculated values and the values reported in ref (53) (see Tables 2 and S3) it can be seen that, as a result of the metal–support interaction, the energy barriers of the different elementary steps are reduced, which could be beneficial for catalytic purposes. Focusing on some of the reactions, one can clearly spot from Table 2 that H_2_ dissociation would be more favorable on the supported Ni cluster than on the extended surface, as for the first system it is a barrierless reaction while it has an energy barrier of 0.26 eV on Ni(111). Moreover, H_2_ adsorption is more favorable on the supported Ni cluster, which also points to a higher activity. Comparing the different routes to CO_2_ conversion, namely, direct CO_2_ dissociation, COOH formation, and HCOO formation reactions, similar barriers are obtained for the two former reactions (although slightly smaller for the supported Ni cluster), while a large difference is observed for HCOO formation with values of 0.39 and 1.05 eV for the Ni cluster and the Ni(111) surface, respectively. The values for the Ni cluster suggest that the most probable reaction will be the HCOO (formate) formation followed by the CO_2_ dissociation, which clearly opens the HCOO-mediated pathway as a possible route for either CO or methane formation. This contrast with the kMC results obtained for the Ni(111) surface in which the CO_2_ dissociation pathway and, to a lower extent, the COOH-mediated pathway were the active pathways, while the formate path was inactive.^53^ Let us assume now that CO has been produced and then we evaluate the possible pathways for methane formation that start with COH (carbon-hydroxyl) formation, CO dissociation, and HCO (formyl) formation reactions. The former has similar energy barriers for both systems being the largest one among the three different reactions, hence the less probable. Regarding CO dissociation, one can spot a huge change in the energy barriers with values of 1.52 and 2.98 eV for the supported cluster and the extended surface, respectively. This suggests that direct CO dissociation could compete with other routes on the supported cluster, while this reaction is very unlikely on Ni(111). However, for the supported cluster, even with a reasonable energy barrier, the reaction is highly endothermic and thus, thermodynamically impeded. For HCO formation, one can also see a decrease in the energy barrier and, at the same time, a decrease in the endothermicity of the reaction. Precisely, one of the drawbacks for methane formation on the Ni(111) facet has been reported to be the high endothermicity of the HCO formation reaction, so that whenever HCO is formed it rapidly dissociates to CO, which further desorbs.^53^ At the supported cluster, the lower endothermicity added to the highest CO adsorption energy could be paramount for the methane formation. After HCO is formed, it can also dissociate to CH or hydrogenated to CH_2_O, with energy barriers of 0.64 and 0.46 eV for the supported cluster and values of 1.10 and 0.71 eV for the Ni(111) surface, respectively. Again, both reactions have lower energy barriers for the supported cluster thus being more probable to be executed and more competitive with respect to the HCO dissociation to CO. Regarding, CH_2_O dissociation to CH_2_, the energy barrier on the supported cluster is lower than on the Ni(111) surface, which opens another route for methane formation. Finally, the CH_*x*_ species can be easily hydrogenated to form methane on both surfaces, albeit with lower values for the cluster.

To sum up, we have shown that metal–support interaction induces an effect on the supported Ni cluster resulting in a decrease of the energy barriers of some of the relevant elementary steps and, at the same time, increases the adsorption energy of some side products, suggesting the possible formation of methane. From the DFT analysis, it appears that the most probable pathway for methane formation will be a combination of the CO formation—either via the HCOO-mediated pathway and, to a lower extent, the CO_2_ dissociation pathway—followed by the CO hydrogenation to HCO, which can either dissociate to CH or being hydrogenated to CH_2_O, that further dissociates to CH_2_ that finally, can be further hydrogenated to CH_4_. Also, the quite large CO and CH_2_O adsorption energies suggest high methane selectivity. Our proposed mechanism agrees with the one proposed by Zhang et al.,^27^ with the solely exception of the CO formation, that they suggested it is formed via the COOH-mediated pathway. Nevertheless, from the DFT picture only it is not possible to obtain any firm conclusion about the catalytic activity and selectivity and on the actual mechanism that governs the reaction. Moreover, as shown by Lustemberg et al.,^34^ for CH_4_ dissociation, neighboring spectator species can change the reactivity due to the adsorbate–adsorbate interactions. At this point, one may think of reporting and analyzing the Gibbs free energy profile of the proposed mechanism as a way to better understand the catalytic reaction. However, one should be aware that this type of picture, while very useful, stands for situations in which reactants, intermediates, and products do not interact with each other. These situations are easily encountered for systems involving large terraces and small coverage. In the present case, however, the reacting species are in close proximity implying large adsorbate–adsorbate interactions, which are properly accounted for in the kMC simulations via the cluster expansion and will be hardly to reproduce and extremely costly, ultimately leading to a series of Gibbs free energy diagrams. Therefore, to reach a more accurate and realistic description, the evolution of the system under real working conditions must be considered. To this end, in the next section, we couple the DFT calculations with kMC simulations that naturally accounts for the effect of neighboring species and provide insights about the activity, selectivity, and mechanistic aspects at the pressure and temperature conditions of interest.

### kMC Results

#### Outcome of the kMC Simulations

To reach a deep understanding of the system evolution under real working conditions, kMC simulations have been carried out. This allows us to gain insights about the role of the different sites and different elementary steps, such as ER reactions, on the global reaction mechanism, the catalytic activity, and the selectivity toward CH_4_, the latter being conditioned by the competition between the partial CO_2_ hydrogenation to CO via the RWGS reaction and the complete CO_2_ hydrogenation to CH_4_ via the Sabatier reaction. Besides, we compare our values with results reported in the literature for Ni/CeO_2_ systems,^12,16,17^ mainly focusing on the experimental results reported for a system in which small Ni nanoparticles or clusters are likely to be present although, unfortunately, the structure of the CeO_2_ support is different.^12^ Before describing such a comparison, one must be aware that there are some differences between our model and the experimental catalysts. More in detail, the present model involved a supported flat Ni_4_ cluster, while in the experiments, an ensemble of Ni clusters or nanoparticles of different sizes and morphologies is present. Moreover, our study focuses only on the reactivity of the supported Ni cluster on a stoichiometric, well-defined, CeO_2_(111) surface, while in experiments, the support is likely to exhibit oxygen vacancies and other active sites may be present at the interface providing additional active sites where CO_2_ can adsorb and further react. Furthermore, additional deviations from the experimental values may arise from errors in the computed DFT energies, the kMC method itself, or the truncation to two-body terms in the cluster expansion. Despite these limitations, our multiscale study gives useful insights about the catalytic activity and selectivity of small supported Ni clusters that are likely to be present in Ni/CeO_2_ systems with low Ni loading. Finally, and just as a reminder for the reader, we want to point out that the ER reactions we have included in the kMC simulations involve the formation of water via hydrogenation of O and OH species.

Table 3 summarizes the CO, CH_4_, and total turnover frequencies (TOFs) as well as the selectivity toward methane for the scenarios in which ER reactions are included or not. From Table 3, one can spot that, for both scenarios, the higher the temperature, the higher the total TOF and the lower the CH_4_ selectivity. The former is not surprising as the higher the temperature, the higher the system energy and the easier to overcome the different energy barriers with the parallel increase in the catalytic activity. The latter could be explained because CO is an intermediate species of the complete CO_2_ hydrogenation to CH_4_ (vide infra); thus, the higher the temperature, the more probable the CO desorption and the lower the selectivity toward methane. Therefore, a temperature increase translates into higher CO production, while this is not always the case for CH_4_. Interestingly, the highest CH_4_ formation is observed at 543 K for the simulations with(out) the ER reactions, respectively. This shows that, above 543 K, the temperature has a higher effect on the CO desorption than on the other elementary steps necessary for methane formation pointing to this temperature as a limit for a higher CH_4_ production. Note, however, that the highest CH_4_ selectivity is observed for the lowest temperature, which points out that working under this condition will result in high selective methane formation, even if the overall production is smaller than at other temperatures. From Table 3, one can also see similar trends for the different reported magnitudes for the simulations with(out) the ER steps, while higher absolute values are found when ER steps are included. Let us now compare our results with the experimental values reported by Zheng et al.^12^ We have found a qualitative good agreement with the experimental values for the system with the lowest Ni loading that point that at 563 K CO is the major product. In comparison, we have found a lower CH_4_ selectivity (4.4 vs 21.1%) and a larger total TOF (4.318 vs 0.187 molec site^–1^ s^–1^). Note that, for a better comparison, we have adapted their reported values to our units. This is done just by using the Ni atomic mass, Avogadro’s number, and considering that each site contains two Ni atoms. Note also that the CO TOF is normally larger than the CH_4_ one; hence, it is not surprising that our total TOF is higher than the experimental value as we have found a lower CH_4_ selectivity that translates into a higher influence of CO on the total TOF. In fact, for a temperature of 523 K in which the methane selectivity (for the simulations with ER steps) is similar to the experimental value at 563 K, our total TOF is 1.191 molec site^–1^ s^–1^ (see Table 3), which better agrees with the experimental value. Nevertheless, we suggest that the higher CO production we observe is due to the very repulsive adsorbate–adsorbate interactions (see Table S2) present in such a small supported cluster in which steric effects induce species to be in less stable sites, increasing the repulsive interactions between species. This translates into a CO adsorption energy lower than the one reported in Table 1, which turns into an easier CO desorption. Regarding the experimental system, it is likely that even the system with the lowest Ni loading contains large clusters or small nanoparticles in which species can better adapt to the system with the concomitant decrease of the adsorbate–adsorbate interactions that can explain the lower experimental activity and higher CH_4_ selectivity. Let us now compare our results with the ones reported by Xie et al.^17^ for different Ni/CeO_2_ systems containing different amounts of oxygen vacancies, frustrated Lewis pairs, and different sizes and morphologies for the Ni nanoparticles and CeO_2_ support. In contrast to our results, they reported a methane selectivity of ∼95% for a temperature range of 423 to 673 K, clearly higher than our values of 73.1 to 4.4% for a temperature range of 483 to 563 K. Again, we attribute the large CH_4_ selectivity experimentally observed due to the large Ni nanoparticles, and the effect of the oxygen vacancies and frustrated Lewis pairs present on these systems. Regarding the CH_4_ TOF, they reported values between 0.16 and 0.24 molec site^–1^ s^–1^ for a temperature of 498 K, which nicely agree with our CH_4_ TOF at 503 K of 0.178 molec site^–1^ s^–1^ pointing to small Ni clusters as interesting options for low-temperature methane formation. Therefore, from their conclusions and our results, one can argue that Ni/CeO_2_ catalysts combining small Ni clusters with interfacial oxygen vacancies and frustrated Lewis pairs are likely to be promising catalysts for low-temperature methane formation. To sum up, the present results suggest that flat small Ni clusters supported over the CeO_2_(111) surface are potential good candidates for highly active and selective CO formation at high temperatures while pointing to be suitable catalysts for active and selective methane formation under mild conditions.

**Table 3:** Total, CO, and CH_4_ TOFs and Methane Selectivity for the Simulations Including the ER Steps (W/ER) and without Them (Wo/ER) at Five Different Temperature Conditions and with P(H_2_) = 0.528 bar and P(CO_2_) = 0.132 bar for All the Simulations^a^

*T*	total TOF		CO TOF		CH_4_ TOF		CH_4_ selectivity	
	W/ER	Wo/ER	W/ER	Wo/ER	W/ER	Wo/ER	W/ER	Wo/ER
483	0.141 ± 0.013	0.065 ± 0.006	0.038 ± 0.006	0.029 ± 0.003	0.103 ± 0.009	0.036 ± 0.005	73.1 ± 2.9	55.0 ± 4.3
503	0.380 ± 0.018	0.212 ± 0.014	0.202 ± 0.018	0.162 ± 0.017	0.179 ± 0.007	0.050 ± 0.006	47.0 ± 2.7	23.9 ± 3.8
523	1.191 ± 0.027	0.755 ± 0.047	0.912 ± 0.028	0.698 ± 0.048	0.280 ± 0.007	0.057 ± 0.010	23.5 ± 0.8	7.5 ± 1.4
543	2.892 ± 0.015	1.993 ± 0.025	2.587 ± 0.021	1.936 ± 0.023	0.305 ± 0.010	0.057 ± 0.005	10.6 ± 0.4	2.9 ± 0.2
563	4.516 ± 0.053	3.556 ± 0.074	4.318 ± 0.063	3.524 ± 0.076	0.199 ± 0.017	0.033 ± 0.003	4.4 ± 0.4	0.9 ± 0.1

a The TOF units are in molec site^–1^ s^–1^, while the CH_4_ selectivity is given in percent. Note that the methane selectivity is calculated as CH_4_ selectivity = (CH_4_ TOF/total TOF) × 100. Note that the present values are calculated as the mean value of 5 different kMC simulations for each working condition and including the standard deviation.

#### Mechanistic Insights of CO and CH_4_ Formation

**Figure 3:**
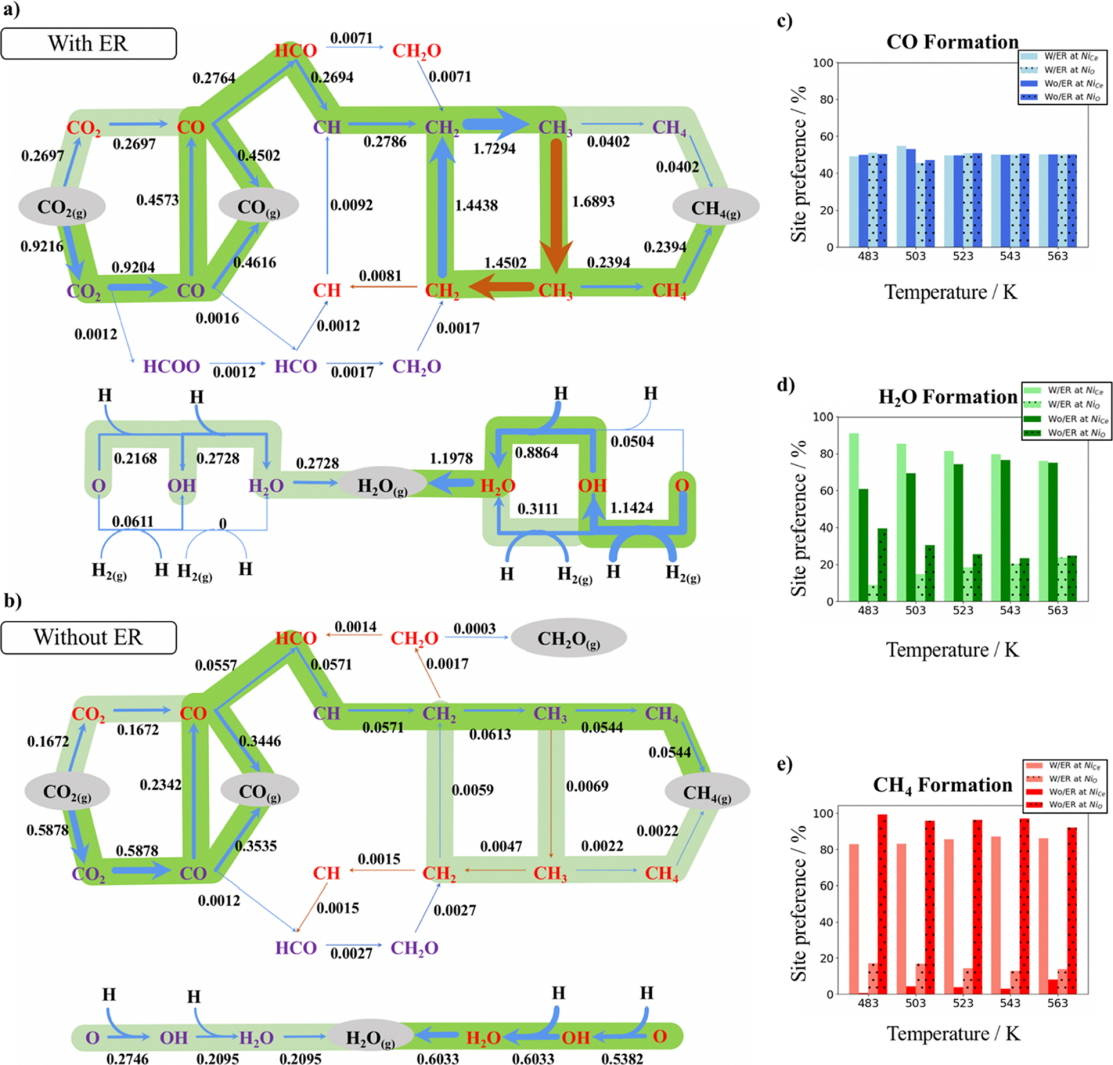
Schematic representation of the different executed events at a temperature of 523 K and P(H_2_) = 0.528 bar and P(CO_2_) = 0.132 bar for simulations with and without ER reactions in (a,b), respectively. Purple and red letters stand for species adsorbed on the Ni_O_ and Ni_Ce_ sites, respectively. Black numbers represent the net number of executed processes as the average of 5 different kMC simulations in units of events site^–1^ s^–1^. Blue arrows stand for events executed from right to left and from bottom to up, while brown arrows stand for events executed in the opposite directions. The size of the arrows represents the weight of the elementary step. Green and light green colors highlight the most important and secondary pathways, respectively. (c–e) Site preference formation of CO, H_2_O, and CH_4_ on the Ni_Ce_ and Ni_O_ sites for the simulations with and without the ER reactions, in blue, green, and red colors, respectively. Light colors stand for the simulations with ER reactions, while dark colors stand for the simulations without ER reactions. Segments with no texture represent desorption on the Ni_Ce_ site, while segments with “.” texture stand for desorption on the Ni_O_ site.

We now examine in detail the role of the ER steps on the overall reaction mechanism, the activity, and methane selectivity. We chose to carry out the analysis at 523 K because at this temperature we have found a reasonable CH_4_ selectivity and a high CH_4_ production. Note that, regarding the dominant mechanism, no important changes are observed for the other working conditions. Figure 3a,b shows a schematic representation of the net executed processes for the simulations with and without the ER reactions, respectively. The event frequency plots at the different temperatures and different sites are included in Figure S1. As shown in Figure 3a,b, there is a clear synergy between the two different sites, in which some reactions are dominant in one site while other reactions happen on the other site. For both scenarios, CO_2_ has a large adsorption energy at the Ni_O_ site (see Table 1) although a nonnegligible amount of CO_2_ also adsorbs at Ni_Ce_. Once CO_2_ is adsorbed, it can dissociate or be hydrogenated to HCOO, which further dissociates to produce HCO. From Figure 3a,b, one can spot that CO_2_ dissociation is the dominant reaction, and only a very few HCOO moieties are formed when the ER reactions are included. As shown in Table S3, HCOO formation has a lower energy barrier than CO_2_ dissociation, which points to the former reaction to be more probable. However, the former reaction is less exothermic than CO_2_ dissociation. Moreover, once HCOO is formed, it has to dissociate to HCO, which is an endothermic reaction, that tends to go backward to form HCOO again. In fact, the CO_2_ + H → HCOO → HCO + O total process is executed more times than the direct CO_2_ dissociation (see Figure S1) but due to the endothermic nature of the last reaction, the overall process goes backward, and the net balance is for CO_2_ dissociation. Interestingly, the HCOO path is slightly observed when including the ER reactions because in this situation it is easier for the adsorbed O species to be hydrogenated through R34 and R35 avoiding the recombination of HCO + O to HCOO and allowing HCO to generate other species. Regarding CO_2_ dissociation, it can be seen in Figure 3a,b that it is predominant on the Ni_O_ sites, producing CO and O on Ni_O_ and Ni_Ce_ sites, respectively. At this point, there are only two possibilities for the reaction to continue: CO desorption or O hydrogenation to water that further desorbs. Interestingly, Figure 3c shows that CO desorbs in a similar amount from both sites, which points to water being formed, in general, first, hence, leaving a free Ni_Ce_ site in which CO can diffuse and further desorbs (among other possible reactions). On the contrary, if CO desorbs first, one should expect a CO desorption ratio similar to the one for the CO_2_ dissociation reaction at the different sites, which is not the case as shown in Figure 3c. In this regard, Figure 3d also points to water being formed first as it is formed mostly on the Ni_Ce_ site. Moreover, this can also explain why the activity is lower when the ER reactions are not included as water is more difficult to be formed in this situation and the system needs more time to produce water, which is a necessary step for the latter CO diffusion and desorption with the simultaneous decrease on the catalytic activity.

Now, we focus on the CH_4_ formation starting from CO. Once CO is formed, the most probable reaction is CO hydrogenation to HCO. This reaction is more probable at the Ni_Ce_ site than at the Ni_O_ one due to the slightly lower energy barrier associated with the former. This is similar to HCO dissociation which is also favored at the Ni_Ce_ site (see Table S3). This fact promotes CO diffusion from Ni_O_ to Ni_Ce_, where some CO desorbs and some CO hydrogenates to HCO that further dissociates to CH and O at Ni_O_ and Ni_Ce_, respectively (see Figure 3a,b). Note that there is also a very small amount of HCO that is hydrogenated to CH_2_O that further dissociates to CH_2_. However, for the simulations without the ER reactions, this alternative route goes backward (i.e., CH_2_ + O → CH_2_O → HCO + H) generating a cycle that is detrimental to methane formation. This is because, in this type of simulations, the O adatom is less likely to be hydrogenated, hence favoring the CH_2_ + O → CH_2_O reaction. Coming back to HCO dissociation to produce CH and O on Ni_O_ and Ni_Ce_, respectively, there are two possibilities for the reaction to proceed: CH hydrogenation to methane or O hydrogenation to water. Interestingly, water formation plays again an important role in the final activity and selectivity. We found that in the simulations including ER reactions, water is formed before methane, while the opposite behavior is observed in the simulations without ER reactions. This is supported by the difference in the diffusion steps of the CH_*x*_ species between the two different sites because if water is formed first, then the Ni_Ce_ site becomes free so that CH_*x*_ species can diffuse to this site as clearly seen in Figure 3a but not in Figure 3b. This is also supported by the fact that, in the simulations without ER reactions, there is a larger number of events for water formation at the Ni_O_ site (see Figure 3d). This is because methane is formed earlier at the Ni_O_ site (see Figure 3e), leaving the Ni_O_ site available so that O can diffuse to the Ni_O_ site, where it is further hydrogenated to water. For the scenario with ER reactions, Figure 3a shows that, after water formation, CH hydrogenates to CH_2_ that further hydrogenates to CH_3_ at Ni_O_. Then, a minor part of CH_3_ hydrogenates to CH_4_, which further desorbs, while most of CH_3_ diffuse to Ni_Ce_. Next, a fraction of CH_3_ hydrogenates to CH_4_ that further desorbs, while another fraction dissociates to CH_2_ that diffuses to the Ni_O_ site generating a cycle. At the end, this cycle is beneficial for the final methane production as methane is easily formed at the Ni_Ce_ site probably because of the larger adsorbate–adsorbate interactions at that site, which explains the highest CH_4_ site preference for this site in this type of simulations (see Figure 3e).

At this point, we have unveiled the mechanism for CO and methane formation. First, we have shown that CO is formed via the dissociative pathway of the RWGS reaction. Then, we have revealed that methane is formed as a combination of the dissociative pathway to produce CO followed by a mixture of the HCOO-mediated and dissociative pathways for the Sabatier reaction (i.e., CO_2_ → CO → HCO → CH → CH_2_ → CH_3_ → CH_4_). We have also shown that there is a clear synergy between the two different sites and that the ER reactions play an important role in the final mechanism, activity, and selectivity. In that sense, we have revealed that CO_2_ mainly dissociates at the Ni_O_ site, while CO desorbs similarly from both Ni_Ce_ and Ni_O_ sites. This is because water is formed before CO desorption takes place leaving a free site in which CO can diffuse, react, or desorb. The similar site preference for CO desorption for both types of simulations points to CO as a mere spectator for water formation. On the other hand, methane is formed on different sites depending on the type of simulation, which we attribute to differences in water formation. For the simulations that include the ER reactions, water is formed first leaving a free site in which CH_*x*_ species can diffuse and finally react to produce methane on the most favorable site. However, for the simulations without the ER reactions, water formation is hindered and methane is formed first in the less reactive Ni_O_ site. Finally, we suggest that adsorbate–adsorbate interactions between CH_*x*_ species and other species hinder water formation and lower the reactivity. This is even more evident for the simulations without ER reaction and explains the drastic change in the site preference of methane formation. Interestingly, our results are in quite good agreement with the ones reported by Onrubia-Calvo et al.^16^ In their work, these authors proposed different mechanisms for the CO_2_ methanation reaction and used the Langmuir–Hinshelwood–Hougen–Watson (LHHW) approach to derive a rate equation for their proposed mechanisms. From their simulations, they found different mechanisms that were able to predict, with different levels of confidence, their experimental results for the 8.5% Ni/CeO_2_ catalyst. They reported the best agreement for the formate mechanism (i.e., CO_2_ → HCOO → HCO → CH → CH_4_) followed by the formyl mechanism (i.e., CO_2_ → CO → HCO → CH → CH_4_). Their results are in quite good agreement with our mechanistic predictions in which we have also shown the importance of the HCO intermediate for the final CH formation and methane production. Regarding the HCOO intermediate, we have found that even if HCOO is formed during the simulations as a result of the endothermic nature of the HCOO → HCO + O reaction, the CO_2_ + H → HCOO → HCO + O total process goes in the backward direction being the CO_2_ dissociation the dominant reaction for CO formation that is further hydrogenated to HCO, thus being the formyl mechanism the dominant one. Regarding the results reported by Zhang et al.,^27^ we have also found a partial agreement with our results. In their work, they proposed the dominant reaction mechanism to be a mix of the carboxyl pathway for the CO formation followed by the CO-hydrogenation pathway for the Sabatier reaction (i.e., CO_2_ → COOH → CO → HCO → CH_2_O → CH_2_ → CH_3_ → CH_4_). In contrast, the present kMC simulations show that CO is mainly formed via the CO_2_ direct dissociation rather than the carboxyl pathway. Moreover, the HCO dissociation reaction is key for methane formation rather than the CH_2_O dissociation. Finally, comparing our results with previous kMC simulations for the Ni(111) surface,^53^ we have shown that some of the drawbacks that make Ni(111) surface not selective toward methane formation are not present in the Ni-supported model catalyst. Compared with Ni(111), these are the higher CO adsorption energy, the lower endothermicity of the HCO formation reaction (R10), and the smaller HCO dissociation energy barrier (R13) present for the supported Ni_4_ cluster that opens the HCO dissociation route as a possible source of CH_*x*_ species that are further hydrogenated. This fact directly points to a small Ni cluster supported on ceria as potential active and selective catalysts, in agreement with the main experimental findings. As a final remark, one may wonder whether a deeper insight into the mechanism can be reached from subsequent analysis. For instance, one can rely on the Campbell degree of rate control (DRC)^61^ to find out which are the elementary steps that most affect the selectivity toward methane. However, for a complex reaction with many elementary steps as the one considered in the present work, performing this type of analysis becomes rapidly unpractical as the number of additional kMC simulations becomes very large, and the computer time necessary to get converged results with respect to the simulation time becomes excessive. In any case, the present results provide compelling evidence that HCO formation and its further dissociation as well as water formation are the reactions controlling selectivity toward CH_4_, and it is unlikely that a DRC analysis will provide relevant additional information.

The coverage of the different species at the different sites with(out) the ER reactions for a temperature of 523 K deserves further comments. Results in Table 4 show that, as expected, H coverage is slightly higher for the simulations with the ER reactions. This is because once the ER reaction occurs, an H atom is released to the surface. Regarding the O coverage, for the simulations without the ER reactions, it is higher as in these simulations O is less likely to be hydrogenated. Interestingly, this difference is more pronounced for the Ni_Ce_ sites as O is generally formed at this site. Moreover, the high O coverage at Ni_Ce_ agrees with a higher site preference of water formation on the Ni_O_ site found in the simulations without the ER reactions, as the oxygen atoms at the Ni_Ce_ site require more time to be hydrogenated (see Figure 3d). For the simulations without the ER reactions, Table 4 also shows a larger CO coverage at Ni_O_ but a lower one on Ni_Ce_. This is directly related to water formation at Ni_Ce_; since water is produced before CO desorption, the less probable the H_2_O formation, the longer the CO stays at the Ni_O_ site with the concomitant increase of the CO coverage at that site. Similarly, as CO is blocked by the O atom, it cannot diffuse to the Ni_Ce_ site with the concomitant decrease of CO coverage at this site. Finally, for the simulations with the ER reactions, comparing the coverage at different temperatures (see Table S4), one can see that, as expected, the higher the temperature the lower the coverage. However, for the situation in which ER reactions are not considered, an increase of the temperature only decreases the coverage of H_2_ and H. Interestingly, the higher the temperature the higher the coverage of CO and O on Ni_O_ and Ni_Ce_, respectively, which is associated with a more difficult water formation at higher temperatures.

**Table 4:** Coverage of the Different Species Considered at the Different Sites for the Simulations Including the ER Reactions (W/ER) and without Them (Wo/ER) at *T* = 523 K, P(H_2_) = 0.528 bar, and P(CO_2_) = 0.132 bar^a^

*T* = 523 K	Ni_Ce_		Ni_O_		h_Ce_		h_O_	
species	W/ER	Wo/ER	W/ER	Wo/ER	W/ER	Wo/ER	W/ER	Wo/ER
CO	71.7 ± 0.1	67.7 ± 0.1	27.0 ± 0.1	30.2 ± 0.2				
O	0.7 ± 0.1	6.6 ± 0.2	0	0.5 ± 0.1				
OH	0.7 ± 0.1	0.5 ± 0.1	0	0				
H_2_					29.0 ± 0.3	29.3 ± 0.3	29.0 ± 0.3	29.3 ± 0.3
H					18.9 ± 0.2	17.3 ± 0.3	17.0 ± 0.2	15.6 ± 0.3
total	73.1	74.8	27.0	30.7	47.9	46.6	46.0	44.9

a Note that the present values are calculated as the mean value of 5 different kMC simulations for each working condition and including the standard deviation.

#### Analysis of Relevant Kinetic Parameters

**Figure 4:**
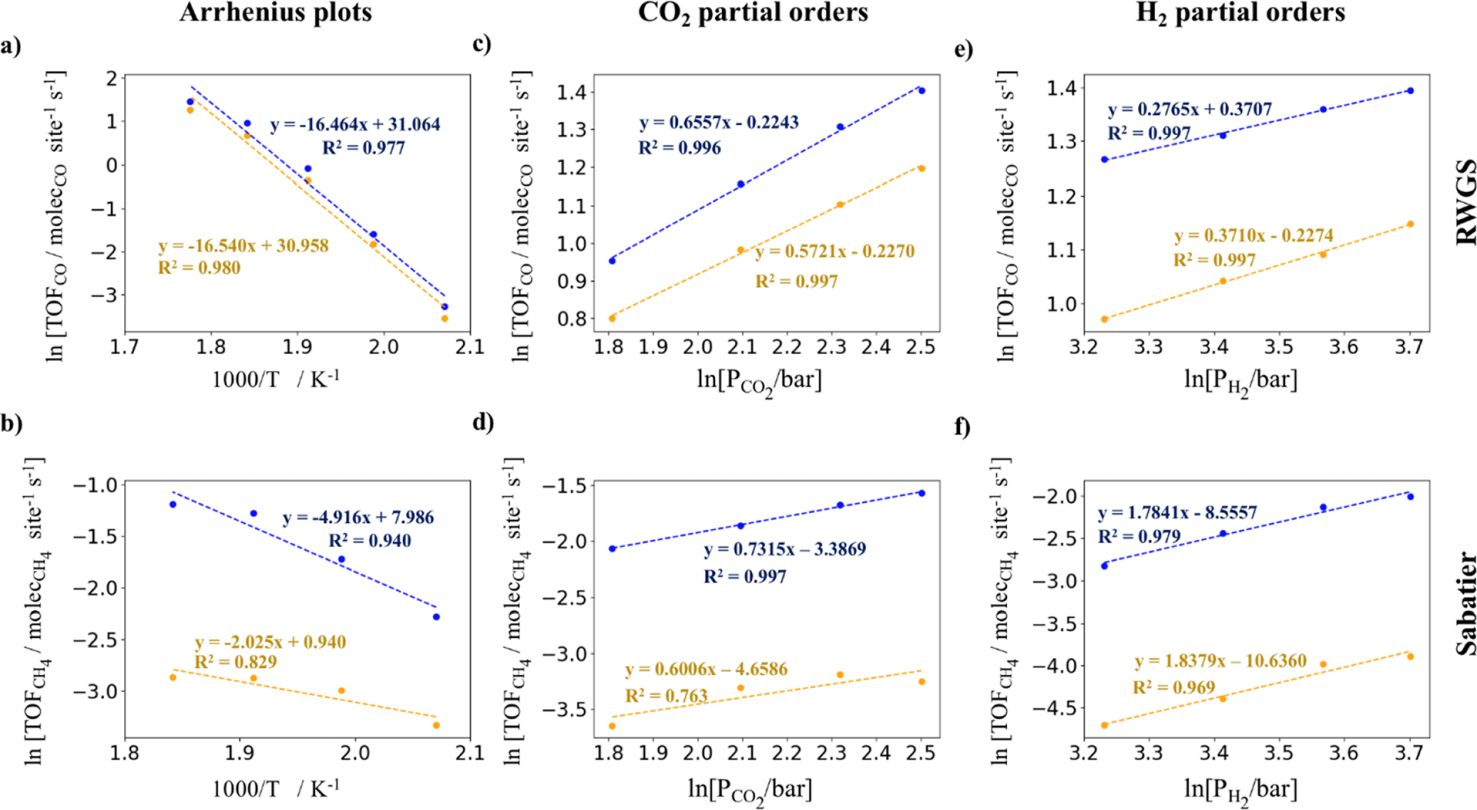
Arrhenius plot for the RWGS (a) and Sabatier (b) reactions, respectively, at P(H_2_) = 0.528 bar and P(CO_2_) = 0.132 bar. CO_2_ partial orders for the RWGS (c) and Sabatier (d) reactions, respectively, at fixed P(H_2_) = 0.54 bar. H_2_ partial orders for the RWGS (e) and Sabatier (f) reactions, respectively, at fixed P(CO_2_) = 0.135 bar. Blue and orange colors stand for the simulations with and without including the ER reactions, respectively.

We have obtained the apparent activation energy and partial orders of reaction with respect of both reactants (i.e., CO_2_ and H_2_) for the RWGS and Sabatier reactions as shown in Figure 4. First, for the sake of simplicity, we focus on the values for the simulations including the ER steps and compare them with the experimental values keeping in mind that the experimental system and the model system are similar but not identical. Next, we compare our values for the simulations with the ER reactions. From Figure 4a,b, we extract apparent activation energies of 136.9 and 40.9 kJ mol^–1^ for the RWGS and Sabatier reactions, respectively. Note that for the Sabatier reaction, we have only found an Arrhenius-like behavior for a temperature range between 483 and 543 K. Comparing our results with the experimental values reported by Zheng et al.^12^ of 92.7 and 131.6 kJ mol^–1^ for the RWGS and Sabatier reactions, respectively, we found higher values for the CO production but smaller values for methane formation. We assign the difference for the RWGS to the stronger CO adsorption on the small Ni cluster and the smaller apparent activation energy for methane production to the increased activity toward CH_4_ on the supported Ni_4_ cluster. This difference in the apparent activation energy for the Sabatier reaction contrasts with the values reported by Onrubia-Calvo et al.^16^ and Xie et al.^17^ of 53.9 kJ mol^–1^ and between 69.3 and 51.0 kJ mol^–1^, respectively, which nicely agree with our calculated value. We suggest that their low apparent activation energies are due to a high amount of interfacial oxygen vacancies, which enhances the methane formation in a comparable way as small Ni clusters do, as observed by the similar apparent activation energy of 40.9 kJ mol^–1^ that we have found. Thus, the present results point to small supported clusters as prominent candidates for CO_2_ hydrogenation to methane under mild conditions. Comparing the CO_2_ and H_2_ partial orders of reaction (cf. Figure 4c–f) to the experimental values reported by Zheng et al.,^12^ we found smaller CO_2_ partial orders of reaction (i.e., 0.66 and 0.73 for the RWGS and Sabatier reactions, respectively, vs 1.42 and 1.08), while larger H_2_ partial orders of reaction (i.e., 0.28 and 1.78 for the RWGS and Sabatier reactions, respectively, vs −0.04 and 1.07). In both cases, we attribute the differences to the larger nanoparticles likely to be present in the experimental system as well as to differences in the support structure. For the CO_2_ partial orders, our smaller values are directly associated with the limited number of available sites of our model, where an increase of the CO_2_ partial pressure only makes more probable CO_2_ to be adsorbed but does not change the number of CO_2_ adsorbed molecules (only one can be adsorbed at a time), which is not the case in the larger nanoparticles used in the experiments, thus producing a higher effect on the final reactivity. Regarding the H_2_ partial order of reaction, in our model, CO and CH_4_ formation is somehow linked to previous water formation. Therefore, the higher the H_2_ partial pressure, the more probable the water formation is, which increases the CO and CH_4_ formation. Nevertheless, in larger nanoparticles with more available free sites, CO and CH_4_ are not conditioned by water formation which explains the lower experimental values. Regarding the values reported by Onrubia-Calvo et al.,^16^ they found smaller CO_2_ and H_2_ partial orders of reaction (i.e., 0.06 and 0.29, respectively) for the Sabatier reaction than the ones we have found (i.e., 0.28 and 1.78, respectively). We suggest that the differences are due to the very large nanoparticles present in their experiments which produce a large number of oxygen vacancies and available Ni sites in which both CO_2_ and H_2_ can easily adsorb and react. According to this, an increase in the partial pressure of both reactants does not drastically change the final product formation.

Finally, we compare our values for the simulations with and without explicit consideration of the ER elementary steps. From Figure 4a,b, we extract apparent activation energies of 136.9 and 137.5 kJ mol^–1^ for the RWGS reaction and 40.9 and 16.8 kJ mol^–1^ for the Sabatier reaction, for the simulations with and without including the ER reactions, respectively. Regarding the values for the RWGS reaction, there are almost no differences, and we suggest that this is due to a similar CO desorption in both situations. For the Sabatier reaction, one can see a quite large difference, which suggests that the temperature has a higher effect on the ER reactions than on the surface reactions; hence, simulations with ER reactions are more affected by the temperature. For the CO_2_ partial orders, we have found slightly higher partial orders for the simulations with the ER reactions as shown in Figure 4c,d. As explained earlier, a higher CO_2_ pressure does not directly translate into a large number of CO_2_ molecules adsorbed on the surface. Nevertheless, the higher the CO_2_ partial pressure, the higher the probability of CO_2_ adsorption and reaction is. As the simulations with the ER reactions predict a higher activity, a higher probability of CO_2_ to be at the surface translates into a higher activity, which explains why CO_2_ partial orders are slightly higher when considering the ER reactions. Finally, regarding the H_2_ partial orders of reaction, we have found slightly lower partial orders for the simulations with the ER reactions (see Figure 4e,f), which suggest that the H_2_ partial pressure has a lower impact on the ER reactions rather than on the surface reactions. As a final remark, the overall results suggest that high methane selectivity requires working at higher H_2_ partial pressures.

## Conclusions

The mechanism of CO_2_ hydrogenation on a well-defined model of a Ni/CeO_2_ catalyst, consisting of a Ni_4_ cluster supported on the stoichiometric CeO_2_ (111) surface, has been thoroughly investigated by coupling DFT calculations with kMC simulations. The DFT calculations, carried out for an extensive reaction network, evidence that the adsorption energies and energy barriers of some important intermediates and elementary steps are significantly different from those corresponding to the extended Ni (111) surface. To a large extent, the origin of the different reactivity is due to metal–support interactions that change the Ni electronic structure along with differences in Ni atomic coordination that could be beneficial for catalytic purposes.

To further understand the mechanism of CO_2_ hydrogenation on the supported Ni cluster, we have performed several kMC simulations at different temperature and pressure conditions. From the kMC results, we are able to unravel the mechanism that governs the overall reactions as well as to gain insights about conditions defining the catalytic activity and the selectivity to methane. Moreover, we have thoroughly studied the effect of including some ER reactions on the final mechanisms, and their effect on the catalytic activity and selectivity. The kMC simulations unravel the existence of a synergic effect between the two different 3-fold Ni hollow sites present on the Ni_4_ cluster, in such a way that some reactions are dominant in one site while other reactions are mostly done on the other site. Remarkably, this effect is even more evident comparing the simulations with or without including the ER reactions.

The kMC simulations unveil the mechanism for CO and methane formation, CO being the most significant product at the highest temperatures, which agrees with the experimental observations for a catalytic system in which small Ni nanoparticles are likely to be present, while being methane the most important product at the lowest temperature, hence suggesting that working under mild conditions will result in a high methane selectivity. CO is produced via the dissociative pathway of the RWGS reaction while methane is formed as a combination of the dissociative pathway for the CO formation followed by a mixture of the HCOO-mediated and dissociative pathways for the final methane formation (i.e., CO_2_ → CO → HCO → CH → CH_2_ → CH_3_ → CH_4_). We have shown that CO is mainly formed on the Ni_O_ site following CO_2_ dissociation; then, once water is formed, some CO desorbs while some CO diffuses to the Ni_Ce_ site in which it can either desorb or being hydrogenated to HCO, which further dissociates to CH on the Ni_O_ site. Interestingly, for the simulations with ER reactions, water formation occurs before methane formation, and it helps methane formation to occur at the more reactive Ni_Ce_ site. However, for the simulations without the ER reactions, methane is formed on the less reactive Ni_O_ site before water formation, with a concomitant decrease in the activity and methane selectivity. Finally, the analysis of the partial order of reaction suggests that working at high H_2_ pressure leads to improved CH_4_ selectivity.

The present simulations clearly reveal that small Ni clusters supported on ceria are potential good candidates for high selective methane formation under mild conditions while being highly active and selective for CO at high temperatures. Moreover, considering the general belief of the increase of the catalytic activity due to interfacial oxygen vacancies of Ni/CeO_2_ systems and our conclusions suggesting that small Ni clusters supported on CeO_2_ are good catalysts for methane formation under mild conditions, one can suggest that Ni/CeO_2_ catalysts combining small Ni clusters with interfacial oxygen vacancies could be potential good catalysts for highly active low-temperature methane formation, which call for the design and investigation both at the theoretical and experimental level of Ni/CeO_2_ systems combining small Ni clusters with interfacial oxygen vacancies. Finally, the present results show that an accurate simulation of the CO_2_ hydrogenation on the Ni/CeO_2_ catalyst requires including ER steps, and it is likely that this will be the case for other supported catalysts as well.

## Data Availability

Optimized structures (i.e., VASP CONTCAR files) of all relevant structures and kMC inputs have been also made available on a public GitHub repository: https://github.com/plozanore/CO2_hydrogenation_on_well-defined_Ni-CeO2_model_catalyst

## Supporting Information Available

Information about details on the calculation of formation energies, cluster expansion Hamiltonians, and how to calculate lateral interactions, reaction energies, and energy barriers for the different considered process on the Ni4/CeO_2_ system, and coverages at different temperatures and event frequencies at the NiCe and NiO sites at the different studied temperatures (PDF)

## References

[ref1] Götz M.; Lefebvre J.; Mörs F.; Koch A. M.; Graf F.; Bajohr S.; Reimert R.; Kolb T. Renewable Power-to-Gas: A Technological and Economic Review. Renewable Energy 2016, 85, 1371–1390. 10.1016/j.renene.2015.07.066.

[ref2] Mazza A.; Bompard E.; Chicco G. Application of Power to Gas Technologies in Emerging Electrical Systems. Renew. Sust. Energy Rev. 2018, 92, 794–806. 10.1016/j.rser.2018.04.072.

[ref3] Ghaib K.; Ben-Fares F.-Z. Power-to-Methane: A State-of-the-art review. Renew. Sust. Ener. Rev. 2018, 81, 433–446. 10.1016/j.rser.2017.08.004.

[ref4] Aziz M. A. A.; Jalil A. A.; Triwahyono S.; Ahmad A. CO_2_ Methanation over Heterogeneous Catalysts: Recent Progress and Future Prospects. Green Chem. 2015, 17, 2647–2663. 10.1039/C5GC00119F.

[ref5] Wei W.; Jinlong G. Methanation of Carbon Dioxide: an Overview. Front. Chem. Sci. Eng. 2011, 5, 2–10. 10.1007/s11705-010-0528-3.

[ref6] Tada S.; Shimizu T.; Kameyama H.; Haneda T.; Kikuchi R. Ni/CeO_2_ Catalysts with High CO_2_ Methanation Activity and High CH_4_ Selectivity at Low Temperatures. Int. J. Hydrog. Energy 2012, 37, 5527–553. 10.1016/j.ijhydene.2011.12.122.

[ref7] Martin N. M.; Velin P.; Skoglundh M.; Bauer M.; Carlsson P. A. Catalytic Hydrogenation of CO_2_ to Methane over Supported Pd, Rh and Ni Catalysts. Catal. Sci. Technol. 2017, 7, 1086–1094. 10.1039/C6CY02536F.

[ref8] Cárdenas-Arenas A.; Quindimil A.; Davó-Quiñonero A.; Bailón-García E.; Lozano-Castelló D.; De-La-Torre U.; Pereda-Ayo B.; González-Marcos J. A.; González-Velasco J. R.; Bueno-López A. Isotopic and In Situ DRIFTS Study of the CO_2_ Metahanation Mechanism using Ni/CeO_2_ and Ni/Al_2_O_3_ Catalysts. Appl. Catal. B 2020, 265, 118538–118547. 10.1016/j.apcatb.2019.118538.

[ref9] Le T. A.; Kim M. S.; Lee S. H.; Kim T. W.; Park E. D. CO and CO_2_ Methanation over Supported Ni Catalysts. Catal. Today 2017, 293–294, 89–96. 10.1016/j.cattod.2016.12.036.

[ref10] Lin L.; Gerlak C. A.; Liu C.; Llorca J.; Yao S.; Rui N.; Zhang F.; Liu Z.; Zhang S.; Deng K.; Murray C. B.; Rodriguez J. A.; Senanayake S. D. Effect of Ni Particle Size on the Production of Renewable Methane from CO_2_ over Ni/CeO_2_. J. Energy Chem. 2021, 61, 602–611. 10.1016/j.jechem.2021.02.021.

[ref11] Winter L. R.; Gomez E.; Yan B.; Yao S.; Chen J. G. Tuning Ni-Catalyzed CO_2_ Hydrogenation Selectivity via Ni-ceria Support Interactions and Ni-Fe Bimetallic Formation. Appl. Catal. B 2018, 224, 442–450. 10.1016/j.apcatb.2017.10.036.

[ref12] Zheng H.; Liao W.; Ding J.; Xu F.; Jia A.; Huang W.; Zhang Z. Unveiling the Key Factors in Determining the Activity and Selectivity of CO_2_ Hydrogenation over Ni/CeO_2_ Catalysts. ACS Catal. 2022, 12, 15451–15462. 10.1021/acscatal.2c04437.

[ref13] Lin S.; Li Z.; Li M. Tailoring Metal-Support Interactions via Tuning CeO_2_ Particle Size for Enhancing CO_2_ Methanation Activity over Ni/CeO_2_ Catalysts. Fuel 2023, 333, 126369–126383. 10.1016/j.fuel.2022.126369.

[ref14] Riu N.; Zhang X.; Zhang F.; Liu Z.; Cao X.; Xie Z.; Zou R.; Senanayake S. D.; Yang Y.; Rodriguez J. A.; Liu C.-J. Highly Active Ni/CeO_2_ Catalyst for CO_2_ Methanation: Preparation and Characterization. Appl. Catal., B 2021, 282, 119581–119593. 10.1016/j.apcatb.2020.119581.

[ref15] Pu T.; Chen J.; Tu W.; Xu W.; Wachs I. E.; Zhu M.; Han Y.-F. Dependency of CO_2_ Methanation on the Strong Metal-Support Interaction for Supported Ni/CeO_2_ Catalysts. J. Catal. 2022, 413, 821–828. 10.1016/j.jcat.2022.07.038.

[ref16] Onrubia-Calvo J. A.; Quindimil A.; Dacó-Quiñonero A.; Bermejo-López A.; Bailón-García E.; Pereda-Ayo B.; Lozano-Castelló D.; González-Marcos J. A.; Bueno-López A.; González-Velasco J. R. Kinetics, Model Discrimination, and Parameters Estimation of CO_2_ Methanation on Highly Active Ni/CeO_2_ Catalyst. Ind. Eng. Chem. Res. 2022, 61, 10419–10435. 10.1021/acs.iecr.2c00164.

[ref17] Xie Y.; Chen J.; Wu X.; Wen J.; Zhao R.; Li Z.; Tian G.; Zhang Q.; Ning P.; Hao J. Frustrated Lewis Pairs Boosting Low-Temperature CO_2_ Methanation Performance over Ni/CeO_2_ Nanocatalysts. ACS. Catal. 2022, 12, 10587–10602. 10.1021/acscatal.2c02535.

[ref18] Barreau M.; Salusso D.; Li J.; Zhang J.; Borfecchia E.; Sobczak K.; Braglia L.; Gallet J.; Torelli P.; Guo H.; Lin S.; Zafeiratos S. Ionic Nickel Embedded in Ceria with High Specific CO_2_ Methanation Activity. Angew. Chem., Int. Ed. 2023, 62, e202302087 10.1002/anie.202302087. 37062698

[ref19] Liu Z.; Grinter D. C.; Lustemberg P. G.; Nguyen-Phan T.-D.; Zhou Y.; Luo Si; Waluyo I.; Crumlin E. J.; Stacchiola D. J.; Zhou J.; Carrasco J.; Busnengo H. F.; Ganguglia-Pirovano M. V.; Senanayake S. D.; Rodriguez J. A. Dry Reforming of Methane on a Highly-Active Ni-CeO_2_ Catalyst: Effects of Metal-Support Interactions on C–H Bond Breaking. Angew. Chem., Int. Ed. 2016, 55, 7455–7459. 10.1002/anie.201602489. 27144344

[ref20] Liu Z.; Lustemberg P.; Gutiérrez R. A.; Carey J. J.; Palomino R. M.; Vorokhta M.; Grinter D. C.; Ramírez P. J.; Matolín V.; Nolan M.; Ganduglia-Pirovano M. V.; Senanayake S. D.; Rodriguez J. A. In Situ Investigation of Methane Dry Reforming on Metal/Ceria(111) Surfaces: Metal–Support Interactions and C–H Bond Activation at Low Temperature. Angew, Chem. Int. Ed. 2017, 56, 13041–13046. 10.1002/anie.201707538. 28815842

[ref21] Lustemberg P. G.; Ramírez P. J.; Liu Z.; Gutiérrez R. A.; Grinter D. G.; Carrasco J.; Senanayake S. D.; Rodriguez J. A.; Ganduglia-Pirovano M. V. Room-Temperature Activation of Methane and Dry Reforming with CO_2_ on Ni-CeO_2_(111) Surfaces: Effect of Ce^3+^ Sites and Metal-Support Interactions on C-H Bond Cleavage. ACS Catal. 2016, 6 (12), 8184–8191. 10.1021/acscatal.6b02360.

[ref22] Lustemberg P. G.; Mao Z.; Salcedo A.; Irigoyen B.; Ganduglia-Pirovano M. V.; Campbell C. T. Nature of the Active Sites on Ni/CeO_2_ Catalysts for Methane Conversions. ACS Catal. 2021, 11, 10604–10613. 10.1021/acscatal.1c02154. 34484854 PMC8411779

[ref23] Lustemberg P. G.; Palomino R. M.; Gutiérrez R. A.; Grinter D. C.; Vorokhta M.; Liu Z.; Ramírez P. J.; Matolín V.; Ganduglia-Pirovano M. V.; Senanayake S. D.; Rodriguez J. A. Direct Conversion of Methane to Methanol on Ni-Ceria Surfaces: Metal-Support Interactions and Water-Enabled Catalytic Conversion by Site Blocking. J. Am. Chem. Soc. 2018, 140, 7681–7687. 10.1021/jacs.8b03809. 29804460

[ref24] Carrasco J.; Lopez-Duran D.; Liu Z.; Duchon T.; Evans J.; Senanayake S. D.; Crumlin E. J.; Matolín V.; Rodriguez J. A.; Ganduglia-Pirovano M. V. In Situ and Theoretical Studies for the Dissociation of Water on an Active Ni/CeO_2_ Catalyst: Importance of Strong Metal–Support Interactions for the Cleavage of O–H Bonds. Angew. Chem. Int. Ed 2015, 54, 3917–3921. 10.1002/anie.201410697. 25651288

[ref25] Salcedo A.; Lustemberg P. G.; Rui N.; Palomino R. M.; Liu Z.; Nemsak S.; Senanayake S. D.; Rodriguez J. A.; Ganduglia-Pirovano M. V.; Irigoyen B. Reaction Pathway for Coke-Free Methane Steam Reforming on a Ni/CeO_2_ Catalyst: Active Sites and the Role of Metal-Support Interactions. ACS Catal. 2021, 11, 8327–8337. 10.1021/acscatal.1c01604. 34306812 PMC8294006

[ref26] Lustemberg P. G.; Feria L.; Ganduglia-Pirovano M. V. Single Ni Sites Supported on CeO_2_(111) Reveal Cooperative Effects in the Water-Gas Shift Reaction. J. Phys. Chem. C 2019, 123, 7749–7757. 10.1021/acs.jpcc.8b06231.

[ref27] Zhang J.; Yang Y.; Liu J.; Xiong B. Mechanistic Understanding of CO_2_ Hydrogenation to Methane over Ni/CeO_2_. Appl. Surf. Sci. 2021, 558, 149866–149875. 10.1016/j.apsusc.2021.149866.

[ref28] Lozano-Reis P.; Prats H.; Sayós R.; Illas F. Limitations of Free Energy Diagrams to Predict the Catalytic Activity: The Reverse Water Gas Shift Reaction Catalyzed by Ni/TiC. J. Catal. 2023, 425, 203–211. 10.1016/j.jcat.2023.05.026.

[ref29] Zhai H.; Alexandrova A. N. Local Fluxionality of Surface-Deposited Cluster Catalysts: The Case of Pt_7_ on Al_2_O_3_. J. Phys. Chem. Lett. 2018, 9, 1696–1702. 10.1021/acs.jpclett.8b00379. 29551071

[ref30] Sun G.; Fuller J. T.; Alexandrova A. N.; Sautet P. Global Activity Search Uncovers Reaction Induced Concomitant Catalyst Restructuring for Alkane Dissociation on Model Pt Catalysts. ACS Catal. 2021, 11, 1877–1855. 10.1021/acscatal.0c05421.

[ref31] Zhang Z.; Zandkarimi B.; Alexandrova A. N. Ensembles of Metastable States Govern Heterogeneous Catalysis on Dynamic Interfaces. Acc. Chem. Res. 2020, 53, 447–458. 10.1021/acs.accounts.9b00531. 31977181

[ref32] Xia Z.; Yin Y.; Li J.; Xiao H. Single-atom Catalysis Enabled by High-energy Metastable Structures. Chem. Sci. 2023, 14, 2631–2639. 10.1039/D2SC06962H. 36908952 PMC9993862

[ref33] Mao Z.; Lustemberg P. G.; Rumptz J. R.; Gundiglia-Pirovano M. V.; Campbell C. T. Ni Nanoparticles on CeO_2_(111): Energetics, Electron Transfer, and Structure by Ni Adsorption Calorimetry, Spectroscopies, and Density Functional Theory. ACS Catal. 2020, 10, 5101–5114. 10.1021/acscatal.0c00333.

[ref34] Lustemberg P. G.; Senanayake S. D.; Rodriguez J. A.; Ganduglia-Pirovano M. V. Tuning Selectivity in the Direct Conversion of Methane to Methanol: Bimetallic Synergistic Effects on the Cleavage of C-H and O-H Bonds over NiCu/CeO_2_ Catalysts. J. Phys. Chem. Lett. 2022, 13, 5589–5596. 10.1021/acs.jpclett.2c00885. 35699247 PMC9234976

[ref35] Lozano-Reis P.; Sayós R.; Rodriguez J. A.; Illas F. Structural, Electronic, and Magnetic Properties of Ni Nanoparticles Supported on the TiC(001) Surface. Phys. Chem. Chem. Phys. 2020, 22, 26145–26154. 10.1039/D0CP04884D. 33185221

[ref36] Lozano-Reis P.; Prats H.; Sayós R.; Illas F. Assesing the Activity of Ni Clusters Supported on TiC(001) toward CO_2_ and H_2_ Dissociation. J. Phys. Chem. C 2021, 125, 12019–12027. 10.1021/acs.jpcc.1c03219.

[ref37] Kresse G.; Hafner J. Ab Initio Molecular Dynamics for Liquid Metals. Phys. Rev. B 1993, 47, 558–561. 10.1103/PhysRevB.47.558. 10004490

[ref38] Kresse G.; Furthmüller J. Efficient Iterative Schemes for Ab Initio Total-energy Calculations Using a Plane-wave Basis Set. Phys. Rev. B 1996, 54, 11169–11186. 10.1103/PhysRevB.54.11169. 9984901

[ref39] Kresse G.; Furthmüller J. Efficiency of Ab-initio Total Energy Calculations for Metals and Semiconductors Using a Plane-wave Basis Set. Comput. Mater. Sci. 1996, 6, 15–50. 10.1016/0927-0256(96)00008-0. 9984901

[ref40] Kresse G.; Joubert D. From Ultrasoft Pseudopotentials to the Projector Augmented-wave Method. Phys. Rev. B 1999, 59, 1758–1775. 10.1103/PhysRevB.59.1758.

[ref41] Perdew J. P.; Burke K.; Ernzerhof M. Generalized Gradient Approximation Made Simple. Phys. Rev. Lett. 1996, 77, 3865–3868. 10.1103/PhysRevLett.77.3865. 10062328

[ref42] Fabris S.; Vicario G.; Balducci G.; de Gironcoli S.; Baroni S. Electronic an Atomistic of Clean and Reduced Ceria Surfaces. J. Phys. Chem. B 2005, 109, 22860–22867. 10.1021/jp0511698. 16853978

[ref43] Cococcioni M.; de Gironcoli S. Linear Response Approach to the Calculation of the Effective Interaction Parameters in the LDA + U Method. Phys. Rev. B 2005, 71, 035105 10.1103/PhysRevB.71.035105.

[ref44] Grimme S.; Antony J.; Ehrlich S.; Krieg H. A Consistent and Accurate Ab Initio Parametrization of Density Functional Dispersion Correction (DFT-D) for the 94 Elements H-Pu. J. Chem. Phys. 2010, 132, 154104 10.1063/1.3382344. 20423165

[ref45] Bader R. F. W. A Quantum Theory of Molecular Structure and Structure and its Applications. Chem. Rev. 1991, 91 (5), 893–928. 10.1021/cr00005a013.

[ref46] Alvarez-Galvan C.; Lustemberg P. G.; Oropeza F. E.; Bachiller-Baeza B.; Ospina M. D.; Herranz M.; Cebollada J.; Collado L.; Campos-Martin J. M.; de la Peña-O’Shea V. A.; Alonso J. A.; Ganduglia-Pirovano M. V. ACS Appl. Mater. Interfaces 2022, 14, 50739–50750. 10.1021/acsami.2c11248. 36321841 PMC9673058

[ref47] Lustemberg P. G.; Zhang F.; Gutiérrez R. A.; Ramírez P. J.; Senanayake S. D.; Rodriguez J. A.; Ganduglia-Pirovano M. V. Breaking Simple Scaling Relations through Metal-Oxide interactions: Understanding Room-Temperature Activation of Methane on M/CeO_2_ (M = Pt, Ni or Co) Interfaces. J. Phys. Chem. Lett. 2020, 11, 9131–9137. 10.1021/acs.jpclett.0c02109. 33052684

[ref48] Monkhorst H. J.; Pack J. D. Special Points for Brillouin-zone Integrations. Phys. Rev. B 1976, 13, 5188–5192. 10.1103/PhysRevB.13.5188.

[ref49] Jónsson H.; Mills G.; Jacobsen K. W. Nudged Elastic Band Method for Finding Minimum Energy Paths of Transitions. In Classical and Quantum Dynamics in Condensed Phase Simulations; World scientific: Lerici, Villa Marigola, 1998, pp 385–404.

[ref50] Henkelman G.; Uberuaga B. P.; Jónsson H. A Climbing Image Nudged Elastic Band Method for Finding Saddle Points and Minimum Energy Paths. J. Chem. Phys. 2000, 113, 9901–9904. 10.1063/1.1329672.

[ref51] Smidstrup S.; Pedersen A.; Stokbro K.; Jónsson H. Improved Initial guess for Minimum Energy Path Calculations. J. Chem. Phys. 2014, 140, 214106 10.1063/1.4878664. 24907989

[ref52] Hjorth Larsen A.; Jo̷rgen Mortensen J.; Blomqvist J.; Castelli I. E.; Christensen R.; Dułak M.; Friis J.; Groves M. N.; Hammer B.; Hargus C.; Hermes E. D.; Jennings P. C.; Bjerre Jensen P.; Kermode J.; Kitchin J. R.; Leonhard Kolsbjerg E.; Kubal J.; Kaasbjerg K.; Lysgaard S.; Bergmann Maronsson J.; Maxson T.; Olsen R.; Pastewka L.; Peterson A.; Rostgaard C.; Schio̷tz J.; Schütt O.; Strange M.; Thygesen K. S.; Vegge T.; Vilhelmsen L.; Walter M.; Zeng Z.; Jacobsen K. W. The Atomic Simulation Environment—a Python Library for Working with Atoms. J. Phys.: Condens. Matter. 2017, 29, 273002 10.1088/1361-648X/aa680e. 28323250

[ref53] Lozano-Reis P.; Prats H.; Gamallo P.; Illas F.; Sayós R. Multiscale Study of the Mechanism of Catalytic CO_2_ Hydrogenation: Role of the Ni(111) Facets. ACS Catal. 2020, 10, 8077–8089. 10.1021/acscatal.0c01599.

[ref54] Stamatakis M.; Vlachos D. G. A Graph-theoretical Kinetic Monte Carlo Framework for on-lattice Chemical Kinetics. J. Chem. Phys. 2011, 134, 214115 10.1063/1.3596751. 21663352

[ref55] Stamatakis M.; Vlachos D. G. Unraveling the Complexity of Catalytic Reactions Via kinetic Monte Carlo Simulation: Current Status and Frontiers. ACS Catal. 2012, 2, 2648–2663. 10.1021/cs3005709.

[ref56] Nielsen J.; d’Avezac M.; Hetherington J.; Stamatakis M. Parallel kinetic Monte Carlo Simulation Framework Incorporating Accurate Models of Adsorbate Lateral Interactions. J. Chem. Phys. 2013, 139, 224706 10.1063/1.4840395. 24329081

[ref57] Prats H.; Posada-Pérez S.; Rodriguez J. A.; Sayós R.; Illas F. Kinetic Monte Carlo Simulations Unveil Synergic Effects at Work on Bifunctional Catalysts. ACS Catal. 2019, 9, 9117–9126. 10.1021/acscatal.9b02813.

[ref58] Prats H.; Álvarez L.; Illas F.; Sayós R. Kinetic Monte Carlo Simulations of the Water Gas Shift reaction on Cu(1 1 1) From Density Functional Theory Based Calculations. J. Catal. 2016, 333, 217–226. 10.1016/j.jcat.2015.10.029.

[ref59] Piccinin S.; Stamatakis M. CO Oxidation on Pd(111): a First-principles-based Kinetic Monte Carlo Study. ACS Catal. 2014, 4, 2143–2152. 10.1021/cs500377j.

[ref60] Yang L.; Karim A.; Muckerman J. T. Density Functional Kinetic Monte Carlo Simulation of Water–gas Shift Reaction on Cu/ZnO. J. Phys. Chem. C 2013, 117, 3414–3425. 10.1021/jp3114286.

[ref61] Campbell C. T. The Degree of Rate Control: a Powerful Tool for Catalysis Research. ACS Catal. 2017, 7, 2770–2779. 10.1021/acscatal.7b00115.

