# Supplementary material for: Comprehensive Density Functional and Kinetic Monte Carlo Study of CO2 Hydrogenation on a Well-Defined Ni/CeO2 Model Catalyst: Role of Eley–Rideal Reactions
Source: ACS Catal. 2024 Jan 30;14(4):2284–99. doi: 10.1021/acscatal.3c05336 (PMC10877572; doi:10.1021/acscatal.3c05336)
Supplement: Supplementary file 1 — cs3c05336_si_001.pdf [file cs3c05336_si_001.pdf]

Supporting Information

Comprehensive Density Functional and Kinetic Monte Carlo Study of CO_2 Hydrogenation on a Well-Defined Ni/CeO_2 Model Catalyst: Role of Eley-Rideal Reactions

Pablo Lozano-Reis, Pablo Gamallo*, Ramón Sayós, Francesc Illas*

Departament de Ciència de Materials i Química Física & Institut de Química Teòrica i Computacional (IQTCUB), Universitat de Barcelona, C. Martí i Franquès 1, 08028 Barcelona, Spain

**corresponding authors: gamallo@ub.edu, p.lozano@ub.edu*

S1. Formation energies calculation.

The reference set for the formation energy values is {slab, $H_{2(g)}$, $H_2O_{(g)}$ and $CO_{(g)}$} where slab stands for the energy of the Ni_4/CeO_2 surface while $H_{2(g)}$, $H_2O_{(g)}$ and $CO_{(g)}$ are the DFT energies of the gas-phase molecules. Therefore, the formation energy on the *i* adsorbate is calculated as:

$$E_i^f = U_{i-slab} - U_{slab} - \sum_j (n_j R_j) \quad (1)$$

Where U_{slab} is the DFT energy of the pristine slab, U_{i-slab} is the DFT energy of the adsorbate *i* on the slab, n_j is the number of atoms *j* in species *i*, and R_j is the reference energy of the atom *j*, defined in our reference set as:

$$R_H = 0.5\left(U_{H_{2(g)}}\right) \quad (2)$$

$$R_O = U_{H_2O_{(g)}} - 2R_H \quad (3)$$

$$R_C = U_{CO_{(g)}} - R_O \quad (4)$$

where $U_{i_{(g)}}$ is the DFT energy for the *i* gas-phase species. Following the above definition, the formation energy (FE) of the different species at the different sites for the Ni_4/CeO_2 system is calculated and summarized in Table S1.

Table S1. Formation Energies Including the ZPE Term for All the Different Species at the Different Considered Sites on the Ni_4/CeO_2 system. The * and ** Symbols Stand for Monodentate (One Site) or Bidentate (Two Sites) Adsorbed Species, Respectively.

Species	FE /eV	Species	FE / eV	Species	FE / eV	Species	FE / eV
$H_{2,hCehO}^{**}$	-0.63	CO_{NiO}^{*}	-2.33	$CH_2OH_{NiCe}^{*}$	-2.43	$H_2O_{NiO}^{*}$	-0.75
$CO_{2,NiO-NiCe}^{**}$	-2.35	CO_{NiCe}^{*}	-2.47	$CH_{3,NiO}^{*}$	-3.15	$H_2O_{NiCe}^{*}$	-0.73
$CO_{2,NiCe-NiO}^{**}$	-2.30	C_{NiO}^{*}	-1.36	$CH_{3,NiCe}^{*}$	-3.22	H_{hO}^{*}	-0.74
$COOH_{NiO-NiCe}^{**}$	-2.36	C_{NiCe}^{*}	-1.43	$CH_3O_{NiO}^{*}$	-2.83	H_{hCe}^{*}	-0.81
$COOH_{NiCe-NiO}^{**}$	-2.44	CH_{NiO}^{*}	-1.91	$CH_3O_{NiCe}^{*}$	-2.92	$HCOH_{NiO}^{*}$	-1.96
$HCOO_{NiO}^{*}$	-2.70	CH_{NiCe}^{*}	-2.09	$CH_{4,NiO}^{*}$	-2.62	$HCOH_{NiCe}^{*}$	-1.94
$HCOO_{NiCe}^{*}$	-2.73	$CH_{2,NiO}^{*}$	-2.49	$CH_{4,NiCe}^{*}$	-2.62	$CH_3OH_{NiO}^{*}$	-2.13
HCO_{NiO}^{*}	-2.26	$CH_{2,NiCe}^{*}$	-2.62	O_{NiO}^{*}	-1.01	$CH_3OH_{NiCe}^{*}$	-2.11
HCO_{NiCe}^{*}	-2.22	$CH_2O_{NiO}^{*}$	-2.36	O_{NiCe}^{*}	-1.23		
COH_{NiO}^{*}	-1.90	$CH_2O_{NiCe}^{*}$	-2.39	OH_{NiO}^{*}	-1.40		
COH_{NiCe}^{*}	-1.99	$CH_2OH_{NiO}^{*}$	-2.43	OH_{NiCe}^{*}	-1.49		

S2. Cluster expansion Hamiltonians and how to approximate lateral interactions.

To correctly define the energetics of the system of interest, at each surface configuration, cluster expansion Hamiltonians are used which account for the interactions between the different adsorbed species. With this definition the energy of a specific configuration is calculated as a sum of the different clusters that can represent one-body to multi-body terms. Hence, a cluster could be a single absorbed species or a group of neighboring species interacting among them. For instance, the cluster representing a two-body term interaction (normally called lateral interactions) between two species A-A is calculated as:

$$CE(A - A) = E^f_{A-A} - 2E^f_A \quad (5)$$

where E^f_A and E^f_{A-A} stands for the formation energy of the A species alone and the formation energy of the two neighboring species, respectively.

Finally, the energetics of a specific i lattice configuration is expressed as the sum of cluster energies:

$$E(i) = \sum_{k=1}^{N_C} n_k(i) \cdot CE_k \quad (6)$$

where $E(i)$ is the total energy of the system (*i.e.,* the energy of the i lattice configuration), N_C is the total number of clusters included in the model, CE_k is the cluster energy of cluster k and $n_k(i)$ is the number of times that a pattern for k-cluster appears. Note that the formation energy of a single adsorbed species is equivalent to the cluster energy of the adsorbed species. Table S2 summarize the different two-body terms (or lateral interactions) used in the cluster expansion.

Table S2. Pairwise Lateral Interactions (LI), Including the ZPE term, Between the Most Relevant Species Involved in the CO_2 Hydrogenation Reaction on the Ni_4/CeO_2 system. These Values Correspond to the Two-body Terms Used in the Cluster Expansion. The "-" Symbol is Used to Distinguish the Two Different Species in the kMC Simulation. The * and ** Symbols Stand for Monodentate (One Site) or Bidentate (Two Sites) Adsorbed Species, Respectively.

Species	LI / eV	Species	LI / eV	Species	LI / eV
H^*_{hCe}- H^*_{hO}	0.21	$CH_2O^*_{NiCe}$ - H^*_{hCe}	0.91	$HCOO^*_{NiO}$- H^*_{hCe}	0.38
CO^*_{NiCe} - O^*_{NiO}	0.48	$CH_2O^*_{NiO}$ - H^*_{hO}	0.84	$HCOO^*_{NiCe}$- H^*_{hCe}	0.70
CO^*_{NiO} - O^*_{NiCe}	0.38	$CH_2O^*_{NiCe}$- H^*_{hO}	0.35	$HCOO^*_{NiCe}$- H^*_{hO}	0.22
$CO^{**}_{2,NiO-NiCe}$ - H^*_{hCe}	0.53	$CH_2O^*_{NiO}$- H^*_{hCe}	0.30	$CO^{**}_{2,NiCe-NiO}$- H^*_{hCe}	0.96
$CO^{**}_{2,NiCe-NiO}$ - H^*_{hO}	0.38	$CH^*_{2,NiCe}$ - O^*_{NiO}	1.07	$CO^{**}_{2,NiO-NiCe}$- H^*_{hO}	0.73
CO^*_{NiCe} - OH^*_{NiO}	0.24	$CH^*_{2,NiO}$ - O^*_{NiCe}	0.94	CO^*_{NiO}- H^*_{hO}	0.74
CO^*_{NiO} - OH^*_{NiCe}	0.10	CH^*_{NiO}- OH^*_{NiCe}	0.55	CO^*_{NiCe}- H^*_{hCe}	0.55
HCO^*_{NiO} - O^*_{NiCe}	0.99	CH^*_{NiCe} - OH^*_{NiO}	0.64	C^*_{NiO}- H^*_{hO}	0.78
HCO^*_{NiCe} - O^*_{NiO}	0.73	C^*_{NiCe}- OH^*_{NiO}	0.57	C^*_{NiCe}- H^*_{hCe}	0.77
C^*_{NiO} - O^*_{NiCe}	1.07	C^*_{NiO} - OH^*_{NiCe}	0.58	CH^*_{NiCe}- H^*_{hCe}	0.66
C^*_{NiCe} - O^*_{NiO}	0.99	$CH^*_{2,NiCe}$ - OH^*_{NiO}	0.66	CH^*_{NiO}- H^*_{hO}	0.57
C^*_{NiO} - H^*_{hCe}	0.23	$CH^*_{2,NiO}$ - OH^*_{NiCe}	0.64	$CH^*_{2,NiO}$- H^*_{hO}	0.72
C^*_{NiCe} - H^*_{hO}	0.26	$CH_3O^*_{NiCe}$- H^*_{hO}	0.44	$CH^*_{2,NiCe}$- H^*_{hCe}	0.74
CH^*_{NiO} - H^*_{hCe}	0.21	$CH_3O^*_{NiO}$- H^*_{hCe}	0.42	COH^*_{NiCe}- H^*_{hCe}	0.71
CH^*_{NiCe}- H^*_{hO}	0.25	$CH^*_{3,NiCe}$ - OH^*_{NiO}	0.80	COH^*_{NiO}- H^*_{hO}	0.68
$CH^*_{2,NiO}$ - H^*_{hCe}	0.21	$CH^*_{3,NiO}$ - OH^*_{NiCe}	0.76	$COOH^{**}_{NiO-NiCe}$- H^*_{hO}	0.77
$CH^*_{2,NiCe}$ - H^*_{hO}	0.24	$CH^*_{3,NiCe}$ - O^*_{NiO}	0.91	$COOH^{**}_{NiCe-NiO}$- H^*_{hCe}	0.83
$CH^*_{3,NiCe}$ - H^*_{hCe}	0.88	$CH^*_{3,NiO}$ - O^*_{NiCe}	0.97	$COOH^{**}_{NiO-NiCe}$- H^*_{hCe}	0.41
$CH^*_{3,NiO}$ - H^*_{hO}	0.84	COH^*_{NiCe}- H^*_{hO}	0.18	$COOH^{**}_{NiCe-NiO}$- H^*_{hO}	0.48
O^*_{NiO} - H^*_{hCe}	0.35	COH^*_{NiO}- H^*_{hCe}	0.17	$HCOH^*_{NiCe}$- H^*_{hCe}	0.74
O^*_{NiCe} - H^*_{hO}	0.43	COH^*_{NiCe}- O^*_{NiO}	0.86	$HCOH^*_{NiO}$- H^*_{hO}	0.72
OH^*_{NiO}- H^*_{hCe}	0.44	COH^*_{NiO}- O^*_{NiCe}	0.96	$CH^*_{3,NiCe}$ - H^*_{hO}	0.56

OH^*_{NiCe}- H^*_{hO}	0.48	$CH_2OH^*_{NiCe}$- H^*_{hO}	0.57	$CH^*_{3,NiO}$ - H^*_{hCe}	0.49
CO^*_{NiO}- H^*_{hCe}	0.08	$CH_2OH^*_{NiO}$- H^*_{hCe}	0.54	$CH_3O^*_{NiCe}$- H^*_{hCe}	0.92
CO^*_{NiCe}- H^*_{hO}	0.39	$HCOH^*_{NiCe}$- H^*_{hO}	0.46	$CH_3O^*_{NiO}$- H^*_{hO}	0.89
CH^*_{NiCe}- O^*_{NiO}	1.18	$HCOH^*_{NiO}$- H^*_{hCe}	0.55	$CH_2OH^*_{NiCe}$- H^*_{hCe}	0.88
CH^*_{NiO} - O^*_{NiCe}	1.22	O^*_{NiO}- H^*_{hO}	0.76	$CH_2OH^*_{NiO}$- H^*_{hO}	0.74
HCO^*_{NiO}- H^*_{hCe}	0.29	O^*_{NiCe}- H^*_{hCe}	0.84	HCO^*_{NiO} - OH^*_{NiCe}	1.03
HCO^*_{NiCe} - H^*_{hO}	0.28	OH^*_{NiO}- H^*_{hO}	0.89	HCO^*_{NiCe} - OH^*_{NiO}	0.89
HCO^*_{NiO} - H^*_{hO}	0.77	OH^*_{NiCe}- H^*_{hCe}	0.93	H^*_{hCe}- H^*_{hCe}	0.88
HCO^*_{NiCe}- H^*_{hCe}	0.75	$HCOO^*_{NiO}$- H^*_{hO}	0.67	H^*_{hO}- H^*_{hO}	0.91

Table S3. Reaction Energies ($\Delta E_{0,r}$) and Forward and Reverse Energy Barriers ($\Delta E^{\neq}_{0,f}$, $\Delta E^{\neq}_{0,r}$), Including the ZPE Term, for the Different Elementary Reactions Considered for the CO_2 Hydrogenation Reaction for the Ni_4/CeO_2 System with Corresponding Values for the Reaction at the Ni(111) Surface[1] Included for Comparison. For Reactions in which Two Possible Hydrogen Attacks are Considered the *f* and *n* Subscript Stands for the H Atom Being at the Site that is Far or Near the Attacking Species, Respectively. For Instance, *f* Stands for Situations in which H and the Other Species are at h_O/Ni_{Ce} or h_{Ce}/Ni_O, Respectively, and *n* Stands for Situations in which H and the Other Species are at h_{Ce}/Ni_{Ce} or h_O/Ni_O, Respectively. The * and ** Symbols Stand for Monodentate (One Site) or Bidentate (Two Sites) Adsorbed Species, Respectively.

ID	Reaction	$\Delta E_{0,r}$ / eV		$\Delta E^{\neq}_{0,f}$ / eV		$\Delta E^{\neq}_{0,r}$ / eV	
		This Work	Ref 1	This Work	Ref 1	This Work	Ref 1
$R1_{NiO}$	$CO_{2,(g)} + *_{NiO} \rightleftharpoons CO^{**}_{2,NiO-NiCe}$	-1.51	-0.16	0.00	0.00	1.46	0.16
$R1_{NiCe}$	$CO_{2,(g)} + *_{NiCe} \rightleftharpoons CO^{**}_{2,NiCe-NiO}$	-1.46	-0.16	0.00	0.00	1.46	0.16
R2	$H_{2,(g)} + *_{hCe} + *_{hO} \rightleftharpoons H^{**}_{2,hCe-hO}$	-0.63	0.00	0.00	0.00	0.63	0.00
R3	$H^{**}_{2,hCe-hO} \rightleftharpoons H^{*}_{hCe} + H^{*}_{hO}$	-0.70	-0.33	0.00	0.26	0.70	0.59
$R4_{NiO}$	$CO^{**}_{2,NiO-NiCe} + *_{NiCe} \rightleftharpoons CO^{*}_{NiO} + O^{*}_{NiCe}$	-0.82	-0.57	0.78	0.86	1.60	1.43
$R4_{NiCe}$	$CO^{**}_{2,NiCe-NiO} + *_{NiO} \rightleftharpoons CO^{*}_{NiCe} + O^{*}_{NiO}$	-0.70	-0.57	0.77	0.86	1.47	1.43
$R5_{NiO}$	$CO^{**}_{2,NiO} + H^{*}_{hCe} \rightleftharpoons HCOO^{*}_{NiO} + *_{hCe}$	-0.07	0.04	0.60	1.05	0.45	1.01
$R5_{NiCe}$	$CO^{**}_{2,NiCe-NiO} + H^{*}_{hO} \rightleftharpoons HCOO^{*}_{NiCe} + *_{hO}$	-0.07	0.04	0.39	1.05	0.45	1.01
$R6_{NiO}$	$CO^{**}_{2,NiO-NiCe} + H^{*}_{hCe} \rightleftharpoons COOH^{**}_{NiO-NiCe} + *_{hCe}$	0.26	0.49	1.16	1.33	0.90	0.84
$R6_{NiCe}$	$CO^{**}_{2,NiCe-NiO} + H^{*}_{hO} \rightleftharpoons COOH^{**}_{NiCe-NiO} + *_{hO}$	0.22	0.49	1.20	1.33	0.8	0.84
$R7_{NiO}$	$COOH^{**}_{NiO-NiCe} + *_{NiCe} \rightleftharpoons COH^{*}_{NiCe} + O^{*}_{NiO}$	0.23	0.07	1.33	1.28	1.10	1.21
$R7_{NiCe}$	$COOH^{**}_{NiCe-NiO} + *_{NiO} \rightleftharpoons COH^{*}_{NiO} + O^{*}_{NiCe}$	0.28	0.07	1.35	1.28	1.07	1.21
$R8_{NiO}$	$COOH^{**}_{NiO-NiCe} + *_{NiCe} \rightleftharpoons CO^{*}_{NiO} + OH^{*}_{NiCe}$	-1.36	-1.02	0.49	0.49	1.85	1.51
$R8_{NiCe}$	$COOH^{**}_{NiCe-NiO} + *_{NiO} \rightleftharpoons CO^{*}_{NiCe} + OH^{*}_{NiO}$	-1.20	-1.02	0.64	0.49	1.84	1.51
$R9_{NiO}$	$HCOO^{*}_{NiO} + *_{NiCe} \rightleftharpoons HCO^{*}_{NiO} + O^{*}_{NiCe}$	0.20	0.85	0.59	1.39	0.39	0.54
$R9_{NiCe}$	$HCOO^{*}_{NiCe} + *_{NiO} \rightleftharpoons HCO^{*}_{NiCe} + O^{*}_{NiO}$	0.23	0.85	0.65	1.39	0.42	0.54
$R10_{NiO}$	$CO^{*}_{NiO} + H^{*}_{hCe} \rightleftharpoons HCO^{*}_{NiO} + *_{hCe}$	0.80	1.21	1.12	1.42	0.32	0.21
$R10_{NiCe}$	$CO^{*}_{NiCe} + H^{*}_{hO} \rightleftharpoons HCO^{*}_{NiCe} + *_{hO}$	0.60	1.21	0.90	1.42	0.30	0.21
$R11_{NiO}$	$CO^{*}_{NiO} + H^{*}_{hCe} \rightleftharpoons COH^{*}_{NiO} + *_{hCe}$	1.16	1.07	2.18	2.28	1.02	1.21
$R11_{NiCe}$	$CO^{*}_{NiCe} + H^{*}_{hO} \rightleftharpoons COH^{*}_{NiCe} + *_{hO}$	0.83	1.07	1.98	2.28	1.15	1.21
$R12_{NiO}$	$CO^{*}_{NiO} + *_{NiCe} \rightleftharpoons C^{*}_{NiO} + O^{*}_{NiCe}$	0.82	1.84	1.24	2.98	0.42	1.15
$R12_{NiCe}$	$CO^{*}_{NiCe} + *_{NiO} \rightleftharpoons C^{*}_{NiCe} + O^{*}_{NiO}$	1.03	1.84	1.52	2.98	0.49	1.15
$R13_{NiO}$	$HCO^{*}_{NiO} + *_{NiCe} \rightleftharpoons CH^{*}_{NiCe} + O^{*}_{NiO}$	0.34	-0.07	0.75	1.10	0.41	1.17
$R13_{NiCe}$	$HCO^{*}_{NiCe} + *_{NiO} \rightleftharpoons CH^{*}_{NiO} + O^{*}_{NiCe}$	0.31	-0.07	0.64	1.10	0.33	1.17
$R14_{NiO}$	$COH^{*}_{NiO} + *_{NiCe} \rightleftharpoons C^{*}_{NiO} + OH^{*}_{NiCe}$	-0.37	0.67	1.09	1.81	1.46	1.14

$R14_{NiCe}$	$COH^*_{NiCe} + *_{NiO} \rightleftharpoons C^*_{NiCe} + OH^*_{NiO}$	-0.28	0.67	1.07	1.81	1.35	1.14
$R15_{NiO,f}$	$C^*_{NiO} + H^*_{hCe} \rightleftharpoons CH^*_{NiO} + *_{hCe}$	0.02	-0.63	0.58	0.64	0.56	1.27
$R15_{NiCe,f}$	$C^*_{NiCe} + H^*_{hO} \rightleftharpoons CH^*_{NiCe} + *_{hO}$	-0.18	-0.63	0.48	0.64	0.66	1.27
$R15_{NiO,n}$	$C^*_{NiO} + H^*_{hO} \rightleftharpoons CH^*_{NiO} + *_{hO}$	-0.59	-0.63	0.03	0.64	0.62	1.27
$R15_{NiCe,n}$	$C^*_{NiCe} + H^*_{hCe} \rightleftharpoons CH^*_{NiCe} + *_{hCe}$	-0.62	-0.63	0.12	0.64	0.75	1.27
$R16_{NiO,f}$	$CH^*_{NiO} + H^*_{hCe} \rightleftharpoons CH^*_{2,NiO} + *_{hCe}$	0.01	0.30	0.37	0.64	0.36	0.34
$R16_{NiCe,f}$	$CH^*_{NiCe} + H^*_{hO} \rightleftharpoons CH^*_{2,NiCe} + *_{hO}$	-0.04	0.30	0.33	0.64	0.37	0.34
$R16_{NiO,n}$	$CH^*_{NiO} + H^*_{hO} \rightleftharpoons CH^*_{2,NiO} + *_{hO}$	-0.41	0.30	0.20	0.64	0.43	0.34
$R16_{NiCe,n}$	$CH^*_{NiCe} + H^*_{hCe} \rightleftharpoons CH^*_{2,NiCe} + *_{hCe}$	-0.38	0.30	0.03	0.63	0.41	0.34
$R17_{NiO,f}$	$CH^*_{2,NiO} + H^*_{hCe} \rightleftharpoons CH^*_{3,NiO} + *_{hCe}$	-0.07	-0.11	0.44	0.57	0.51	0.68
$R17_{NiCe,f}$	$CH^*_{2,NiCe} + H^*_{hO} \rightleftharpoons CH^*_{3,NiCe} + *_{hO}$	-0.10	-0.11	0.44	0.57	0.54	0.68
$R17_{NiO,n}$	$CH^*_{2,NiO} + H^*_{hO} \rightleftharpoons CH^*_{3,NiO} + *_{hO}$	-0.64	-0.11	0.03	0.57	0.67	0.68
$R17_{NiCe,n}$	$CH^*_{2,NiCe} + H^*_{hCe} \rightleftharpoons CH^*_{3,NiCe} + *_{hCe}$	-0.53	-0.11	0.09	0.57	0.62	0.68
$R18_{NiO}$	$COH^*_{NiO} + H^*_{hCe} \rightleftharpoons HCOH^*_{NiO} + *_{hCe}$	0.56	0.67	1.21	0.82	0.65	0.14
$R18_{NiCe}$	$COH^*_{NiCe} + H^*_{hO} \rightleftharpoons HCOH^*_{NiCe} + *_{hO}$	0.60	0.67	1.26	0.82	0.66	0.14
$R19_{NiO}$	$HCO^*_{NiO} + H^*_{hO} \rightleftharpoons HCOH^*_{NiO} + *_{hO}$	0.26	0.40	0.88	0.93	0.62	0.53
$R19_{NiCe}$	$HCO^*_{NiCe} + H^*_{hCe} \rightleftharpoons HCOH^*_{NiCe} + *_{hCe}$	0.33	0.40	0.89	0.93	0.55	0.53
$R20_{NiO}$	$HCOH^*_{NiO} + *_{NiCe} \rightleftharpoons CH^*_{NiCe} + OH^*_{NiO}$	-0.23	-0.48	0.24	0.72	0.47	1.19
$R20_{NiCe}$	$HCOH^*_{NiCe} + *_{NiO} \rightleftharpoons CH^*_{NiO} + OH^*_{NiCe}$	-0.28	-0.48	0.36	0.72	0.63	1.19
$R21_{NiO}$	$HCOH^*_{NiO} + H^*_{hCe} \rightleftharpoons CH_2OH^*_{NiO} + *_{hCe}$	-0.20	0.22	0.73	0.63	0.93	0.41
$R21_{NiCe}$	$HCOH^*_{NiCe} + H^*_{hO} \rightleftharpoons CH_2OH^*_{NiCe} + *_{hO}$	-0.20	0.22	0.68	0.63	0.93	0.41
$R22_{NiO}$	$HCO^*_{NiO} + H^*_{hCe} \rightleftharpoons CH_2O^*_{NiO} + *_{hCe}$	0.42	0.26	0.51	0.71	0.09	0.45
$R22_{NiCe}$	$HCO^*_{NiCe} + H^*_{hO} \rightleftharpoons CH_2O^*_{NiCe} + *_{hO}$	0.29	0.26	0.46	0.71	0.17	0.45
$R23_{NiO}$	$CH_2O^*_{NiO} + *_{NiCe} \rightleftharpoons CH^*_{2,NiCe} + O^*_{NiO}$	-0.19	-0.40	0.64	0.96	0.83	1.37
$R23_{NiCe}$	$CH_2O^*_{NiCe} + *_{NiO} \rightleftharpoons CH^*_{2,NiO} + O^*_{NiCe}$	-0.39	-0.40	0.68	0.96	1.07	1.37
$R24_{NiO}$	$CH_2O^*_{NiO} + H^*_{hO} \rightleftharpoons CH_2OH^*_{NiO} + *_{hO}$	-0.17	0.31	0.68	0.86	0.75	0.55
$R24_{NiCe}$	$CH_2O^*_{NiCe} + H^*_{hCe} \rightleftharpoons CH_2OH^*_{NiCe} + *_{hCe}$	-0.14	0.33	0.63	0.86	0.77	0.55
$R25_{NiO}$	$CH_2OH^*_{NiO} + *_{NiCe} \rightleftharpoons CH^*_{2,NiCe} + OH^*_{NiO}$	-0.93	-0.30	0.25	0.71	1.18	1.01
$R25_{NiCe}$	$CH_2OH^*_{NiCe} + *_{NiO} \rightleftharpoons CH^*_{2,NiO} + OH^*_{NiCe}$	-0.92	-0.30	0.34	0.71	1.26	1.01
$R26_{NiO}$	$CH_2O^*_{NiO} + H^*_{hCe} \rightleftharpoons CH_3O^*_{NiO} + *_{hCe}$	0.03	-0.50	0.65	0.34	0.62	0.85
$R26_{NiCe}$	$CH_2O^*_{NiCe} + H^*_{hO} \rightleftharpoons CH_3O^*_{NiCe} + *_{hO}$	-0.14	-0.50	0.40	0.34	0.53	0.85
$R27_{NiO}$	$CH_3O^*_{NiO} + *_{NiCe} \rightleftharpoons CH^*_{3,NiO} + O^*_{NiCe}$	-0.57	-0.16	0.98	1.36	1.55	1.52
$R27_{NiCe}$	$CH_3O^*_{NiCe} + *_{NiO} \rightleftharpoons CH^*_{3,NiCe} + O^*_{NiO}$	-0.39	-0.16	1.27	1.26	1.67	1.52
$R28_{NiO}$	$CH_3O^*_{NiO} + H^*_{hCe} \rightleftharpoons CH_3OH^*_{NiO} + *_{hCe}$	1.09	0.44	1.31	1.38	0.22	0.95
$R28_{NiCe}$	$CH_3O^*_{NiCe} + H^*_{hO} \rightleftharpoons CH_3OH^*_{NiCe} + *_{hO}$	1.11	0.44	1.33	1.38	0.22	0.95
$R29_{NiO}$	$CH_2OH^*_{NiO} + H^*_{hCe} \rightleftharpoons CH_3OH^*_{NiO} + *_{hCe}$	0.56	-0.42	1.20	0.61	0.64	1.03

$R29_{NiCe}$	$CH_2OH^*_{NiCe} + H^*_{hO} \rightleftharpoons CH_3OH^*_{NiCe} + *_{hO}$	0.48	-0.42	1.04	0.61	0.56	1.03
$R30_{NiO}$	$CH_3OH^*_{NiO} + *_{NiCe} \rightleftharpoons CH^*_{3,NiO} + OH^*_{NiCe}$	-1.76	-0.29	1.13	1.80	2.88	2.09
$R30_{NiCe}$	$CH_3OH^*_{NiCe} + *_{NiO} \rightleftharpoons CH^*_{3,NiCe} + OH^*_{NiO}$	-1.71	-0.29	1.09	1.80	2.80	2.09
$R31_{NiO,f}$	$CH^*_{3,NiO} + H^*_{hCe} \rightleftharpoons CH^*_{4,NiO} + *_{hCe}$	0.85	-0.30	1.00	0.79	0.15	0.96
$R31_{NiO,n}$	$CH^*_{3,NiO} + H^*_{hO} \rightleftharpoons CH^*_{4,NiO} + *_{hO}$	0.43	-0.30	0.71	0.79	0.28	0.96
$R31_{NiCe,f}$	$CH^*_{3,NiCe} + H^*_{hO} \rightleftharpoons CH^*_{4,NiCe} + *_{hO}$	0.77	-0.30	0.91	0.79	0.14	0.96
$R31_{NiCe,n}$	$CH^*_{3,NiCe} + H^*_{hCe} \rightleftharpoons CH^*_{4,NiCe} + *_{hCe}$	0.52	-0.30	0.77	0.79	0.25	0.96
$R32_{NiO,f}$	$O^*_{NiO} + H^*_{hCe} \rightleftharpoons OH^*_{NiO} + *_{hCe}$	0.06	0.04	1.00	1.16	0.94	1.12
$R32_{NiCe,f}$	$O^*_{NiCe} + H^*_{hO} \rightleftharpoons OH^*_{NiCe} + *_{hO}$	0.04	0.04	0.99	1.16	0.95	1.12
$R32_{NiO,n}$	$O^*_{NiO} + H^*_{hO} \rightleftharpoons OH^*_{NiO} + *_{hO}$	-0.42	0.04	0.61	1.16	1.03	1.12
$R32_{NiCe,n}$	$O^*_{NiCe} + H^*_{hCe} \rightleftharpoons OH^*_{NiCe} + *_{hCe}$	-0.30	0.04	0.66	1.16	0.96	1.12
$R33_{NiO,f}$	$OH^*_{NiO} + H^*_{hCe} \rightleftharpoons H_2O^*_{NiO} + *_{hCe}$	1.03	0.25	1.27	1.16	0.24	0.91
$R33_{NiCe,f}$	$OH^*_{NiCe} + H^*_{hO} \rightleftharpoons H_2O^*_{NiCe} + *_{hO}$	1.02	0.25	1.26	1.16	0.24	0.91
$R33_{NiO,n}$	$OH^*_{NiO} + H^*_{hO} \rightleftharpoons H_2O^*_{NiO} + *_{hO}$	0.50	0.25	0.76	1.16	0.26	0.91
$R33_{NiCe,n}$	$OH^*_{NiCe} + H^*_{hCe} \rightleftharpoons H_2O^*_{NiCe} + *_{hCe}$	0.64	0.25	0.99	1.16	0.35	0.91
$R34_{NiO}$	$H_{2,(g)} + O^*_{NiO} + *_{hO} \rightleftharpoons OH^*_{NiO} + H^*_{hO}$	-0.24		0.53		0.77	
$R34_{NiCe}$	$H_{2,(g)} + O^*_{NiCe} + *_{hCe} \rightleftharpoons OH^*_{NiCe} + H^*_{hCe}$	-0.15		0.48		0.63	
$R35_{NiO}$	$H_{2,(g)} + OH^*_{NiO} + *_{hO} \rightleftharpoons H_2O^*_{NiO} + H^*_{hO}$	0.69		1.32		0.63	
$R35_{NiCe}$	$H_{2,(g)} + OH^*_{NiCe} + *_{hCe} \rightleftharpoons H_2O^*_{NiCe} + H^*_{hCe}$	0.63		0.63		0.0	
$R36_{NiO}$	$CO^*_{NiO} \rightleftharpoons CO_{(g)} + *_{NiO}$	2.33	1.61	2.33	1.61	0.0	0.0
$R36_{NiCe}$	$CO^*_{NiCe} \rightleftharpoons CO_{(g)} + *_{NiCe}$	2.47	1.61	2.47	1.61	0.0	0.0
$R37_{NiO}$	$CH_2O^*_{NiO} \rightleftharpoons CH_2O_{(g)} + *_{NiO}$	2.06	0.58	2.06	0.58	0.0	0.0
$R37_{NiCe}$	$CH_2O^*_{NiCe} \rightleftharpoons CH_2O_{(g)} + *_{NiCe}$	2.10	0.58	2.10	0.58	0.0	0.0
$R38_{NiO}$	$CH_3OH^*_{NiO} \rightleftharpoons CH_3OH_{(g)} + *_{NiO}$	0.91	0.36	0.91	0.36	0.0	0.0
$R38_{NiCe}$	$CH_3OH^*_{NiCe} \rightleftharpoons CH_3OH_{(g)} + *_{NiCe}$	0.89	0.36	0.89	0.36	0.0	0.0
$R39_{NiO}$	$CH^*_{4,NiO} \rightleftharpoons CH_{4,(g)} + *_{NiO}$	0.26	0.13	0.26	0.13	0.0	0.0
$R39_{NiCe}$	$CH^*_{4,NiCe} \rightleftharpoons CH_{4,(g)} + *_{NiCe}$	0.26	0.13	0.26	0.13	0.0	0.0
$R40_{NiO}$	$H_2O^*_{NiO} \rightleftharpoons H_2O_{(g)} + *_{NiO}$	0.75	0.26	0.75	0.26	0.0	0.0
$R40_{NiCe}$	$H_2O^*_{NiCe} \rightleftharpoons H_2O_{(g)} + *_{NiCe}$	0.73	0.26	0.73	0.26	0.0	0.0
D1	$H^*_{NiCe} + *_{NiO} \rightleftharpoons H^*_{NiO} + *_{NiCe}$	0.03	0.02	0.16	0.12	0.13	0.10
D2	$CO^*_{NiCe} + *_{NiO} \rightleftharpoons CO^*_{NiO} + *_{NiCe}$	0.14	-0.01	0.19	0.11	0.05	0.12
D3	$O^*_{NiCe} + *_{NiO} \rightleftharpoons O^*_{NiO} + *_{NiCe}$	0.22	0.10	0.60	0.42	0.38	0.32
D4	$OH^*_{NiCe} + *_{NiO} \rightleftharpoons OH^*_{NiO} + *_{NiCe}$	0.09	0.09	0.44	0.19	0.35	0.10
D5	$C^*_{NiCe} + *_{NiO} \rightleftharpoons C^*_{NiO} + *_{NiCe}$	0.08	-0.04	1.70	0.31	1.62	0.35
D6	$CH^*_{NiCe} + *_{NiO} \rightleftharpoons CH^*_{NiO} + *_{NiCe}$	0.19	0.02	0.62	0.32	0.44	0.30
D7	$CH^*_{2,NiCe} + *_{NiO} \rightleftharpoons CH^*_{2,NiO} + *_{NiCe}$	0.13	0.04	0.48	0.19	0.35	0.15

D8	$CH^*_{3,NiCe} + *_{NiO} \rightleftharpoons CH^*_{3,NiO} + *_{NiCe}$	0.06	0.02	0.33	0.15	0.27	0.13

Table S4. Species Coverage at the Different Considered Sites for the Simulations with the Eley-Rideal Reactions (W/ER) and without Them (Wo/ER) at the Five Different Temperatures Considered and at P(H_2) = 0.528 bar and P(CO_2) = 0.132 bar. Note that the Present Values are Calculated as the Mean Value of 5 Different kMC Simulations for Each Working Condition.

**T=483K**	**Ni_{Ce}**		**Ni_O**		**h_{Ce}**		**h_O**	
**Species**	**W/ER**	**Wo/ER**	**W/ER**	**Wo/ER**	**W/ER**	**Wo/ER**	**W/ER**	**Wo/ER**
**CO**	70.0	67.4	28.8	28.0	-	-	-	-
**O**	0.2	5.3	0	1.7	-	-	-	-
**OH**	1.1	0.9	0	0	-	-	-	-
**CH_3**	0.1	0	0.2	0				
**H_2**	-	-	-	-	35.3	36.1	35.3	36.1
**H**	-	-	-	-	17.5	15.5	16.1	14.1
**Total**	71.6	73.6	30.0	29.7	52.8	51.6	51.4	50.2
**T=503K**	**Ni_{Ce}**		**Ni_O**		**h_{Ce}**		**h_O**	
**Species**	**W/ER**	**Wo/ER**	**W/ER**	**Wo/ER**	**W/ER**	**Wo/ER**	**W/ER**	**Wo/ER**
**CO**	72.0	67.4	27.1	28.8	-	-	-	-
**O**	0.5	5.3	0	1.1	-	-	-	-
**OH**	0.7	1.0	0	0.1	-	-	-	-
**H_2**	-	-	-	-	32.4	32.8	32.4	32.8
**H**	-	-	-	-	18.2	17.0	16.6	15.4
**Total**	73.2	73.7	27.1	30.0	50.6	49.8	49.0	48.2
**T=523K**	**Ni_{Ce}**		**Ni_O**		**h_{Ce}**		**h_O**	
**Species**	**W/ER**	**Wo/ER**	**W/ER**	**Wo/ER**	**W/ER**	**Wo/ER**	**W/ER**	**Wo/ER**
**CO**	71.7	67.7	27.0	30.2	-	-	-	-
**O**	0.7	6.6	0	0.5	-	-	-	-
**OH**	0.7	0.5	0	0	-	-	-	-
**H_2**	-	-	-	-	29.0	29.3	29.0	29.3
**H**	-	-	-	-	18.9	17.3	17.0	15.6
**Total**	73.1	74.8	27.0	30.7	47.9	46.6	46.0	44.9

T=543K	Ni_{Ce}		Ni_O		h_{Ce}		h_O	
**Species**	**W/ER**	**Wo/ER**	**W/ER**	**Wo/ER**	**W/ER**	**Wo/ER**	**W/ER**	**Wo/ER**
**CO**	71.1	64.0	26.9	33.2	-	-	-	-
**O**	1.1	10.7	0	0.3	-	-	-	-
**OH**	0.8	0.8	0	0	-	-	-	-
**H_2**	-	-	-	-	25.3	25.6	25.3	25.6
**H**	-	-	-	-	19.0	17.0	17.2	15.3
**Total**	73.0	74.7	26.9	33.5	44.3	42.6	42.5	40.9
**T=563K**	**Ni_{Ce}**		**Ni_O**		**h_{Ce}**		**h_O**	
**Species**	**W/ER**	**Wo/ER**	**W/ER**	**Wo/ER**	**W/ER**	**Wo/ER**	**W/ER**	**Wo/ER**
**CO_2**	0.9	0.7	0.8	0.7	-	-	-	-
**CO**	69.9	60.9	25.8	34.8	-	-	-	-
**O**	1.0	13.7	0	0	-	-	-	-
**OH**	0.5	0.5	0	0	-	-	-	-
**H_2**	-	-	-	-	21.5	22.0	21.5	22.0
**H**	-	-	-	-	19.3	16.6	17.4	15.3
**Total**	72.3	75.8	26.6	35.5	40.8	38.6	38.9	37.3

Plots summarizing the event frequencies for the different sites at the different temperature conditions for the simulations with the ER reactions (W/ER) and without the ER reactions (Wo/ER)

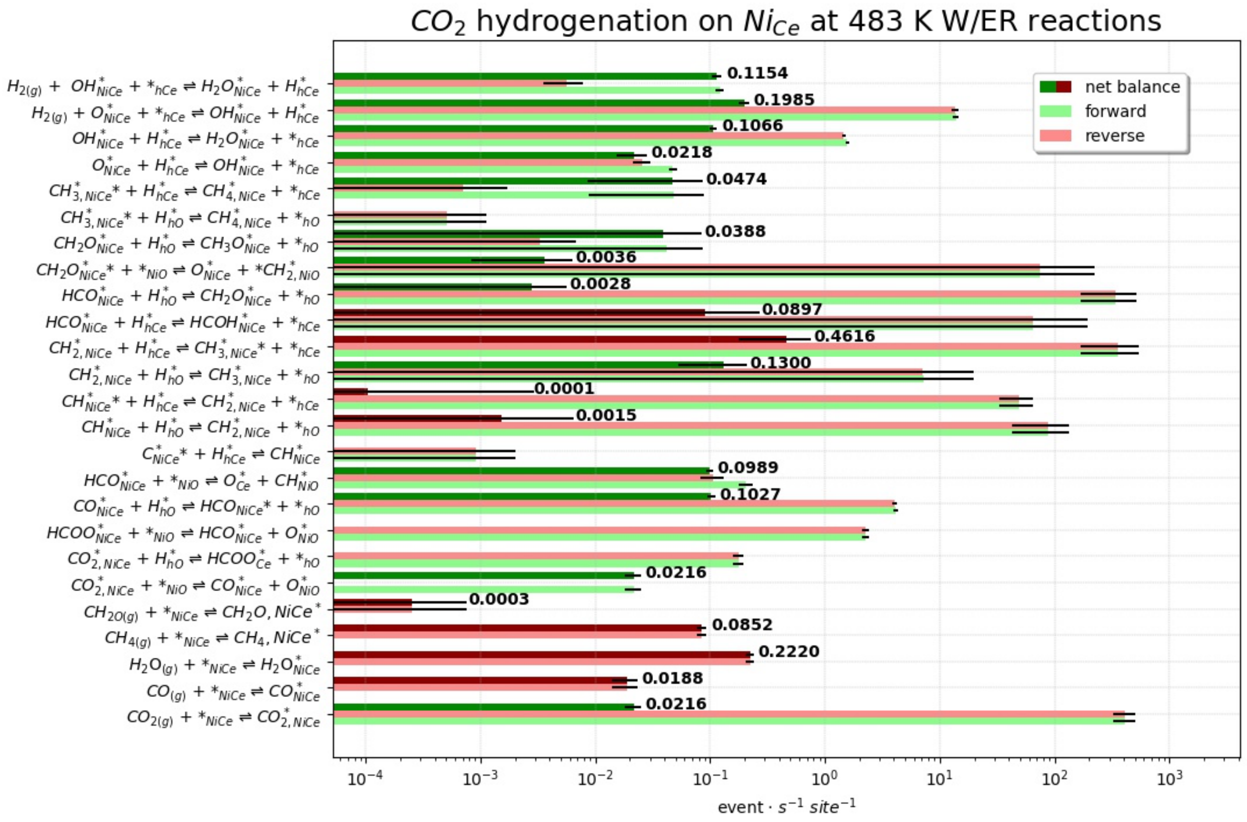

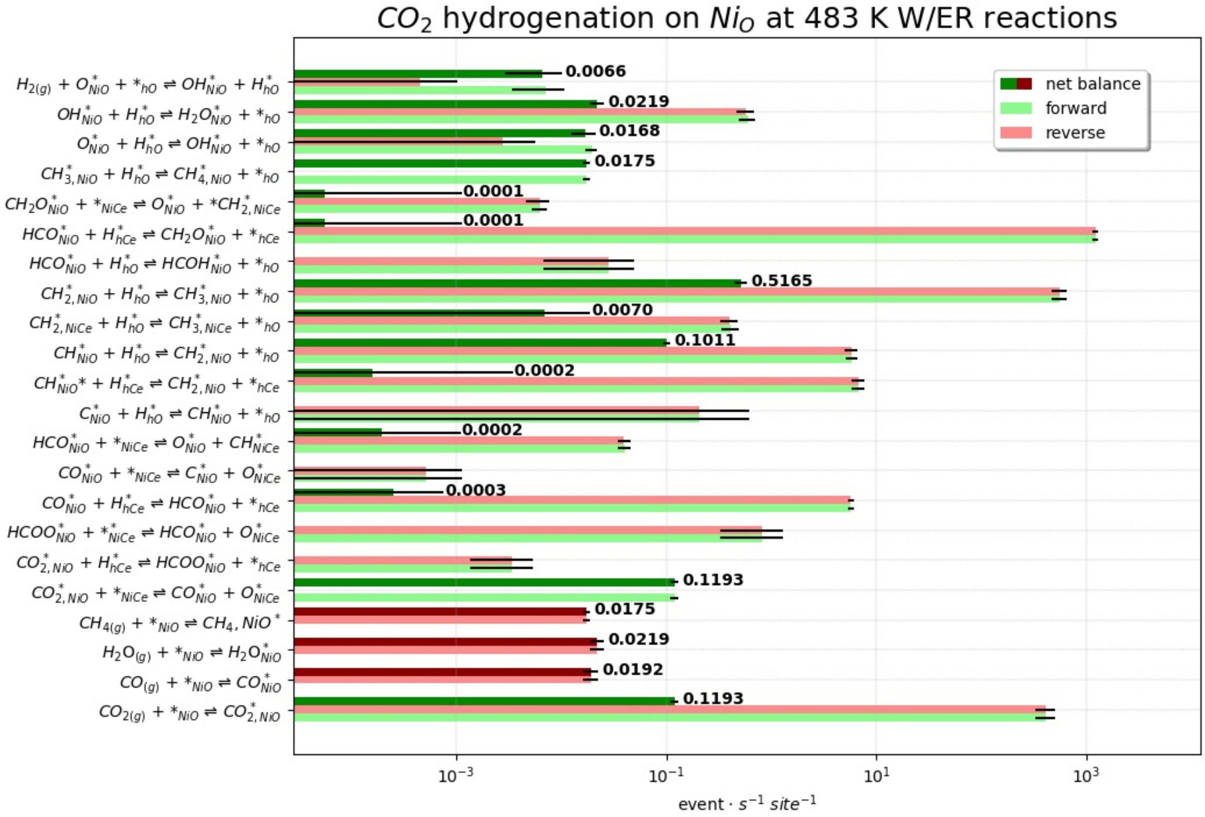
CO2 hydrogenation on NiO at 483 K W/ER reactions
net balance
forward
reverse
0.0066
0.0219
0.0168
0.0175
0.0001
0.0001
0.5165
0.0070
0.1011
0.0002
0.0002
0.0003
0.1193
0.0175
0.0219
0.0192
0.1193
10−3
10−1
10¹
10³
event · s−1 site−1

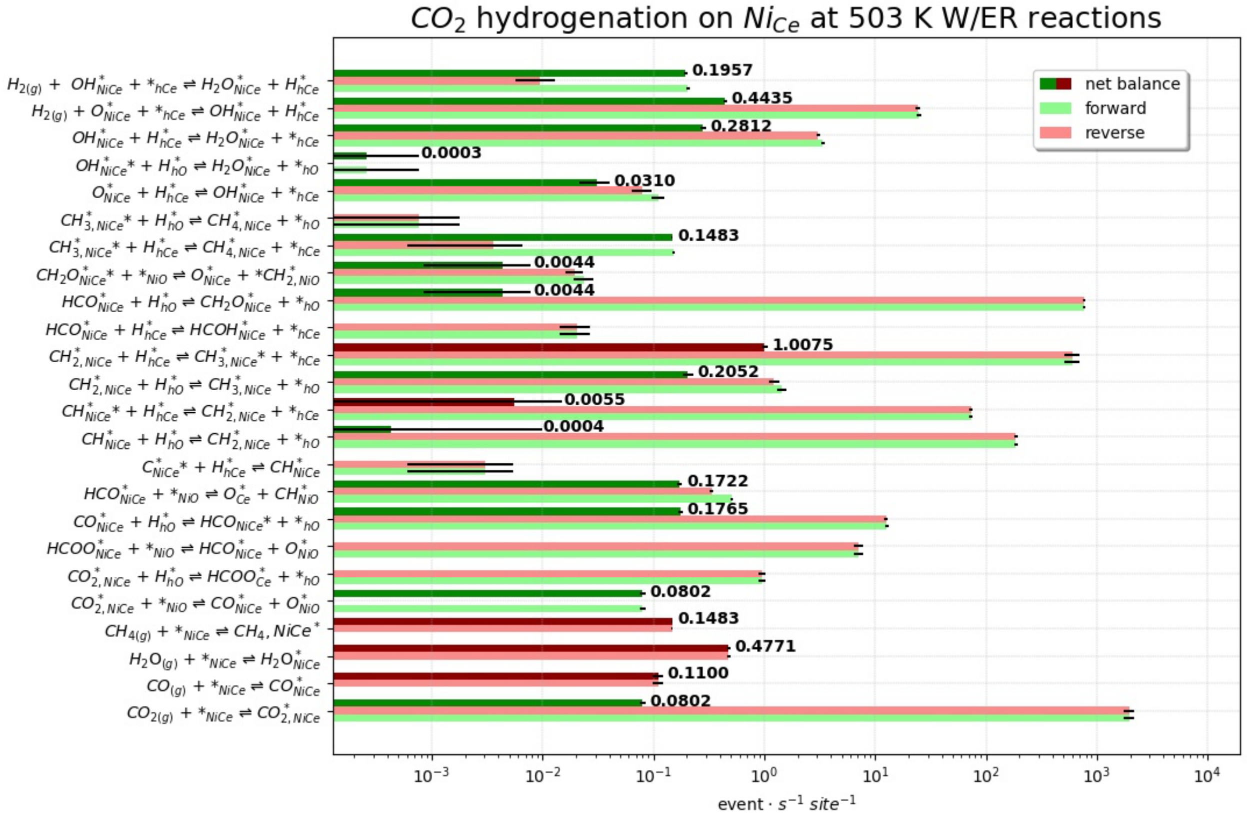
CO_2 hydrogenation on Ni_{Ce} at 503 K W/ER reactions
$H_{2(g)} + OH^*_{NiCe} + *_{hCe} \rightleftharpoons H_2O^*_{NiCe} + H^*_{hCe}$
$H_{2(g)} + O^*_{NiCe} + *_{hCe} \rightleftharpoons OH^*_{NiCe} + H^*_{hCe}$
$OH^*_{NiCe} + H^*_{hCe} \rightleftharpoons H_2O^*_{NiCe} + *_{hCe}$
$OH^*_{NiCe}* + H^*_{hO} \rightleftharpoons H_2O^*_{NiCe} + *_{hO}$
$O^*_{NiCe} + H^*_{hCe} \rightleftharpoons OH^*_{NiCe} + *_{hCe}$
$CH^*_{3,NiCe}* + H^*_{hO} \rightleftharpoons CH^*_{4,NiCe} + *_{hO}$
$CH^*_{3,NiCe}* + H^*_{hCe} \rightleftharpoons CH^*_{4,NiCe} + *_{hCe}$
$CH_2O^*_{NiCe}* + *_{NiO} \rightleftharpoons O^*_{NiCe} + *CH^*_{2,NiO}$
$HCO^*_{NiCe} + H^*_{hO} \rightleftharpoons CH_2O^*_{NiCe} + *_{hO}$
$HCO^*_{NiCe} + H^*_{hCe} \rightleftharpoons HCOH^*_{NiCe} + *_{hCe}$
$CH^*_{2,NiCe} + H^*_{hCe} \rightleftharpoons CH^*_{3,NiCe}* + *_{hCe}$
$CH^*_{2,NiCe} + H^*_{hO} \rightleftharpoons CH^*_{3,NiCe} + *_{hO}$
$CH^*_{NiCe}* + H^*_{hCe} \rightleftharpoons CH^*_{2,NiCe} + *_{hCe}$
$CH^*_{NiCe} + H^*_{hO} \rightleftharpoons CH^*_{2,NiCe} + *_{hO}$
$C^*_{NiCe}* + H^*_{hCe} \rightleftharpoons CH^*_{NiCe}$
$HCO^*_{NiCe} + *_{NiO} \rightleftharpoons O^*_{Ce} + CH^*_{NiO}$
$CO^*_{NiCe} + H^*_{hO} \rightleftharpoons HCO_{NiCe}* + *_{hO}$
$HCOO^*_{NiCe} + *_{NiO} \rightleftharpoons HCO^*_{NiCe} + O^*_{NiO}$
$CO^*_{2,NiCe} + H^*_{hO} \rightleftharpoons HCOO^*_{Ce} + *_{hO}$
$CO^*_{2,NiCe} + *_{NiO} \rightleftharpoons CO^*_{NiCe} + O^*_{NiO}$
$CH_{4(g)} + *_{NiCe} \rightleftharpoons CH_4, NiCe^*$
$H_2O_{(g)} + *_{NiCe} \rightleftharpoons H_2O^*_{NiCe}$
$CO_{(g)} + *_{NiCe} \rightleftharpoons CO^*_{NiCe}$
$CO_{2(g)} + *_{NiCe} \rightleftharpoons CO^*_{2,NiCe}$
0.1957
0.4435
0.2812
0.0003
0.0310
0.1483
0.0044
0.0044
1.0075
0.2052
0.0055
0.0004
0.1722
0.1765
0.0802
0.1483
0.4771
0.1100
0.0802
net balance
forward
reverse
10^{-3} 10^{-2} 10^{-1} 10^{0} 10^{1} 10^{2} 10^{3} 10^{4}
event $\cdot s^{-1}$ $site^{-1}$

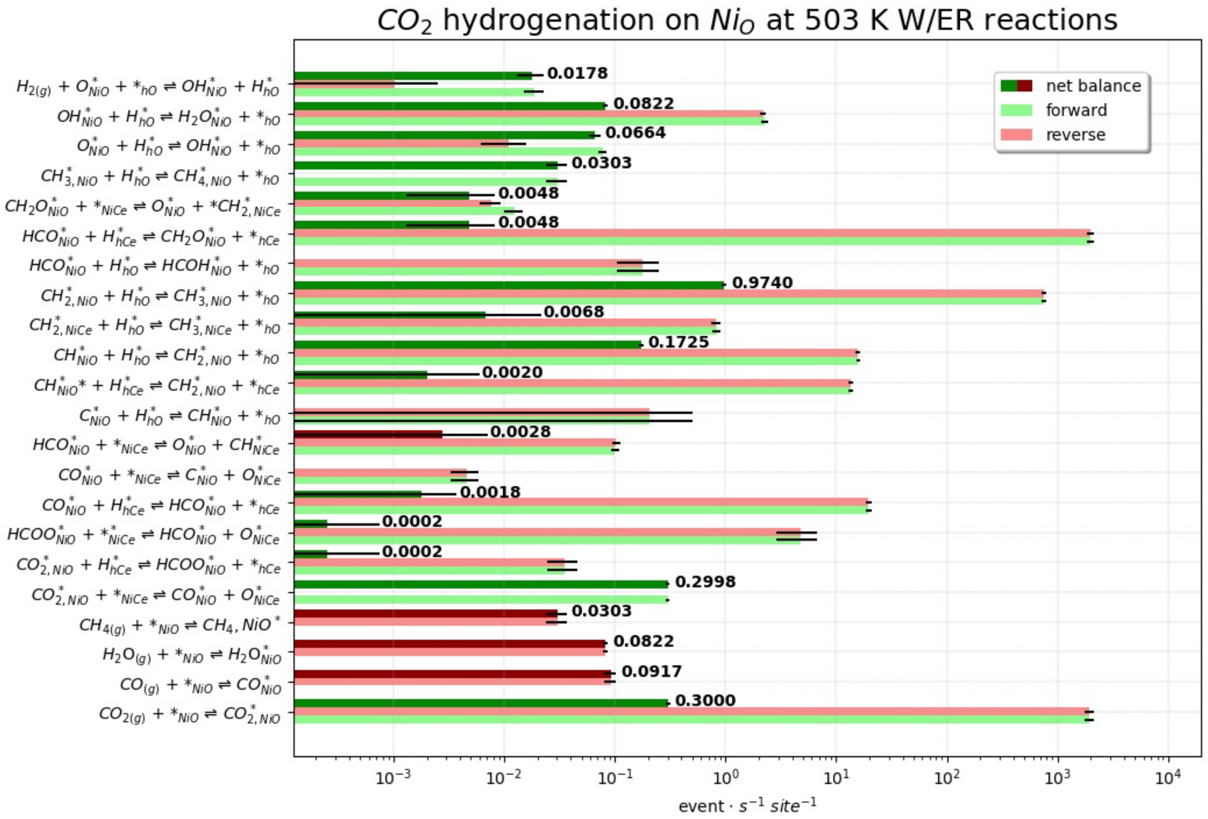
CO_2 hydrogenation on Ni_O at 503 K W/ER reactions
$H_{2(g)} + O^*_{NiO} + *_{hO} \rightleftharpoons OH^*_{NiO} + H^*_{hO}$
$OH^*_{NiO} + H^*_{hO} \rightleftharpoons H_2O^*_{NiO} + *_{hO}$
$O^*_{NiO} + H^*_{hO} \rightleftharpoons OH^*_{NiO} + *_{hO}$
$CH^*_{3,NiO} + H^*_{hO} \rightleftharpoons CH^*_{4,NiO} + *_{hO}$
$CH_2O^*_{NiO} + *_{NiCe} \rightleftharpoons O^*_{NiO} + *CH^*_{2,NiCe}$
$HCO^*_{NiO} + H^*_{hCe} \rightleftharpoons CH_2O^*_{NiO} + *_{hCe}$
$HCO^*_{NiO} + H^*_{hO} \rightleftharpoons HCOH^*_{NiO} + *_{hO}$
$CH^*_{2,NiO} + H^*_{hO} \rightleftharpoons CH^*_{3,NiO} + *_{hO}$
$CH^*_{2,NiCe} + H^*_{hO} \rightleftharpoons CH^*_{3,NiCe} + *_{hO}$
$CH^*_{NiO} + H^*_{hO} \rightleftharpoons CH^*_{2,NiO} + *_{hO}$
$CH^*_{NiO}* + H^*_{hCe} \rightleftharpoons CH^*_{2,NiO} + *_{hCe}$
$C^*_{NiO} + H^*_{hO} \rightleftharpoons CH^*_{NiO} + *_{hO}$
$HCO^*_{NiO} + *_{NiCe} \rightleftharpoons O^*_{NiO} + CH^*_{NiCe}$
$CO^*_{NiO} + *_{NiCe} \rightleftharpoons C^*_{NiO} + O^*_{NiCe}$
$CO^*_{NiO} + H^*_{hCe} \rightleftharpoons HCO^*_{NiO} + *_{hCe}$
$HCOO^*_{NiO} + *_{NiCe} \rightleftharpoons HCO^*_{NiO} + O^*_{NiCe}$
$CO^*_{2,NiO} + H^*_{hCe} \rightleftharpoons HCOO^*_{NiO} + *_{hCe}$
$CO^*_{2,NiO} + *_{NiCe} \rightleftharpoons CO^*_{NiO} + O^*_{NiCe}$
$CH_{4(g)} + *_{NiO} \rightleftharpoons CH_4, NiO^*$
$H_2O_{(g)} + *_{NiO} \rightleftharpoons H_2O^*_{NiO}$
$CO_{(g)} + *_{NiO} \rightleftharpoons CO^*_{NiO}$
$CO_{2(g)} + *_{NiO} \rightleftharpoons CO^*_{2,NiO}$
0.0178
0.0822
0.0664
0.0303
0.0048
0.0048
0.9740
0.0068
0.1725
0.0020
0.0028
0.0018
0.0002
0.0002
0.2998
0.0303
0.0822
0.0917
0.3000
net balance
forward
reverse
10^{-3} 10^{-2} 10^{-1} 10^{0} 10^{1} 10^{2} 10^{3} 10^{4}
event $\cdot s^{-1}$ $site^{-1}$

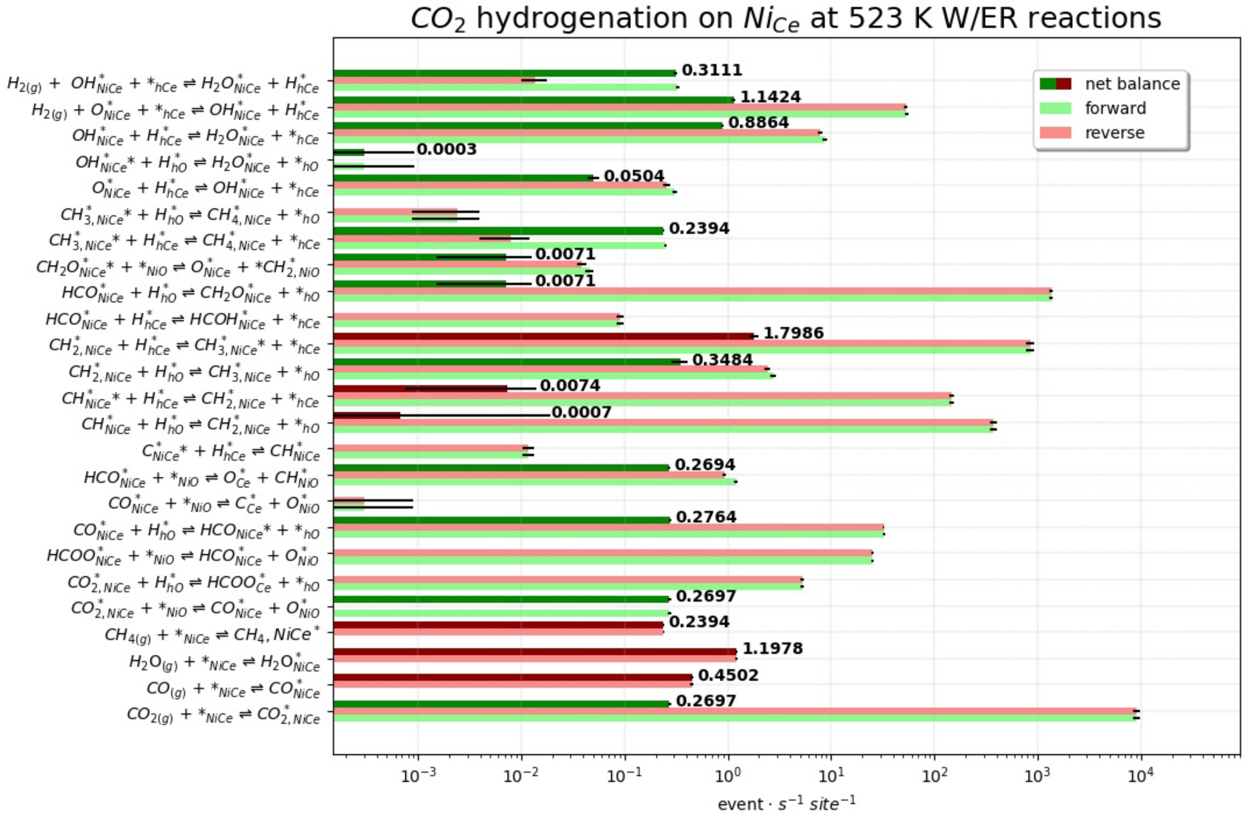
CO2 hydrogenation on NiCe at 523 K W/ER reactions
net balance
forward
reverse
0.3111
1.1424
0.8864
0.0003
0.0504
0.2394
0.0071
0.0071
1.7986
0.3484
0.0074
0.0007
0.2694
0.2764
0.2697
0.2394
1.1978
0.4502
0.2697
event · s−1 site−1

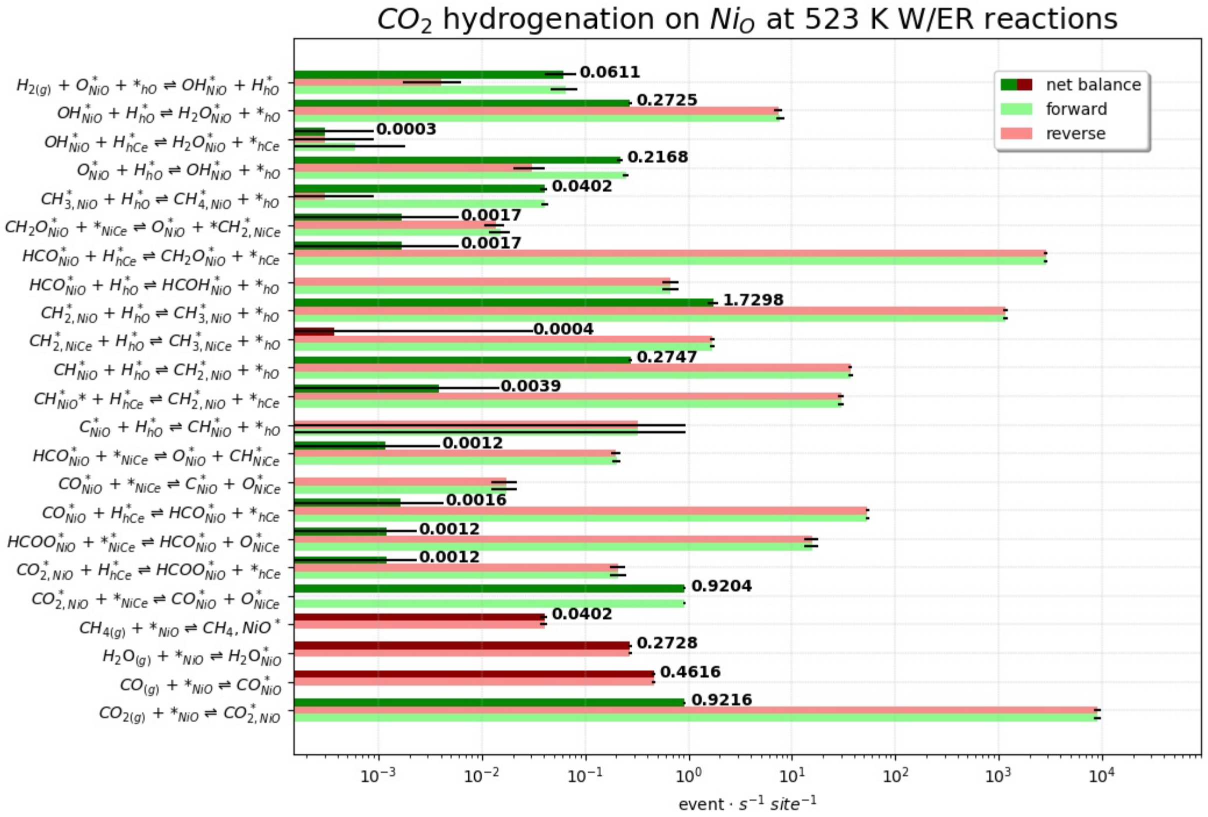
CO2 hydrogenation on NiO at 523 K W/ER reactions
net balance
forward
reverse
0.0611
0.2725
0.0003
0.2168
0.0402
0.0017
0.0017
1.7298
0.0004
0.2747
0.0039
0.0012
0.0016
0.0012
0.0012
0.9204
0.0402
0.2728
0.4616
0.9216
event · s−1 site−1

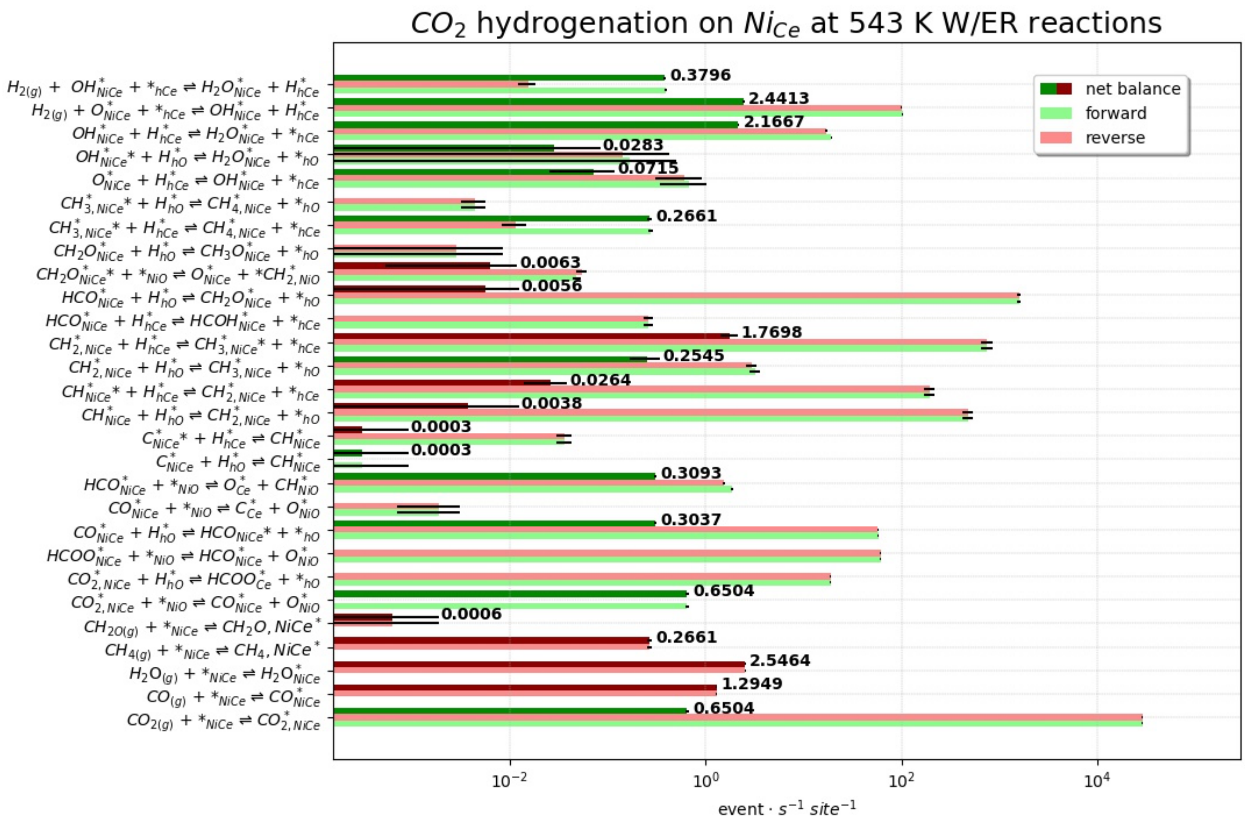

CO_2 hydrogenation on Ni_{Ce} at 543 K W/ER reactions
$H_{2(g)} + OH^*_{NiCe} + *_{hCe} \rightleftharpoons H_2O^*_{NiCe} + H^*_{hCe}$
$H_{2(g)} + O^*_{NiCe} + *_{hCe} \rightleftharpoons OH^*_{NiCe} + H^*_{hCe}$
$OH^*_{NiCe} + H^*_{hCe} \rightleftharpoons H_2O^*_{NiCe} + *_{hCe}$
$OH^*_{NiCe}* + H^*_{hO} \rightleftharpoons H_2O^*_{NiCe} + *_{hO}$
$O^*_{NiCe} + H^*_{hCe} \rightleftharpoons OH^*_{NiCe} + *_{hCe}$
$CH^*_{3,NiCe}* + H^*_{hO} \rightleftharpoons CH^*_{4,NiCe} + *_{hO}$
$CH^*_{3,NiCe}* + H^*_{hCe} \rightleftharpoons CH^*_{4,NiCe} + *_{hCe}$
$CH_2O^*_{NiCe} + H^*_{hO} \rightleftharpoons CH_3O^*_{NiCe} + *_{hO}$
$CH_2O^*_{NiCe}* + *_{NiO} \rightleftharpoons O^*_{NiCe} + *CH^*_{2,NiO}$
$HCO^*_{NiCe} + H^*_{hO} \rightleftharpoons CH_2O^*_{NiCe} + *_{hO}$
$HCO^*_{NiCe} + H^*_{hCe} \rightleftharpoons HCOH^*_{NiCe} + *_{hCe}$
$CH^*_{2,NiCe} + H^*_{hCe} \rightleftharpoons CH^*_{3,NiCe}* + *_{hCe}$
$CH^*_{2,NiCe} + H^*_{hO} \rightleftharpoons CH^*_{3,NiCe} + *_{hO}$
$CH^*_{NiCe}* + H^*_{hCe} \rightleftharpoons CH^*_{2,NiCe} + *_{hCe}$
$CH^*_{NiCe} + H^*_{hO} \rightleftharpoons CH^*_{2,NiCe} + *_{hO}$
$C^*_{NiCe}* + H^*_{hCe} \rightleftharpoons CH^*_{NiCe}$
$C^*_{NiCe} + H^*_{hO} \rightleftharpoons CH^*_{NiCe}$
$HCO^*_{NiCe} + *_{NiO} \rightleftharpoons O^*_{Ce} + CH^*_{NiO}$
$CO^*_{NiCe} + *_{NiO} \rightleftharpoons C^*_{Ce} + O^*_{NiO}$
$CO^*_{NiCe} + H^*_{hO} \rightleftharpoons HCO_{NiCe}* + *_{hO}$
$HCOO^*_{NiCe} + *_{NiO} \rightleftharpoons HCO^*_{NiCe} + O^*_{NiO}$
$CO^*_{2,NiCe} + H^*_{hO} \rightleftharpoons HCOO^*_{Ce} + *_{hO}$
$CO^*_{2,NiCe} + *_{NiO} \rightleftharpoons CO^*_{NiCe} + O^*_{NiO}$
$CH_{2O(g)} + *_{NiCe} \rightleftharpoons CH_2O, NiCe^*$
$CH_{4(g)} + *_{NiCe} \rightleftharpoons CH_4, NiCe^*$
$H_2O_{(g)} + *_{NiCe} \rightleftharpoons H_2O^*_{NiCe}$
$CO_{(g)} + *_{NiCe} \rightleftharpoons CO^*_{NiCe}$
$CO_{2(g)} + *_{NiCe} \rightleftharpoons CO^*_{2,NiCe}$
0.3796
2.4413
2.1667
0.0283
0.0715
0.2661
0.0063
0.0056
1.7698
0.2545
0.0264
0.0038
0.0003
0.0003
0.3093
0.3037
0.6504
0.0006
0.2661
2.5464
1.2949
0.6504
net balance
forward
reverse
10^{-2}
10^0
10^2
10^4
event · s^{-1} $site^{-1}$

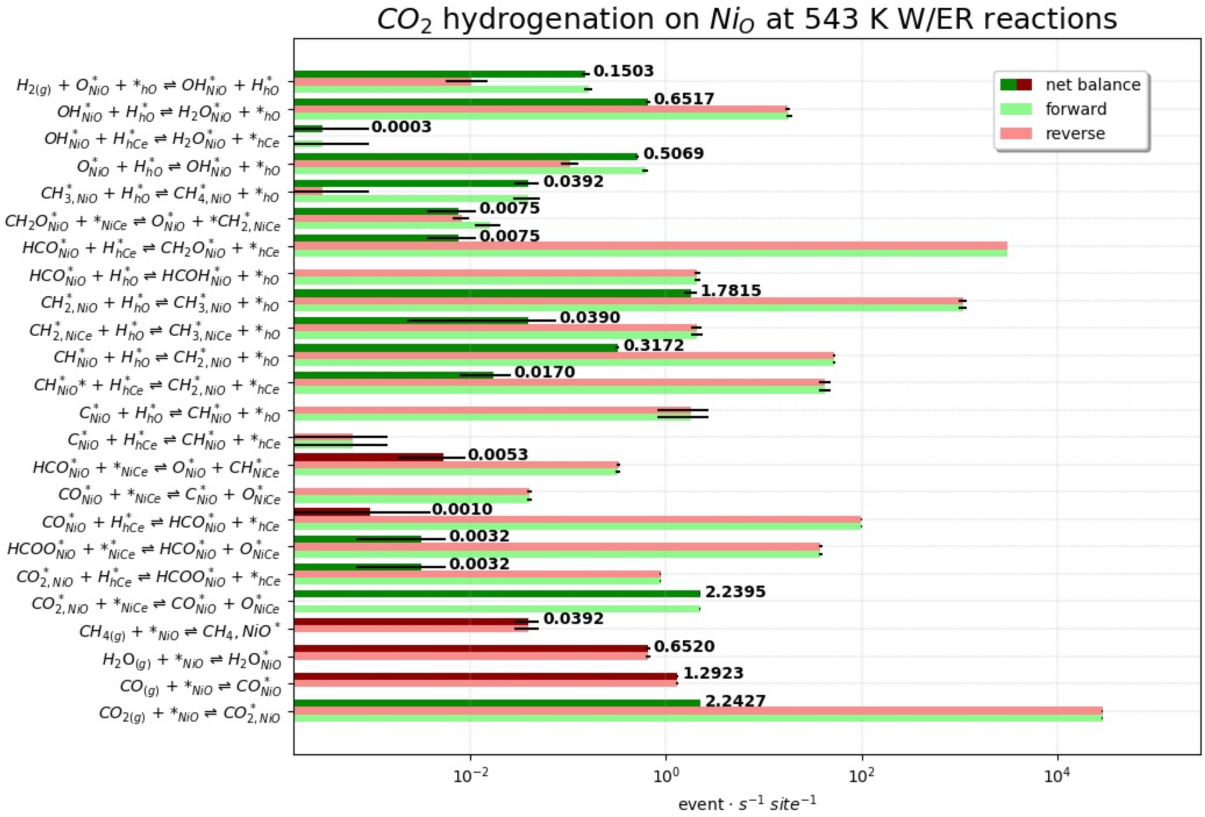

CO_2 hydrogenation on Ni_O at 543 K W/ER reactions
$H_{2(g)} + O^*_{NiO} + *_{hO} \rightleftharpoons OH^*_{NiO} + H^*_{hO}$
$OH^*_{NiO} + H^*_{hO} \rightleftharpoons H_2O^*_{NiO} + *_{hO}$
$OH^*_{NiO} + H^*_{hCe} \rightleftharpoons H_2O^*_{NiO} + *_{hCe}$
$O^*_{NiO} + H^*_{hO} \rightleftharpoons OH^*_{NiO} + *_{hO}$
$CH^*_{3,NiO} + H^*_{hO} \rightleftharpoons CH^*_{4,NiO} + *_{hO}$
$CH_2O^*_{NiO} + *_{NiCe} \rightleftharpoons O^*_{NiO} + *CH^*_{2,NiCe}$
$HCO^*_{NiO} + H^*_{hCe} \rightleftharpoons CH_2O^*_{NiO} + *_{hCe}$
$HCO^*_{NiO} + H^*_{hO} \rightleftharpoons HCOH^*_{NiO} + *_{hO}$
$CH^*_{2,NiO} + H^*_{hO} \rightleftharpoons CH^*_{3,NiO} + *_{hO}$
$CH^*_{2,NiCe} + H^*_{hO} \rightleftharpoons CH^*_{3,NiCe} + *_{hO}$
$CH^*_{NiO} + H^*_{hO} \rightleftharpoons CH^*_{2,NiO} + *_{hO}$
$CH^*_{NiO}* + H^*_{hCe} \rightleftharpoons CH^*_{2,NiO} + *_{hCe}$
$C^*_{NiO} + H^*_{hO} \rightleftharpoons CH^*_{NiO} + *_{hO}$
$C^*_{NiO} + H^*_{hCe} \rightleftharpoons CH^*_{NiO} + *_{hCe}$
$HCO^*_{NiO} + *_{NiCe} \rightleftharpoons O^*_{NiO} + CH^*_{NiCe}$
$CO^*_{NiO} + *_{NiCe} \rightleftharpoons C^*_{NiO} + O^*_{NiCe}$
$CO^*_{NiO} + H^*_{hCe} \rightleftharpoons HCO^*_{NiO} + *_{hCe}$
$HCOO^*_{NiO} + *_{NiCe} \rightleftharpoons HCO^*_{NiO} + O^*_{NiCe}$
$CO^*_{2,NiO} + H^*_{hCe} \rightleftharpoons HCOO^*_{NiO} + *_{hCe}$
$CO^*_{2,NiO} + *_{NiCe} \rightleftharpoons CO^*_{NiO} + O^*_{NiCe}$
$CH_{4(g)} + *_{NiO} \rightleftharpoons CH_4, NiO^*$
$H_2O_{(g)} + *_{NiO} \rightleftharpoons H_2O^*_{NiO}$
$CO_{(g)} + *_{NiO} \rightleftharpoons CO^*_{NiO}$
$CO_{2(g)} + *_{NiO} \rightleftharpoons CO^*_{2,NiO}$
0.1503
0.6517
0.0003
0.5069
0.0392
0.0075
0.0075
1.7815
0.0390
0.3172
0.0170
0.0053
0.0010
0.0032
0.0032
2.2395
0.0392
0.6520
1.2923
2.2427
net balance
forward
reverse
10^{-2}
10^0
10^2
10^4
event · s^{-1} $site^{-1}$

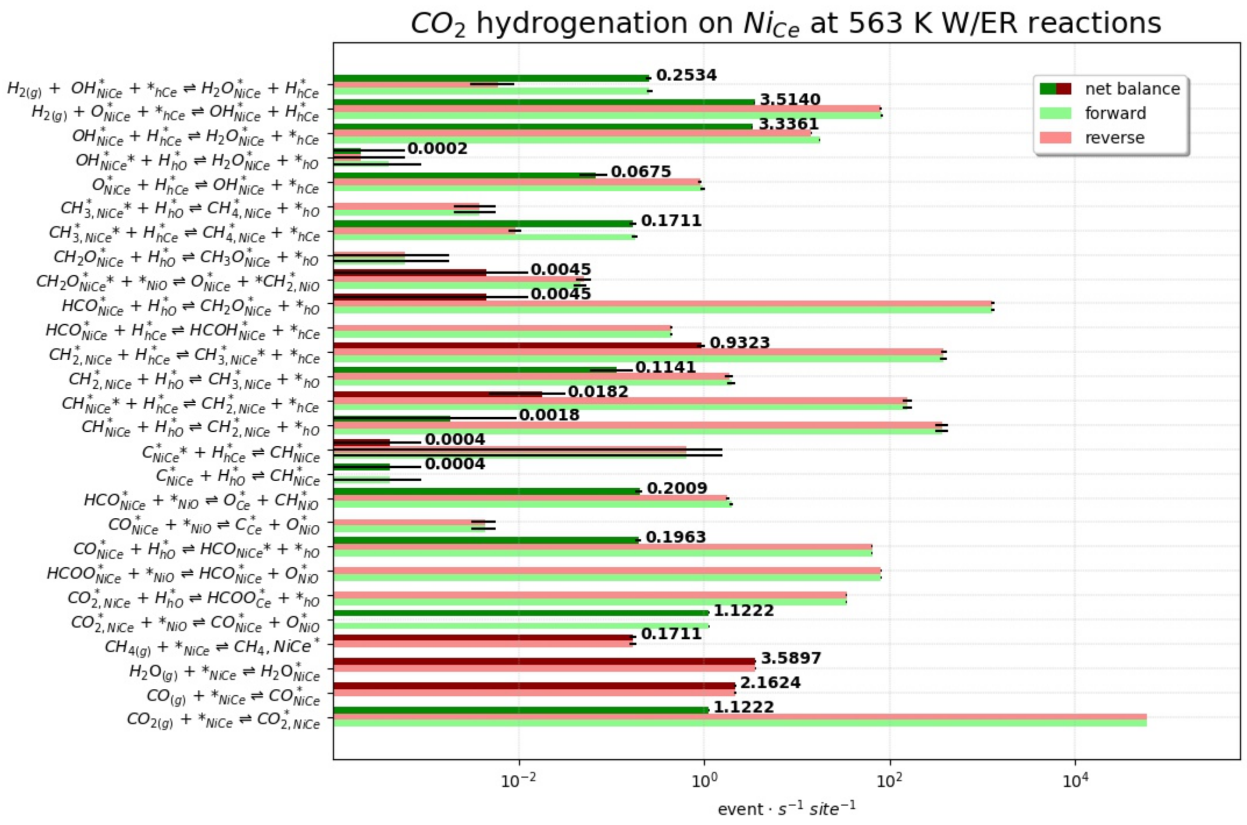
CO_2 hydrogenation on Ni_{Ce} at 563 K W/ER reactions
$H_{2(g)} + OH^*_{NiCe} + *_{hCe} \rightleftharpoons H_2O^*_{NiCe} + H^*_{hCe}$
$H_{2(g)} + O^*_{NiCe} + *_{hCe} \rightleftharpoons OH^*_{NiCe} + H^*_{hCe}$
$OH^*_{NiCe} + H^*_{hCe} \rightleftharpoons H_2O^*_{NiCe} + *_{hCe}$
$OH^*_{NiCe}* + H^*_{hO} \rightleftharpoons H_2O^*_{NiCe} + *_{hO}$
$O^*_{NiCe} + H^*_{hCe} \rightleftharpoons OH^*_{NiCe} + *_{hCe}$
$CH^*_{3,NiCe}* + H^*_{hO} \rightleftharpoons CH^*_{4,NiCe} + *_{hO}$
$CH^*_{3,NiCe}* + H^*_{hCe} \rightleftharpoons CH^*_{4,NiCe} + *_{hCe}$
$CH_2O^*_{NiCe} + H^*_{hO} \rightleftharpoons CH_3O^*_{NiCe} + *_{hO}$
$CH_2O^*_{NiCe}* + *_{NiO} \rightleftharpoons O^*_{NiCe} + *CH^*_{2,NiO}$
$HCO^*_{NiCe} + H^*_{hO} \rightleftharpoons CH_2O^*_{NiCe} + *_{hO}$
$HCO^*_{NiCe} + H^*_{hCe} \rightleftharpoons HCOH^*_{NiCe} + *_{hCe}$
$CH^*_{2,NiCe} + H^*_{hCe} \rightleftharpoons CH^*_{3,NiCe}* + *_{hCe}$
$CH^*_{2,NiCe} + H^*_{hO} \rightleftharpoons CH^*_{3,NiCe} + *_{hO}$
$CH^*_{NiCe}* + H^*_{hCe} \rightleftharpoons CH^*_{2,NiCe} + *_{hCe}$
$CH^*_{NiCe} + H^*_{hO} \rightleftharpoons CH^*_{2,NiCe} + *_{hO}$
$C^*_{NiCe}* + H^*_{hCe} \rightleftharpoons CH^*_{NiCe}$
$C^*_{NiCe} + H^*_{hO} \rightleftharpoons CH^*_{NiCe}$
$HCO^*_{NiCe} + *_{NiO} \rightleftharpoons O^*_{Ce} + CH^*_{NiO}$
$CO^*_{NiCe} + *_{NiO} \rightleftharpoons C^*_{Ce} + O^*_{NiO}$
$CO^*_{NiCe} + H^*_{hO} \rightleftharpoons HCO^*_{NiCe}* + *_{hO}$
$HCOO^*_{NiCe} + *_{NiO} \rightleftharpoons HCO^*_{NiCe} + O^*_{NiO}$
$CO^*_{2,NiCe} + H^*_{hO} \rightleftharpoons HCOO^*_{Ce} + *_{hO}$
$CO^*_{2,NiCe} + *_{NiO} \rightleftharpoons CO^*_{NiCe} + O^*_{NiO}$
$CH_{4(g)} + *_{NiCe} \rightleftharpoons CH_{4},NiCe^*$
$H_2O_{(g)} + *_{NiCe} \rightleftharpoons H_2O^*_{NiCe}$
$CO_{(g)} + *_{NiCe} \rightleftharpoons CO^*_{NiCe}$
$CO_{2(g)} + *_{NiCe} \rightleftharpoons CO^*_{2,NiCe}$
0.2534
3.5140
3.3361
0.0002
0.0675
0.1711
0.0045
0.0045
0.9323
0.1141
0.0182
0.0018
0.0004
0.0004
0.2009
0.1963
1.1222
0.1711
3.5897
2.1624
1.1222
net balance
forward
reverse
10^{-2}
10^0
10^2
10^4
event · s^{-1} $site^{-1}$

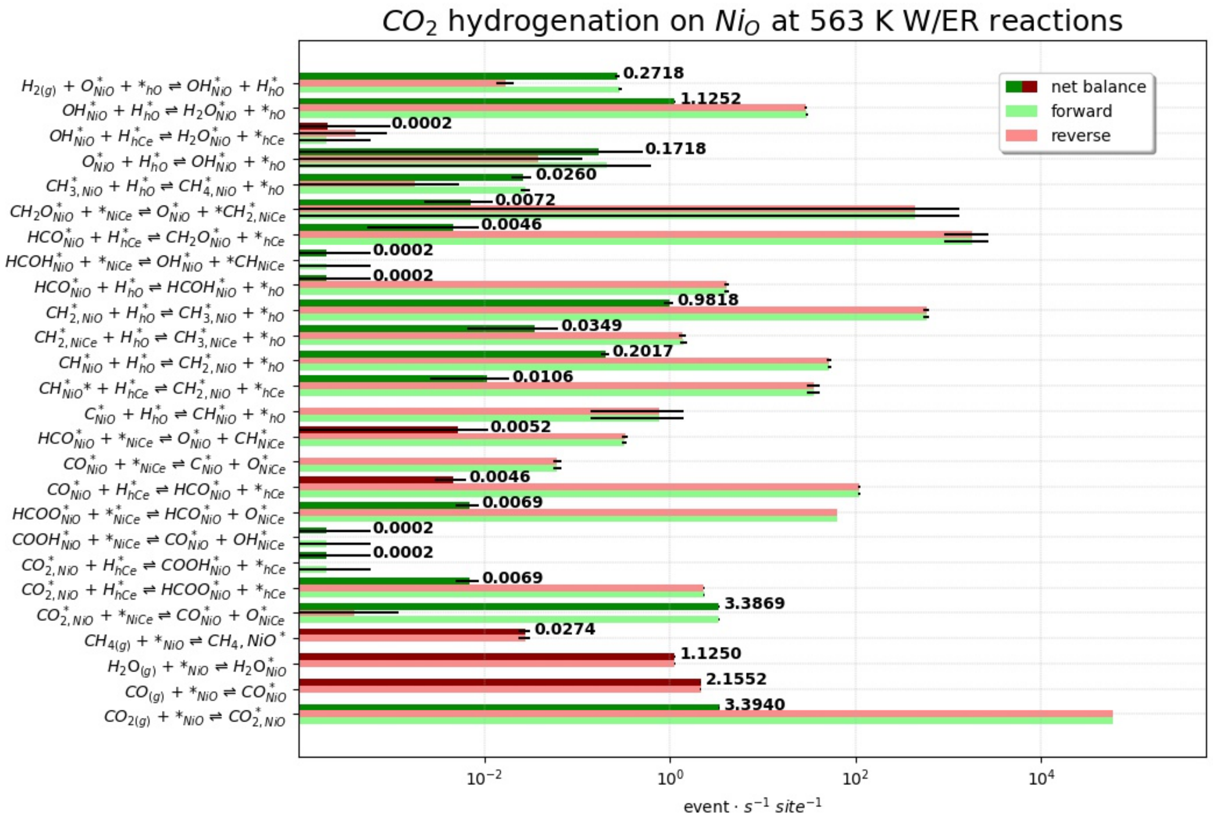
CO_2 hydrogenation on Ni_O at 563 K W/ER reactions
$H_{2(g)} + O^*_{NiO} + *_{hO} \rightleftharpoons OH^*_{NiO} + H^*_{hO}$
$OH^*_{NiO} + H^*_{hO} \rightleftharpoons H_2O^*_{NiO} + *_{hO}$
$OH^*_{NiO} + H^*_{hCe} \rightleftharpoons H_2O^*_{NiO} + *_{hCe}$
$O^*_{NiO} + H^*_{hO} \rightleftharpoons OH^*_{NiO} + *_{hO}$
$CH^*_{3,NiO} + H^*_{hO} \rightleftharpoons CH^*_{4,NiO} + *_{hO}$
$CH_2O^*_{NiO} + *_{NiCe} \rightleftharpoons O^*_{NiO} + *CH^*_{2,NiCe}$
$HCO^*_{NiO} + H^*_{hCe} \rightleftharpoons CH_2O^*_{NiO} + *_{hCe}$
$HCOH^*_{NiO} + *_{NiCe} \rightleftharpoons OH^*_{NiO} + *CH_{NiCe}$
$HCO^*_{NiO} + H^*_{hO} \rightleftharpoons HCOH^*_{NiO} + *_{hO}$
$CH^*_{2,NiO} + H^*_{hO} \rightleftharpoons CH^*_{3,NiO} + *_{hO}$
$CH^*_{2,NiCe} + H^*_{hO} \rightleftharpoons CH^*_{3,NiCe} + *_{hO}$
$CH^*_{NiO} + H^*_{hO} \rightleftharpoons CH^*_{2,NiO} + *_{hO}$
$CH^*_{NiO}* + H^*_{hCe} \rightleftharpoons CH^*_{2,NiO} + *_{hCe}$
$C^*_{NiO} + H^*_{hO} \rightleftharpoons CH^*_{NiO} + *_{hO}$
$HCO^*_{NiO} + *_{NiCe} \rightleftharpoons O^*_{NiO} + CH^*_{NiCe}$
$CO^*_{NiO} + *_{NiCe} \rightleftharpoons C^*_{NiO} + O^*_{NiCe}$
$CO^*_{NiO} + H^*_{hCe} \rightleftharpoons HCO^*_{NiO} + *_{hCe}$
$HCOO^*_{NiO} + *_{NiCe} \rightleftharpoons HCO^*_{NiO} + O^*_{NiCe}$
$COOH^*_{NiO} + *_{NiCe} \rightleftharpoons CO^*_{NiO} + OH^*_{NiCe}$
$CO^*_{2,NiO} + H^*_{hCe} \rightleftharpoons COOH^*_{NiO} + *_{hCe}$
$CO^*_{2,NiO} + H^*_{hCe} \rightleftharpoons HCOO^*_{NiO} + *_{hCe}$
$CO^*_{2,NiO} + *_{NiCe} \rightleftharpoons CO^*_{NiO} + O^*_{NiCe}$
$CH_{4(g)} + *_{NiO} \rightleftharpoons CH_{4},NiO^*$
$H_2O_{(g)} + *_{NiO} \rightleftharpoons H_2O^*_{NiO}$
$CO_{(g)} + *_{NiO} \rightleftharpoons CO^*_{NiO}$
$CO_{2(g)} + *_{NiO} \rightleftharpoons CO^*_{2,NiO}$
0.2718
1.1252
0.0002
0.1718
0.0260
0.0072
0.0046
0.0002
0.0002
0.9818
0.0349
0.2017
0.0106
0.0052
0.0046
0.0069
0.0002
0.0002
0.0069
3.3869
0.0274
1.1250
2.1552
3.3940
net balance
forward
reverse
10^{-2}
10^0
10^2
10^4
event · s^{-1} $site^{-1}$

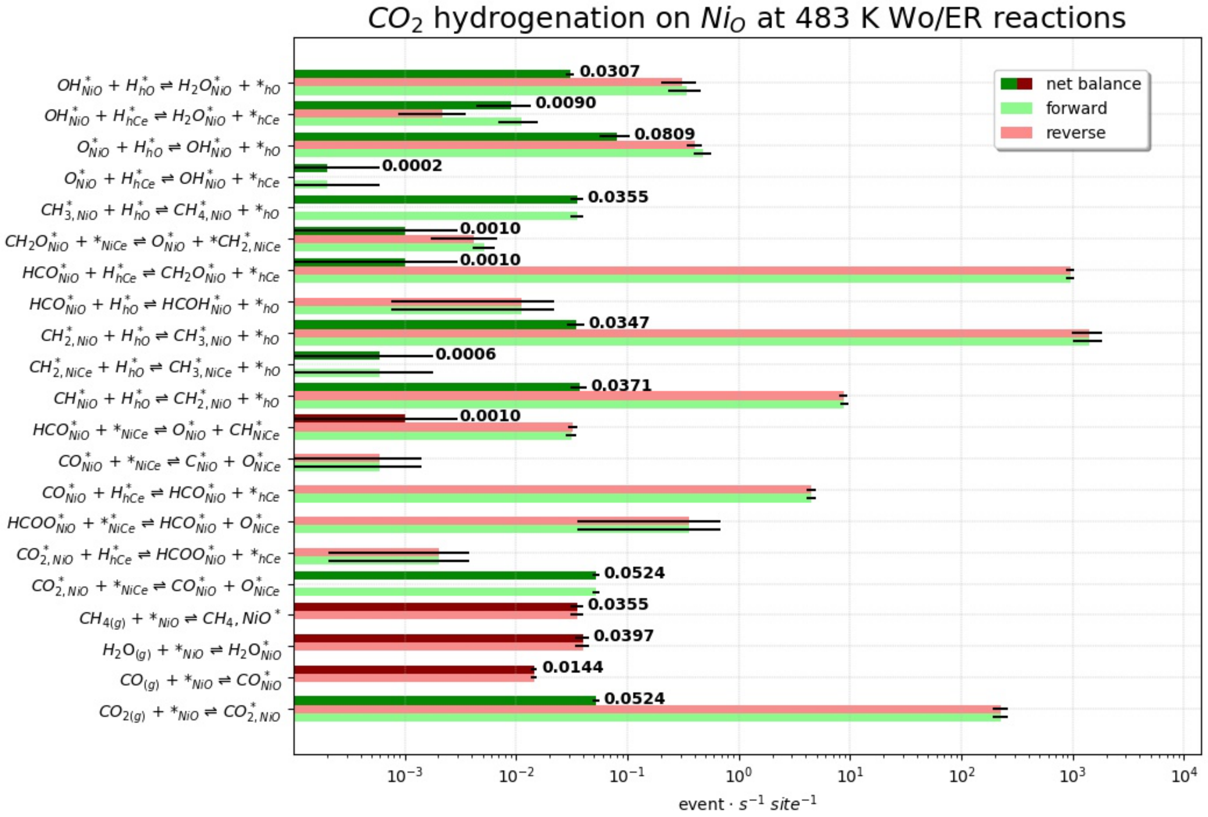
CO2 hydrogenation on NiO at 483 K Wo/ER reactions
net balance
forward
reverse
0.0307
0.0090
0.0809
0.0002
0.0355
0.0010
0.0010
0.0347
0.0006
0.0371
0.0010
0.0524
0.0355
0.0397
0.0144
0.0524
event · s−1 site−1

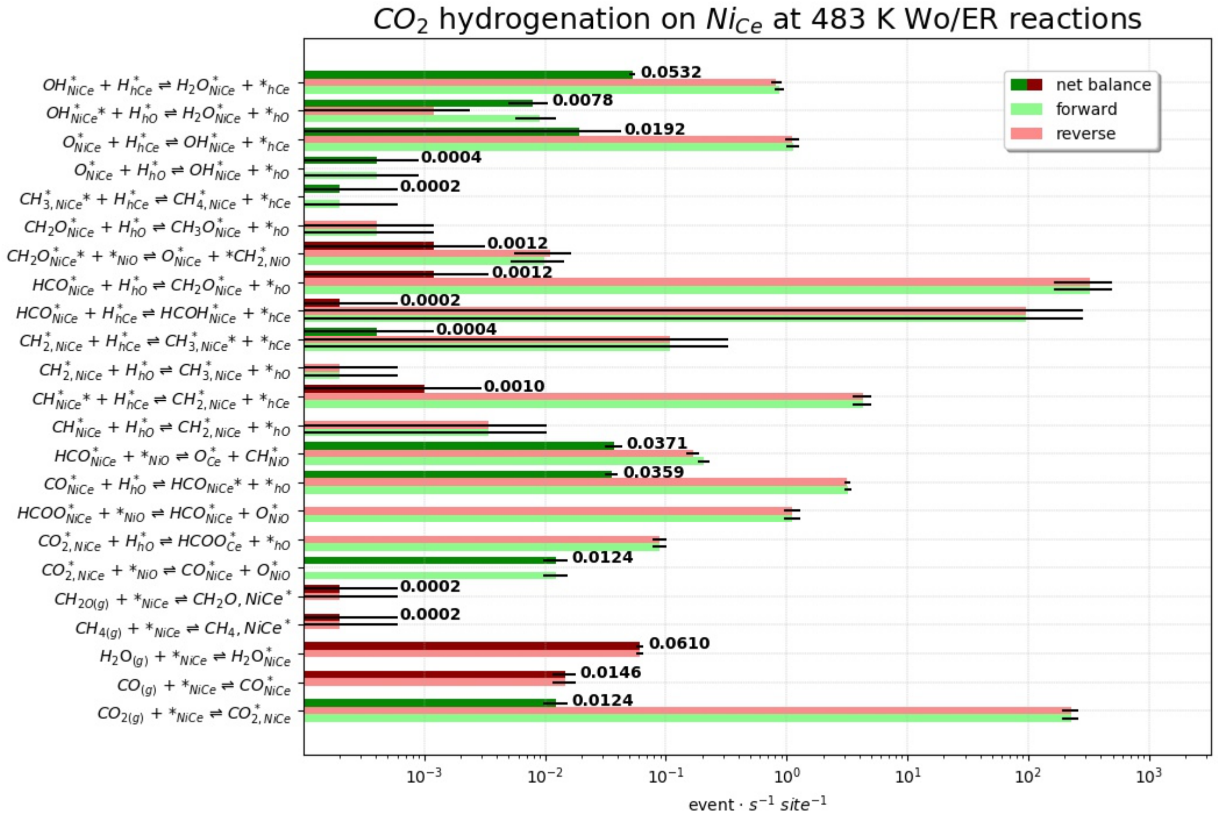
CO2 hydrogenation on NiCe at 483 K Wo/ER reactions
net balance
forward
reverse
0.0532
0.0078
0.0192
0.0004
0.0002
0.0012
0.0012
0.0002
0.0004
0.0010
0.0371
0.0359
0.0124
0.0002
0.0002
0.0610
0.0146
0.0124
event · s−1 site−1

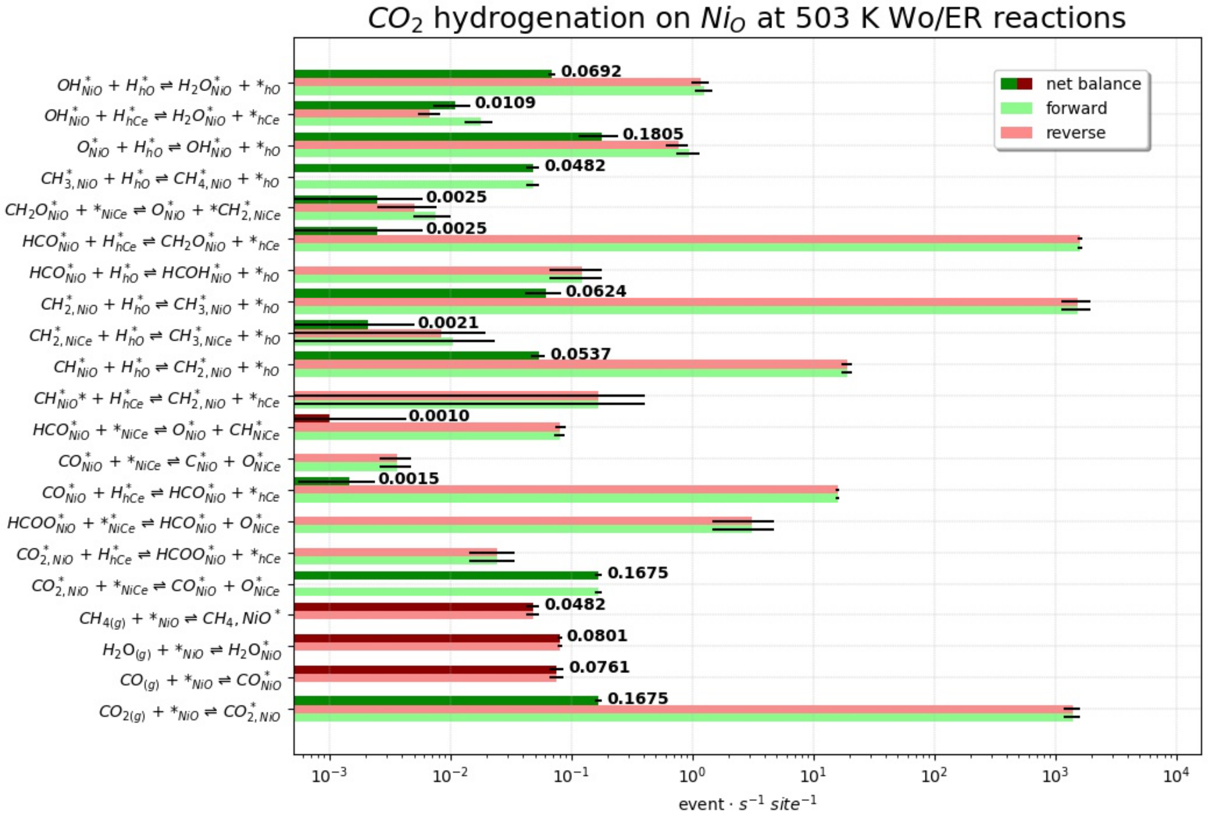
CO_2 hydrogenation on Ni_O at 503 K Wo/ER reactions
net balance
forward
reverse
event · s^{-1} $site^{-1}$

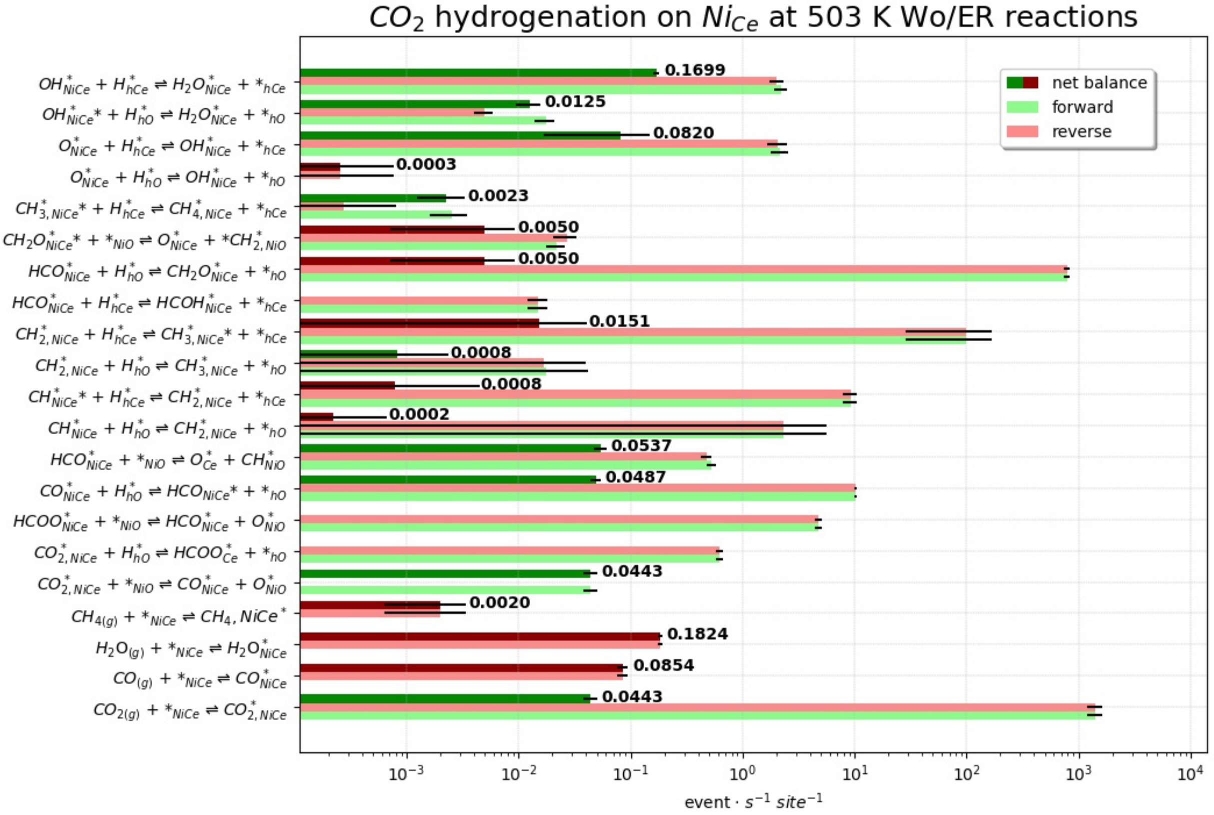
CO_2 hydrogenation on Ni_{Ce} at 503 K Wo/ER reactions
net balance
forward
reverse
event · s^{-1} $site^{-1}$

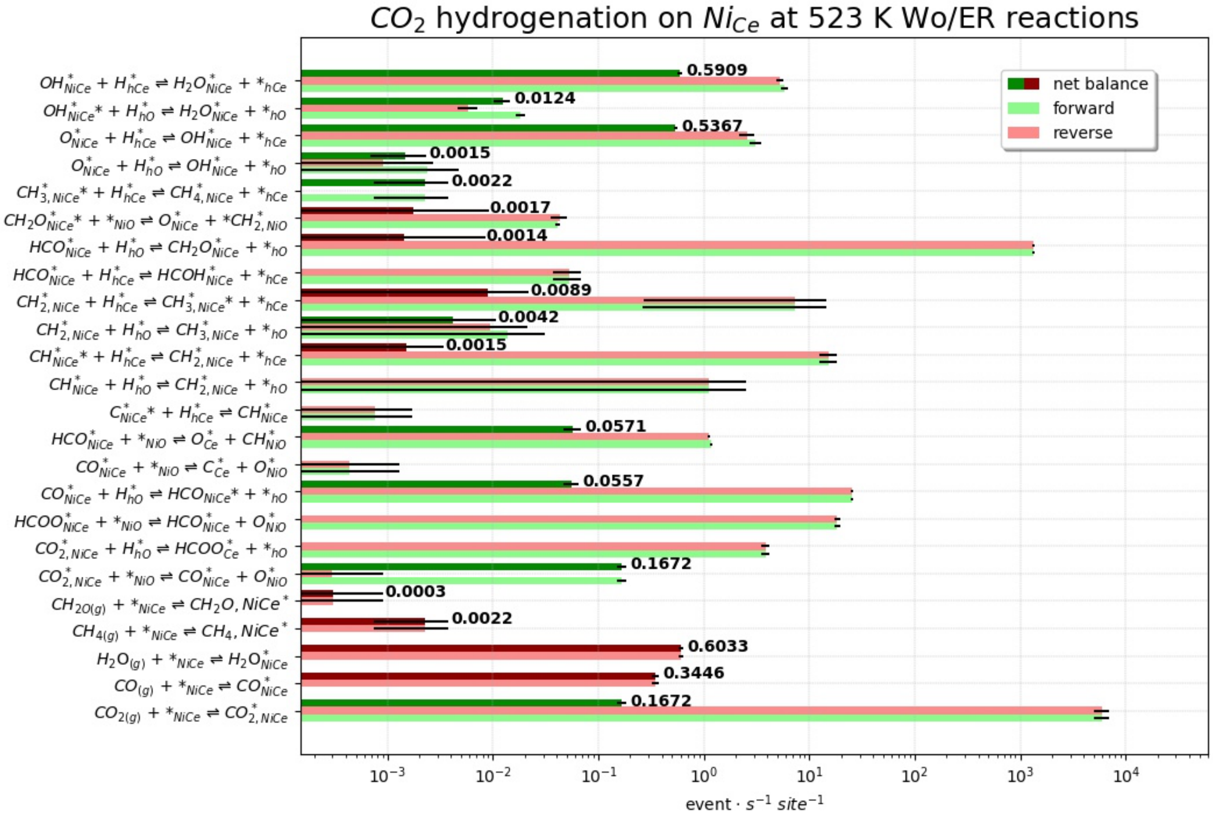
CO2 hydrogenation on NiCe at 523 K Wo/ER reactions
net balance
forward
reverse
0.5909
0.0124
0.5367
0.0015
0.0022
0.0017
0.0014
0.0089
0.0042
0.0015
0.0571
0.0557
0.1672
0.0003
0.0022
0.6033
0.3446
0.1672
event · s-1 site-1

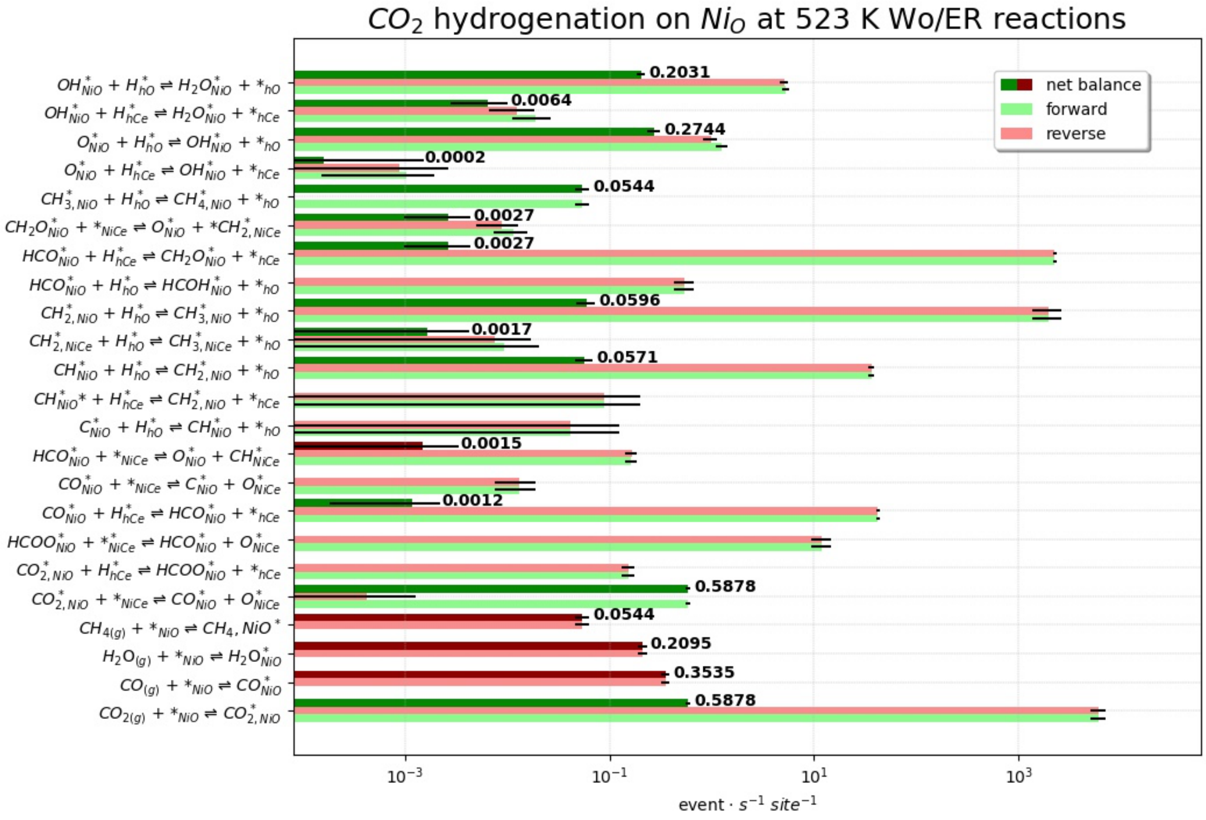
CO2 hydrogenation on NiO at 523 K Wo/ER reactions
net balance
forward
reverse
0.2031
0.0064
0.2744
0.0002
0.0544
0.0027
0.0027
0.0596
0.0017
0.0571
0.0015
0.0012
0.5878
0.0544
0.2095
0.3535
0.5878
event · s-1 site-1

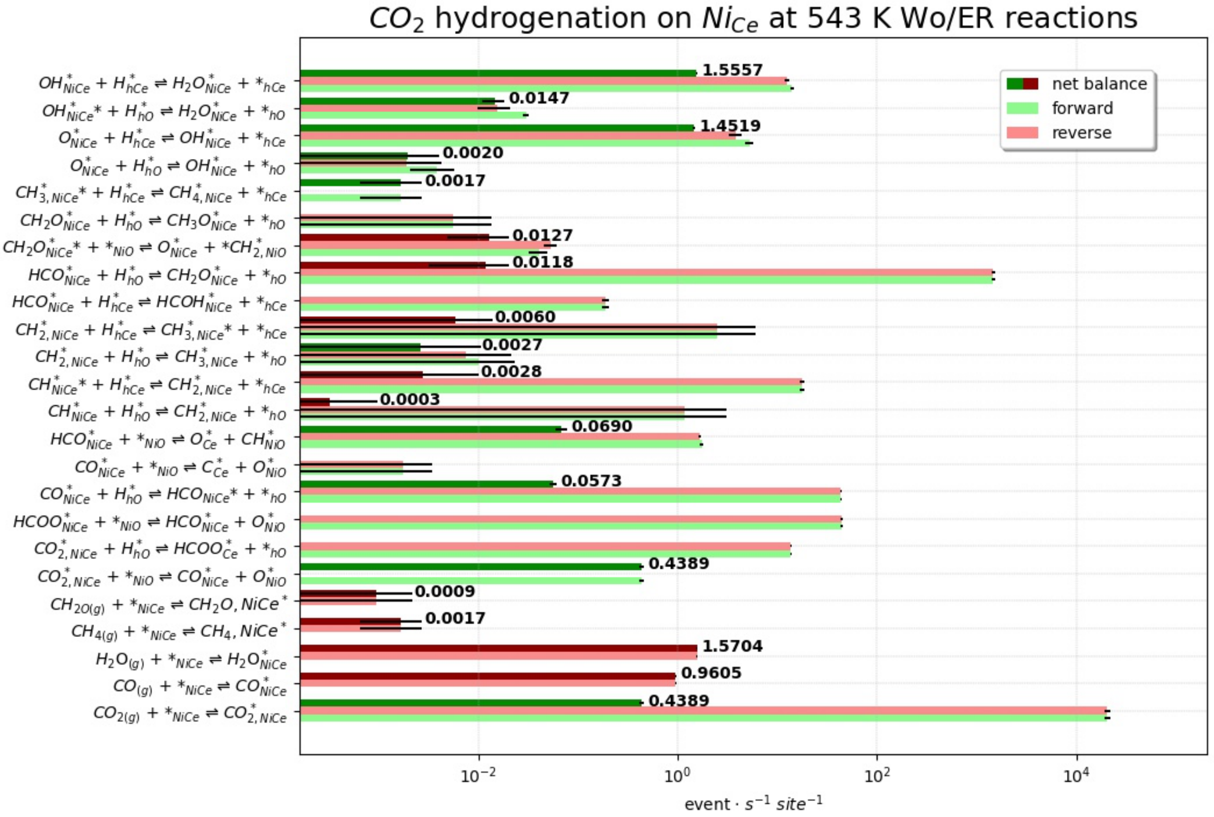
CO2 hydrogenation on NiCe at 543 K Wo/ER reactions
OH*NiCe + H*hCe ⇌ H2O*NiCe + *hCe
OH*NiCe* + H*hO ⇌ H2O*NiCe + *hO
O*NiCe + H*hCe ⇌ OH*NiCe + *hCe
O*NiCe + H*hO ⇌ OH*NiCe + *hO
CH*3,NiCe* + H*hCe ⇌ CH*4,NiCe + *hCe
CH2O*NiCe + H*hO ⇌ CH3O*NiCe + *hO
CH2O*NiCe* + *NiO ⇌ O*NiCe + *CH*2,NiO
HCO*NiCe + H*hO ⇌ CH2O*NiCe + *hO
HCO*NiCe + H*hCe ⇌ HCOH*NiCe + *hCe
CH*2,NiCe + H*hCe ⇌ CH*3,NiCe* + *hCe
CH*2,NiCe + H*hO ⇌ CH*3,NiCe + *hO
CH*NiCe* + H*hCe ⇌ CH*2,NiCe + *hCe
CH*NiCe + H*hO ⇌ CH*2,NiCe + *hO
HCO*NiCe + *NiO ⇌ O*Ce + CH*NiO
CO*NiCe + *NiO ⇌ C*Ce + O*NiO
CO*NiCe + H*hO ⇌ HCONiCe* + *hO
HCOO*NiCe + *NiO ⇌ HCO*NiCe + O*NiO
CO*2,NiCe + H*hO ⇌ HCOO*Ce + *hO
CO*2,NiCe + *NiO ⇌ CO*NiCe + O*NiO
CH2O(g) + *NiCe ⇌ CH2O, NiCe*
CH4(g) + *NiCe ⇌ CH4, NiCe*
H2O(g) + *NiCe ⇌ H2O*NiCe
CO(g) + *NiCe ⇌ CO*NiCe
CO2(g) + *NiCe ⇌ CO*2,NiCe
1.5557
0.0147
1.4519
0.0020
0.0017
0.0127
0.0118
0.0060
0.0027
0.0028
0.0003
0.0690
0.0573
0.4389
0.0009
0.0017
1.5704
0.9605
0.4389
net balance
forward
reverse
10^-2
10^0
10^2
10^4
event · s^-1 site^-1

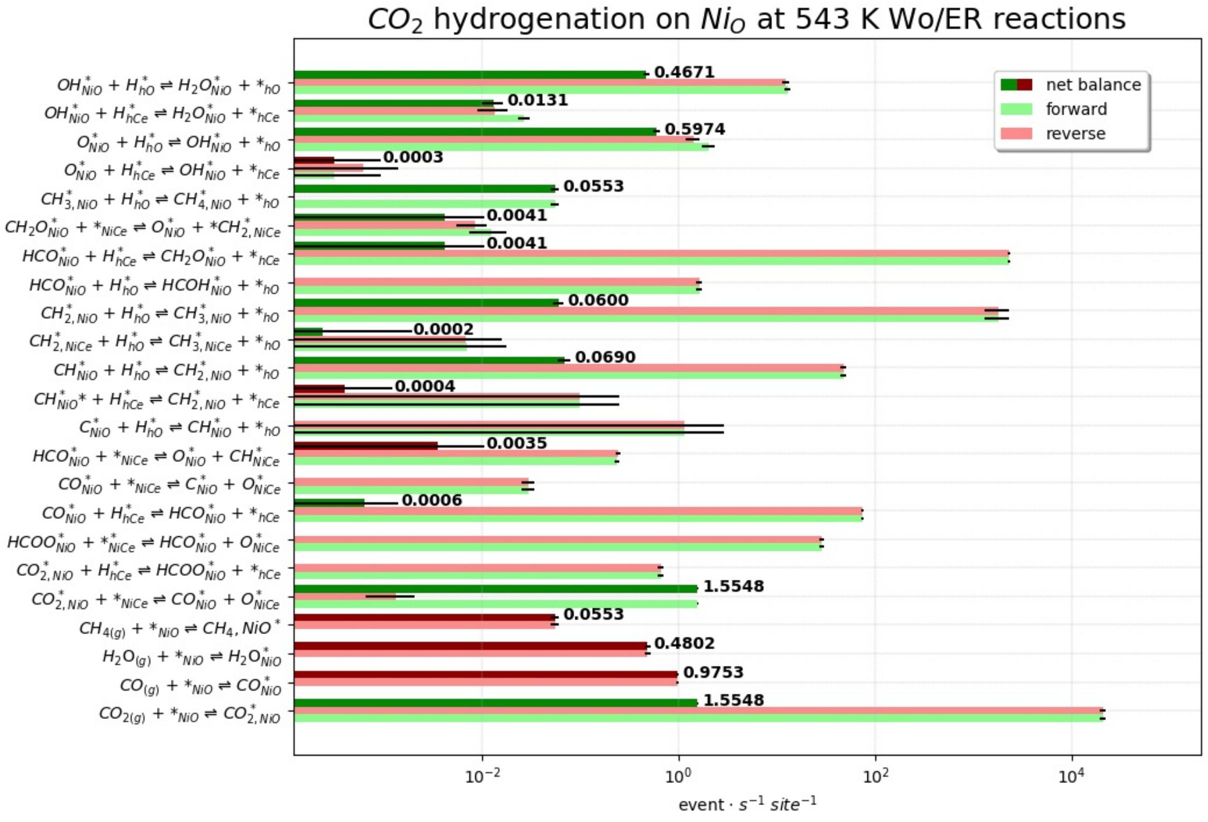
CO2 hydrogenation on NiO at 543 K Wo/ER reactions
OH*NiO + H*hO ⇌ H2O*NiO + *hO
OH*NiO + H*hCe ⇌ H2O*NiO + *hCe
O*NiO + H*hO ⇌ OH*NiO + *hO
O*NiO + H*hCe ⇌ OH*NiO + *hCe
CH*3,NiO + H*hO ⇌ CH*4,NiO + *hO
CH2O*NiO + *NiCe ⇌ O*NiO + *CH*2,NiCe
HCO*NiO + H*hCe ⇌ CH2O*NiO + *hCe
HCO*NiO + H*hO ⇌ HCOH*NiO + *hO
CH*2,NiO + H*hO ⇌ CH*3,NiO + *hO
CH*2,NiCe + H*hO ⇌ CH*3,NiCe + *hO
CH*NiO + H*hO ⇌ CH*2,NiO + *hO
CH*NiO* + H*hCe ⇌ CH*2,NiO + *hCe
C*NiO + H*hO ⇌ CH*NiO + *hO
HCO*NiO + *NiCe ⇌ O*NiO + CH*NiCe
CO*NiO + *NiCe ⇌ C*NiO + O*NiCe
CO*NiO + H*hCe ⇌ HCO*NiO + *hCe
HCOO*NiO + *NiCe ⇌ HCO*NiO + O*NiCe
CO*2,NiO + H*hCe ⇌ HCOO*NiO + *hCe
CO*2,NiO + *NiCe ⇌ CO*NiO + O*NiCe
CH4(g) + *NiO ⇌ CH4, NiO*
H2O(g) + *NiO ⇌ H2O*NiO
CO(g) + *NiO ⇌ CO*NiO
CO2(g) + *NiO ⇌ CO*2,NiO
0.4671
0.0131
0.5974
0.0003
0.0553
0.0041
0.0041
0.0600
0.0002
0.0690
0.0004
0.0035
0.0006
1.5548
0.0553
0.4802
0.9753
1.5548
net balance
forward
reverse
10^-2
10^0
10^2
10^4
event · s^-1 site^-1

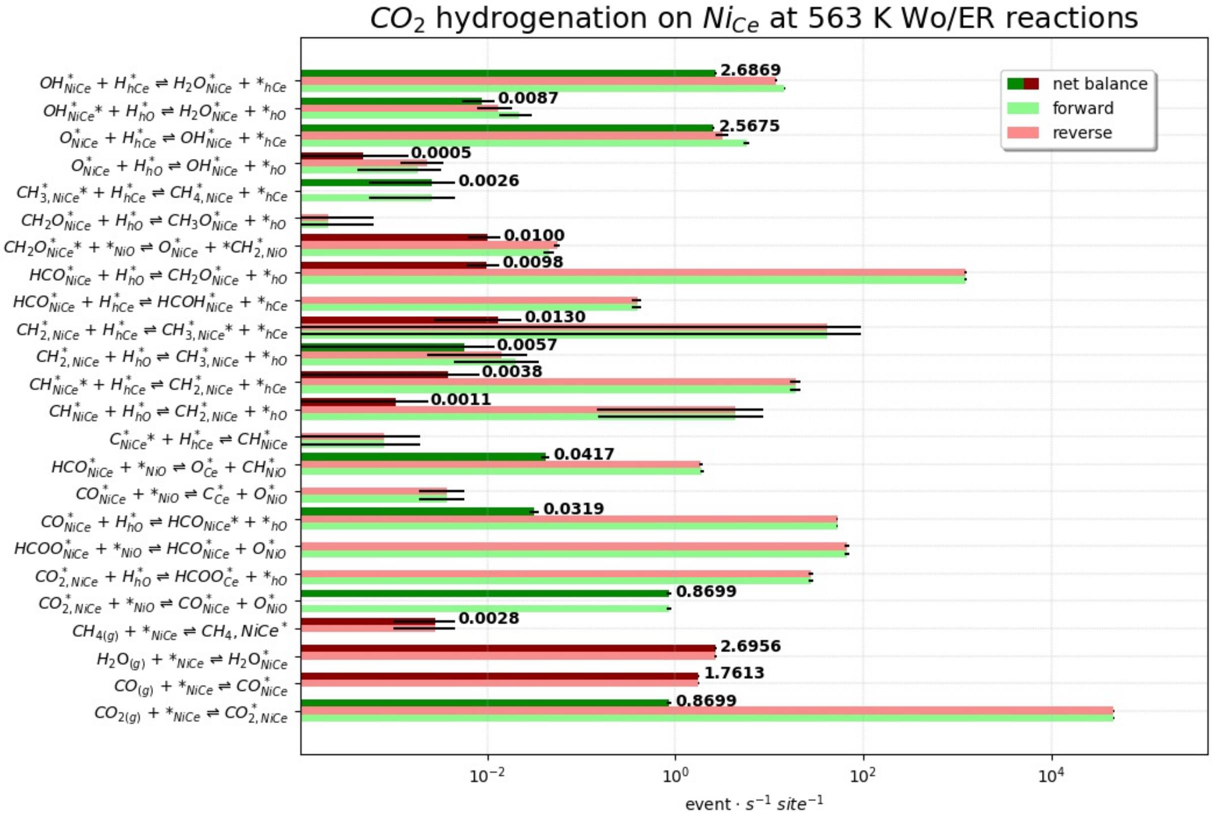
CO2 hydrogenation on NiCe at 563 K Wo/ER reactions
net balance
forward
reverse
2.6869
0.0087
2.5675
0.0005
0.0026
0.0100
0.0098
0.0130
0.0057
0.0038
0.0011
0.0417
0.0319
0.8699
0.0028
2.6956
1.7613
0.8699
event · s−1 site−1

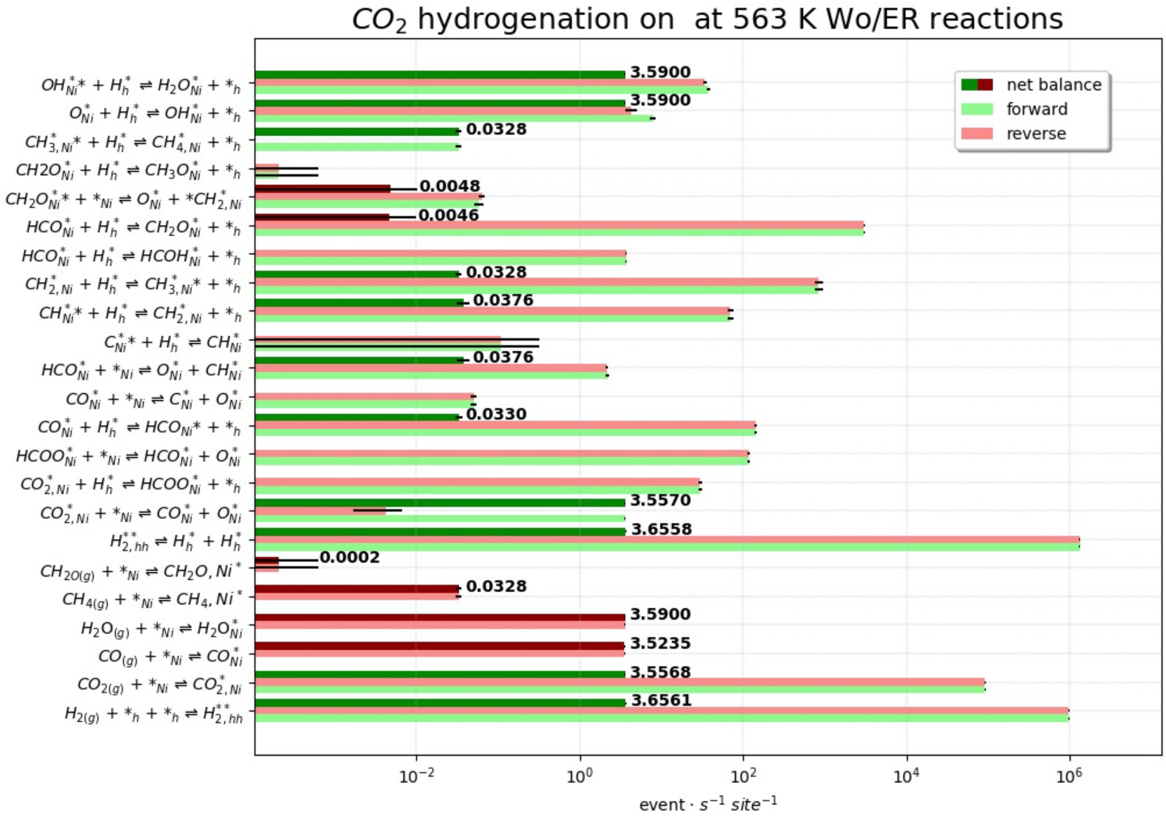
CO2 hydrogenation on at 563 K Wo/ER reactions
net balance
forward
reverse
3.5900
3.5900
0.0328
0.0048
0.0046
0.0328
0.0376
0.0376
0.0330
3.5570
3.6558
0.0002
0.0328
3.5900
3.5235
3.5568
3.6561
event · s−1 site−1

Figure S1. Plots Summarizing the Event Frequencies for the Different Sites at the Different Temperature Conditions and with P(H_2) = 0.528 bar and P(CO_2) = 0.132 bar, for the Simulations with the ER reactions (W/ER) and without the ER reactions (Wo/ER), respectively. Light Green and Light Red Stand for the Event Frequency for the Forward and Reverse Reactions, Respectively. The Net Balance of the Reaction is Shown by the Dark Color, Depicted by the Dark Green Color for a Net Forward Balance and Dark Red Color for a Net Reverse Balance. Bold Numbers Represent the Number of events s^{-1} $site^{-1}$ Taken as the Average of 5 Different kMC Simulations at the Same Conditions and Being the Black Lines, the Standard Deviation Associated.

REFERENCES

1. Lozano-Reis, P.; Prats, H.; Gamallo, P.; Illas, F.; Sayós R. Multiscale Study of the Mechanism of Catalytic CO_2 Hydrogenation: Role of the Ni(111) Facets. *ACS Catal.,* **2020**, 10, 8077–8089.
